# Crystal structures of three 4-methyl­piperidinium salts (one as three polymorphs) with tetrahalogenidoaurate(III), halide and (in one case) di­chloro­iodate(I) counter-anions

**DOI:** 10.1107/S2056989025004918

**Published:** 2025-06-12

**Authors:** Cindy Döring, Peter G. Jones

**Affiliations:** a Institut für Anorganische und Analytische Chemie, Technische Universität Braunschweig, Hagenring 30, D-38106 Braunschweig, Germany; Universität Greifswald, Germany

**Keywords:** crystal structure, tetra­halogenidoaurate(III), hydrogen bond, halogen bond, coinage bond

## Abstract

The structures of three 4-methyl­pyridinium tetra­halogenidoaurate(III) halides, one also including a di­chloro­iodate(I) anion, are presented. Bis(4-methyl­piperidinium) tetra­bromido­aurate(III) bromide, (4-Me-pipH)_2_[AuBr_4_]Br, **1**, crystallizes in space group *C*2/*c* with *Z* = 4. The gold atom lies on the inversion centre 0.75, 0.75, 0.5 and the bromide ion on the twofold axis 0.5, *y*, 0.25. Bis(4-methyl­piperidinium) tetra­chlorido­aurate(III) chloride, (4-Me-pipH)_2_[AuCl_4_]Cl, **2**, was obtained as three polymorphs, none of which is isotypic to **1**. Polymorph **2a** crystallizes in space group *P*2_1_/*c* with *Z* = 4; all atoms lie on general positions. Polymorph **2b** crystallizes in space group *P*2/*c* with *Z* = 8; two chloride ions lie on the twofold axes 0, *y*, 1/4 and 0.5, *y*, 0.25. Polymorph **2c** crystallizes in space group *P*1 with *Z* = 10; all atoms lie on general positions. Hexa­kis­(4-methyl­piperidinium) tetra­kis­{tetra­chlorido­aurate(III)} di­chloro­iodate(I) chloride, (4-Me-pipH)_6_[AuCl_4_]_4_(ICl_2_)Cl, **3**, crystallizes in space group *P*1 with *Z* = 1; two gold atoms occupy inversion centres, as do the iodine atom and one chloride. This is the first time that we have observed the chlorinating agent PhICl_2_ to be ‘non-innocent’. The main inter­est centres on the crystal packings, which involve hydrogen, halogen and coinage bonds, and display prominent substructures, one type involving the cations and the halides, and the other type involving the anions. A common feature in the packing of compounds **1** and **2** is a chain consisting of cations linked by halide ions. In **1**, the chains are flanked by tetra­bromido­aurate ions *via* short Br⋯Br contacts. The anions form zigzag chains *via* Br⋯Br^−^⋯Br groupings. In polymorph **2a**, chains of cations and chloride ions connect to chains of tetra­chlorido­aurate ions to form a layer structure. In polymorph **2b**, cation/chloride chains connect with chains of tetra­chlorido­aurate and chloride anions to form a layer structure; approximately linear Au⋯Cl^−^⋯Au groupings are a striking feature (these are also seen in **2c** and **3**). The asymmetric unit of polymorph **2c** is an ensemble *ca* 27 Å long. Cation/chloride chains are linked by tetra­chlorido­aurates to form one-dimensional polymers. The tetra­chlorido­aurate and chloride ions associate to form a layer structure. In the packing of compound **3** the chloride/tetra­chlorido­aurate substructure is dominant. Two tetra­chlorido­aurate ions associate with the free chloride to form a layer structure; the layers are linked in the third dimension *via* the third tetra­chloro­aurate and the di­chloro­iodate ions. There is no independent cation/chloride substructure; the cations may be considered as being inter­spersed in the spaces of the anionic substructure, forming hydrogen bonds (some multicentred) to chlorine atoms of the anions. A brief database survey of other structures involving aryl- or alkyl­ammonium cations, tetra­halogenidoaurates and halides is presented, including packing diagrams of selected examples.

## Abstract 2

The structures of three 4-methyl­pyridinium tetra­halogenidoaurate(III) halides, one also including a di­chloro­iodate(I) anion, are presented. The crystal packings involve hydrogen, halogen and coinage bonds, and display prominent substructure types involving the cations and halides or the anions alone.

## 1. Chemical context

In this series of publications, we have structurally investigated several classes of amine complexes of gold(I) and gold(III) halides, whereby the term ‘amine’ has been used loosely to include aza­aromatics. The gold(I) derivatives were often synthesized by the reaction of the ligand with chlorido- or bromido­(tetra­hydro­thio­phene)­gold(I), from which the tetra­hydro­thio­phene ligand is easily replaced. Oxidation to the gold(III) species was achieved using elemental bromine or the chlorine equivalent iodo­phenyl dichloride PhICl_2_. Extensive background material is given in Part 12 of this series (Döring & Jones, 2023 ▸).

One of the problems in these syntheses is the sensitivity of some products to hydrolysis and to traces of H^+^, so that crystallizations, which often take weeks or months, can lead to salts of the protonated amine with tetra­halogenidoaurates(III). This tendency is exacerbated by the tendency of the frequently used solvent di­chloro­methane to react with amines, even in the absence of any other species (*e.g.* with pyridine; Rudine *et al.*, 2010 ▸). The structures of the isolated salts have however often proved to be inter­esting in their own right; for instance, they often exhibit short halogen⋯halogen contacts between tetra­halogenidoaurate(III) ions, sometimes leading to networks of these ions (Döring & Jones, 2016 ▸; this publication was not assigned a series number).
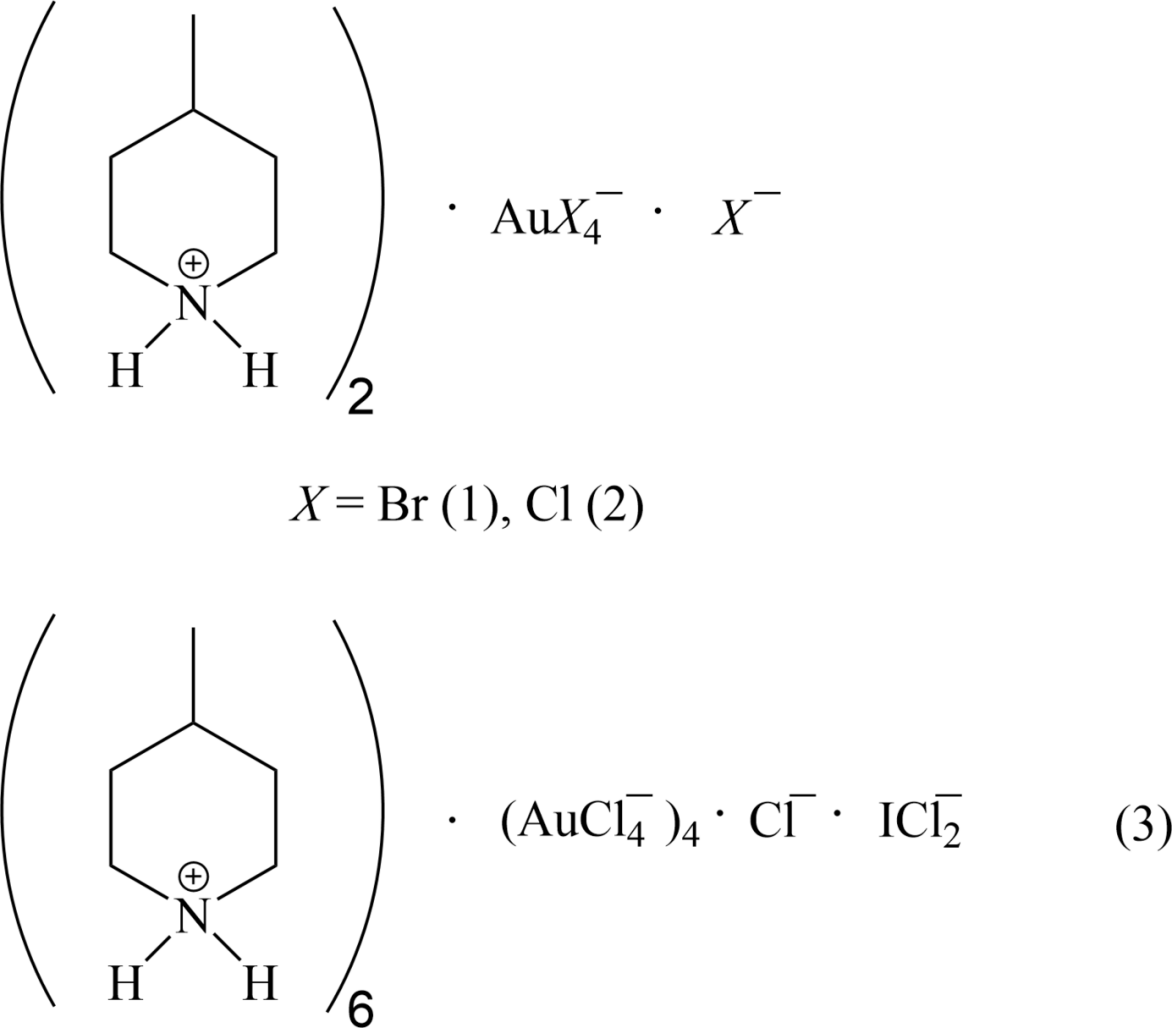


**Figure 1:**
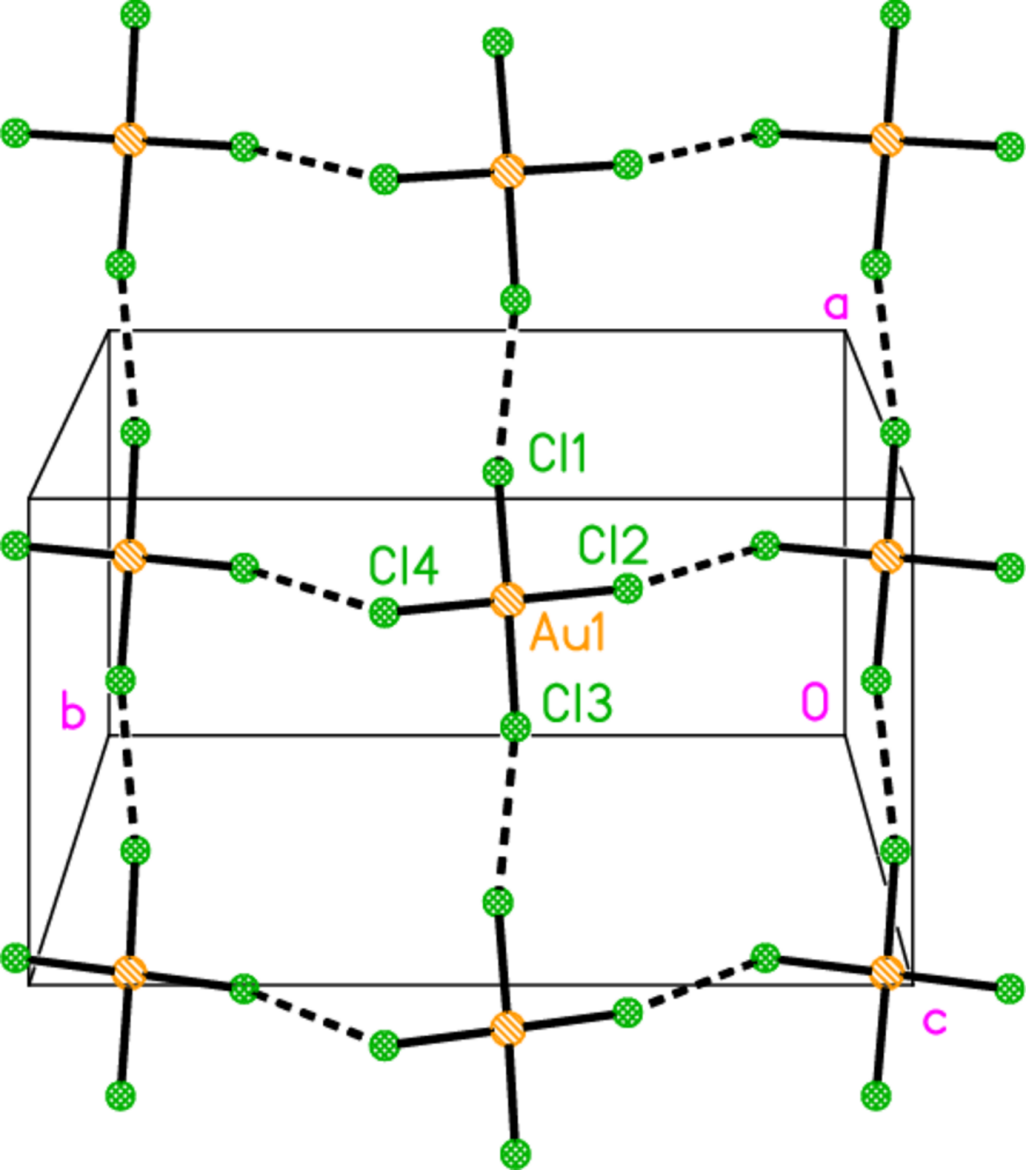
The approximately square network of tetra­chlorido­aurate ions in the compound (4-Me-pipH)[AuCl_4_] (Döring & Jones, 2016 ▸). The dashed bonds indicate Cl⋯Cl contacts.

In the series of 4-methyl­piperidine (4-Me-pip) complexes, we have previously determined the structures of bis­(4-methyl­piperidine)­gold(I) chloride, [Au(4-Me-pip)_2_]Cl; bis­(4-methyl­piperidine)­gold(I) di­chlorido­aurate(I), [Au(4-Me-pip)_2_] [AuCl_2_]; bis­(4-methyl­piperidine)­gold(I) di­bromido­aurate(I), [Au(4-Me-pip)_2_] [AuBr_2_]; the 1:1 adduct chlorido­(4-methyl­piperidine)­gold(I) bis­(4-methyl­piperidine)­gold(I) chlor­ide, AuCl(4-Me-pip)·[Au(4-Me-pip)_2_]Cl, as its di­chloro­methane solvate (Döring & Jones, 2024*a* ▸); tri­chlorido­(4-Me-pip)gold(III), (4-Me-pip)AuCl_3_; tri­bromido­(4-Me-pip)gold(III), (4-Me-pip)AuBr_3_ (Döring & Jones, 2024*b* ▸); and 4-methyl­piperidinium tetra­chlorido­aurate(III), (4-Me-pipH)[AuCl_4_] (Döring & Jones, 2016 ▸). In the last of these papers, we presented the structures of six compounds for which the tetra­halogenidoaurate ions assembled to form approximately square networks with gold atoms at the corners and short halogen⋯halogen contacts Au—*X*⋯*X*—Au along the sides of the squares (Fig. 1 ▸). Here we present the structures of the more complex ionic systems bis­(4-methyl­piperidinium) tetra­bromido­aurate(III) bromide, (4-Me-pipH)_2_[AuBr_4_]Br **1**; bis­(4-methyl­piperidinium) tetra­chlorido­aurate(III) chloride, (4-Me-pipH)_2_[AuCl_4_]Cl **2** (three polymorphs); and hexa­kis­(4-methyl­piperidinium) tetra­kis­{tetra­chlorido­aurate(III)} di­chloro­iodate(I) chloride, (4-Me-pipH)_6_[AuCl_4_]_4_(ICl_2_)Cl **3**. The presence of both halide and tetra­halogenidoaurate ions extends the potential types of anion assemblies.

## 2. Structural commentary

**Figure 2:**
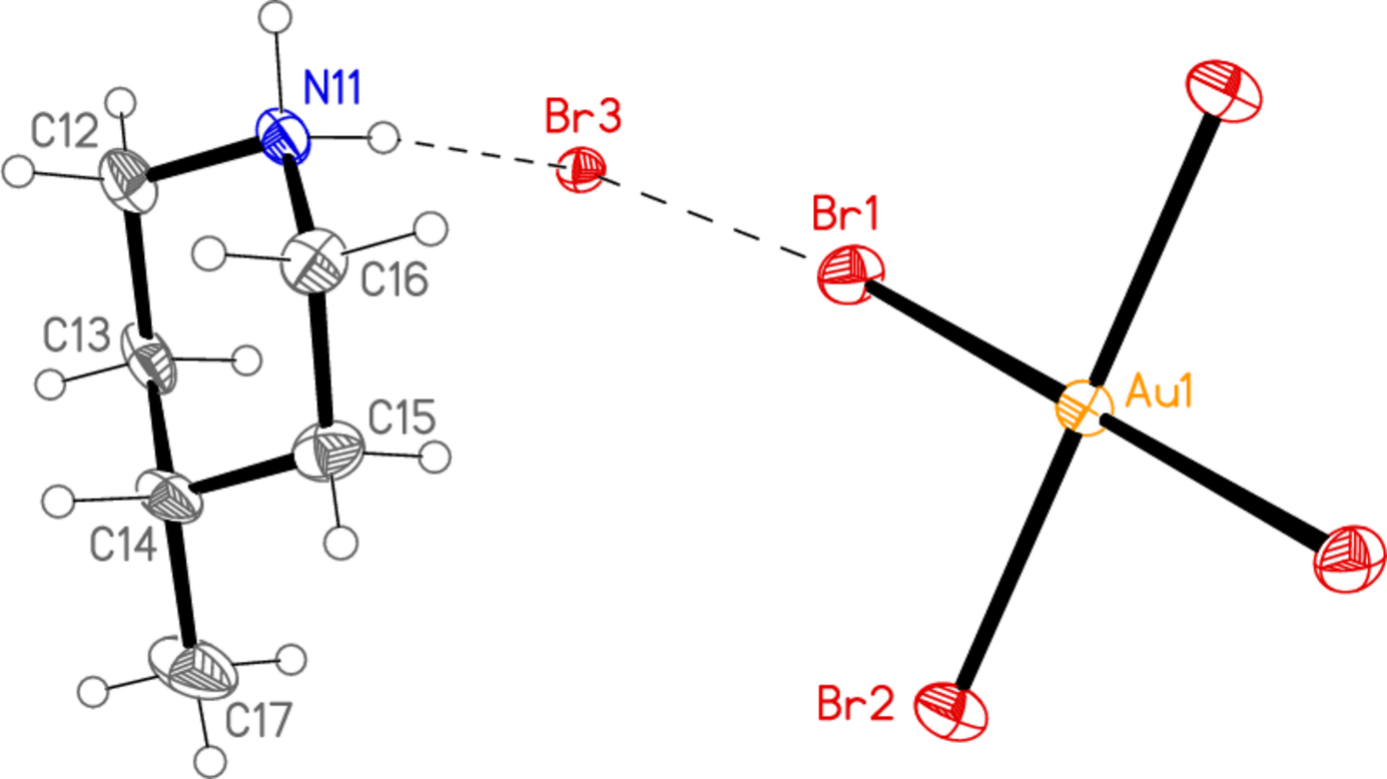
The formula unit of compound **1** in the crystal, extended by symmetry to complete the tetra­bromido­aurate ion. Only the asymmetric unit is labelled; ellipsoids represent 50% probability levels and the dashed lines represent short contacts that are discussed in *Supra­molecular features*. This also applies to Figs. 2 ▸–5 ▸ ▸ ▸.

**Figure 3:**
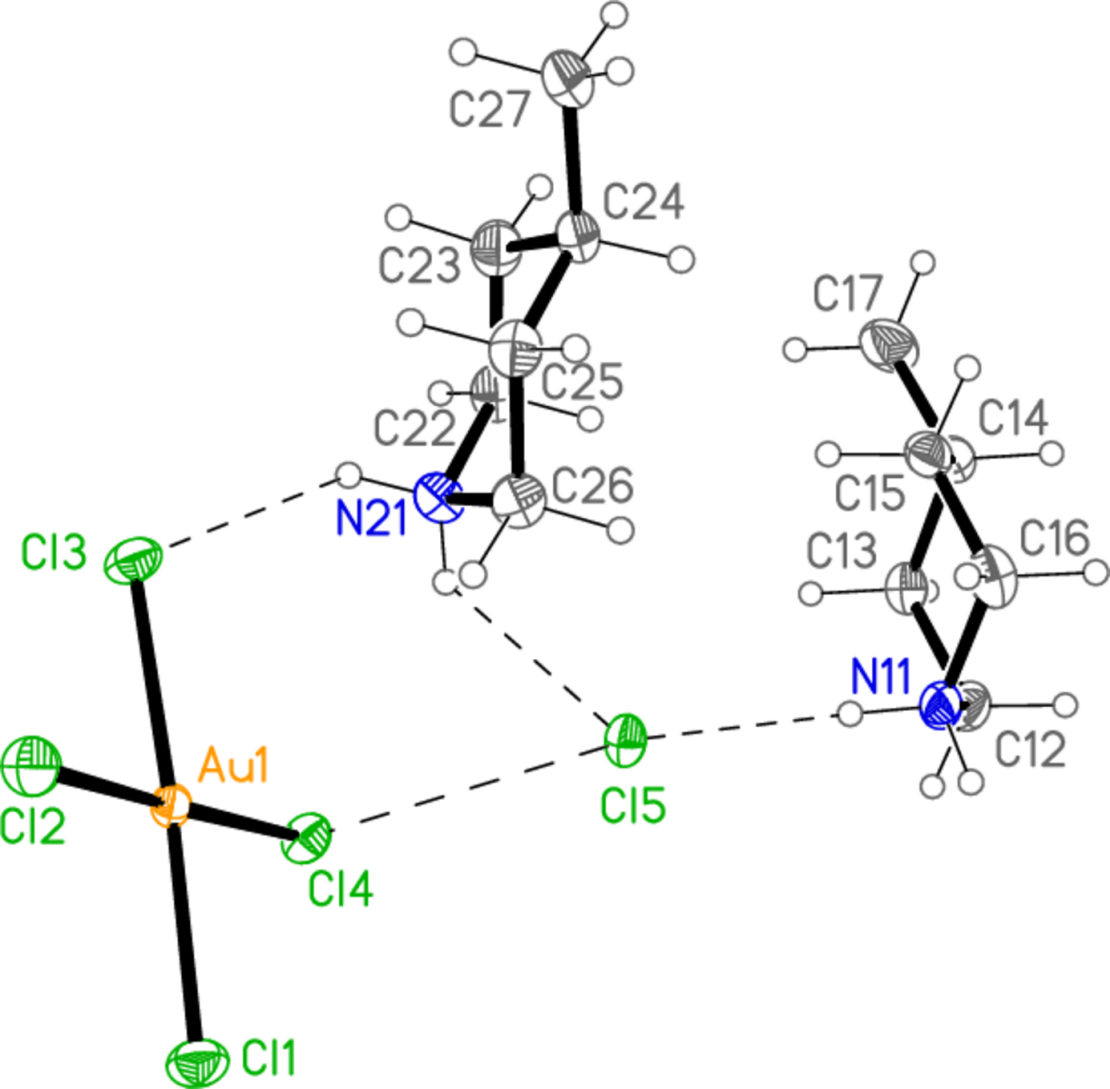
The formula unit of compound **2**, polymorph **2a**, in the crystal.

**Figure 4:**
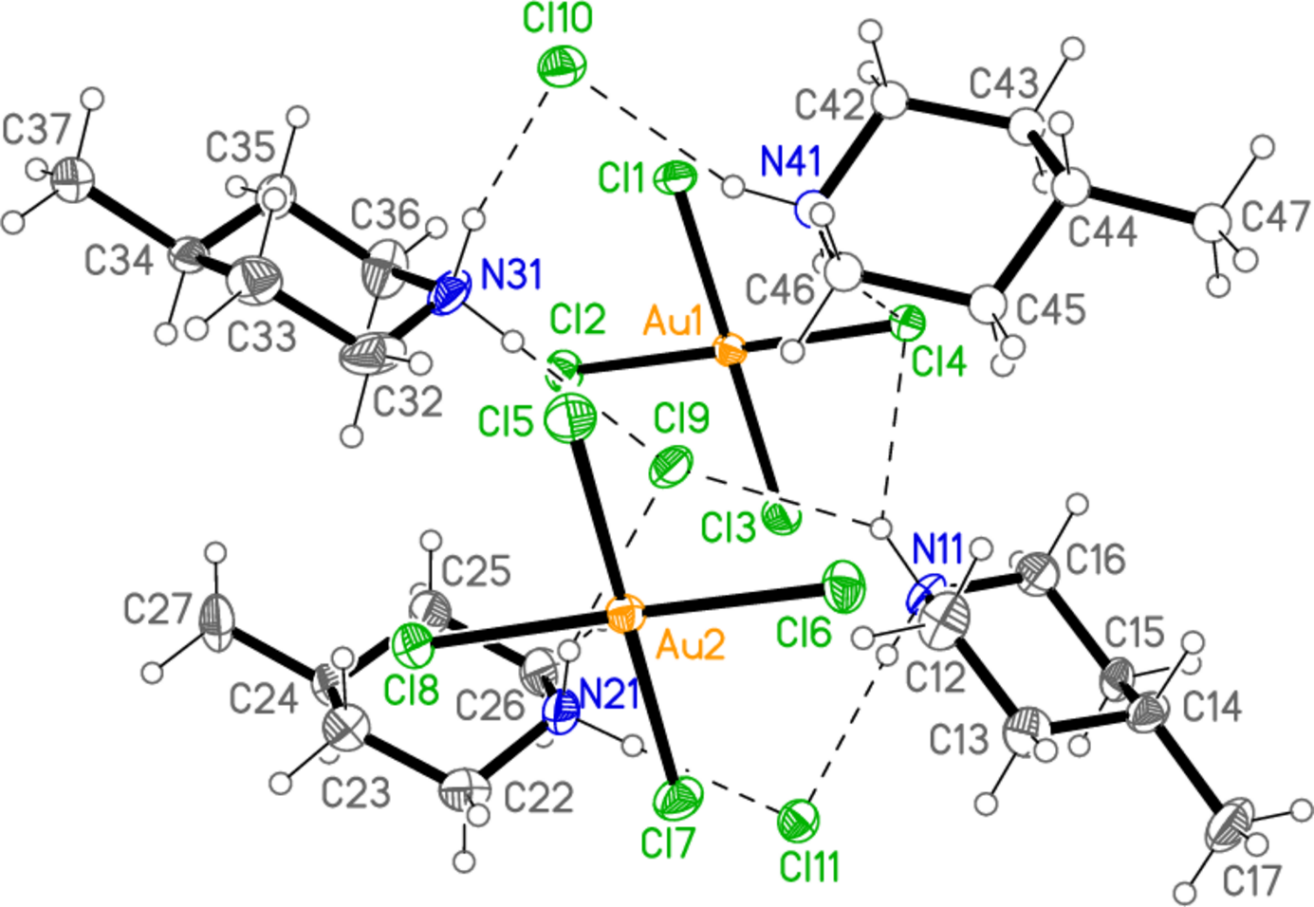
The formula unit of compound **2**, polymorph **2b**, in the crystal.

**Figure 5:**
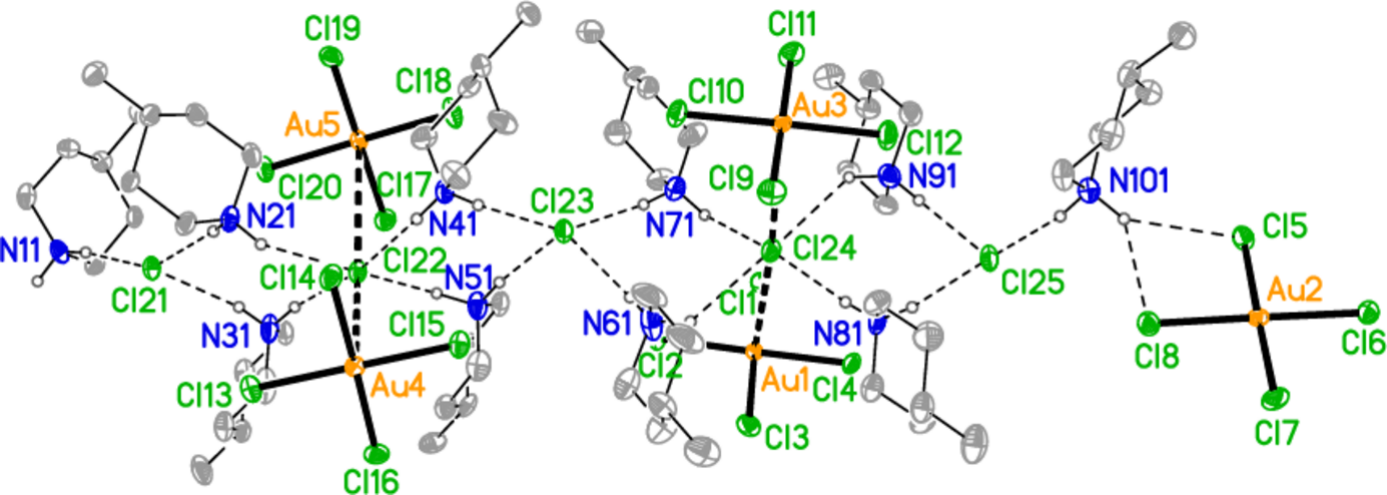
The formula unit of compound **2**, polymorph **2c**, in the crystal. For clarity, hydrogen atoms bonded to carbon have been omitted. Atoms Cl1, Cl2 and Cl18 are partially obscured. The borderline contact Cl20⋯Cl21 is excluded.

**Figure 6:**
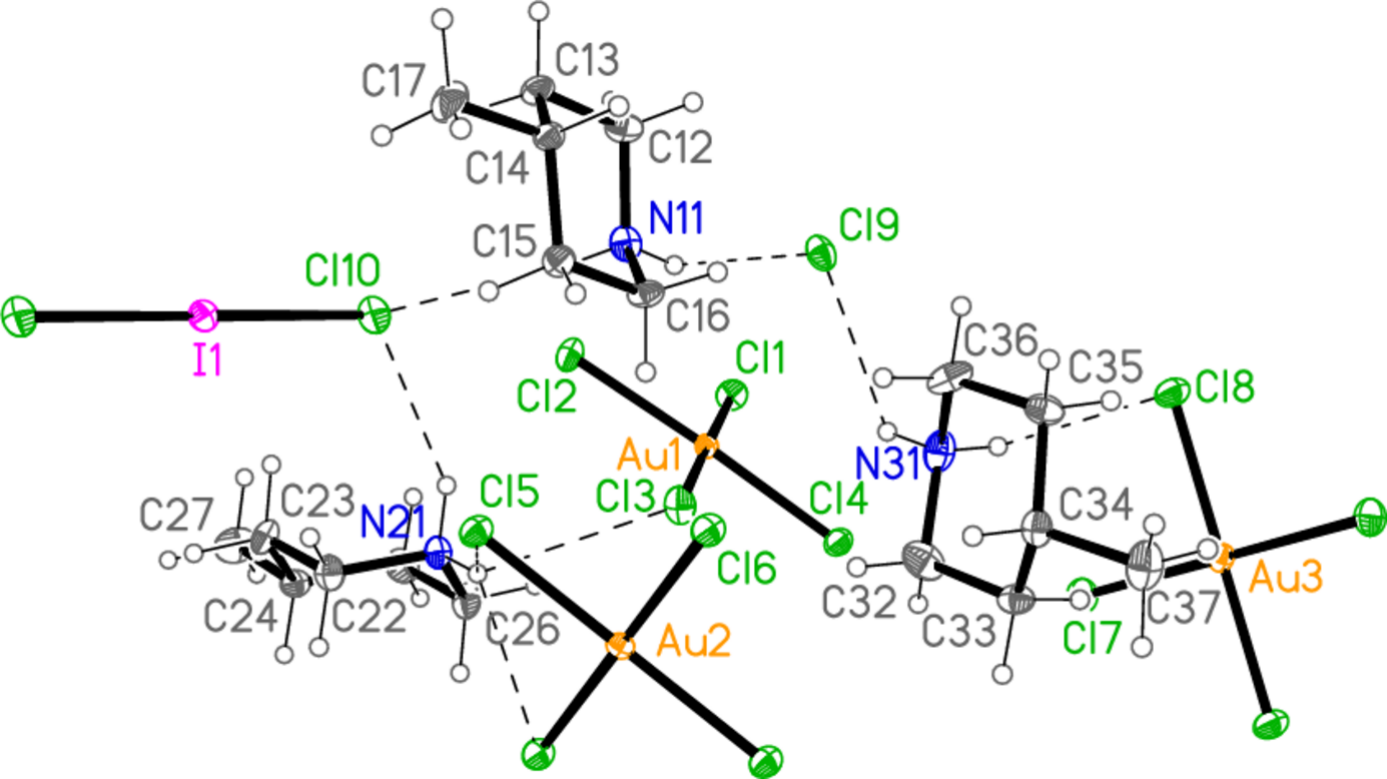
The formula unit of compound **3** in the crystal, extended by symmetry to complete the tetra­chlorido­aurate and di­chloro­iodate ions. Only the asymmetric unit is labelled.

All compounds crystallize solvent-free. In the Figures (Figs. 2 ▸–6 ▸ ▸ ▸ ▸), the asymmetric units have been extended by symmetry where necessary to show complete residues; the dashed lines indicate short contacts that are discussed in *Supra­molecular features*. Bis(4-methyl­piperidinium) tetra­bromido­aurate(III) bromide, (4-Me-pipH)_2_[AuBr_4_]Br **1** (Fig. 2 ▸) crystallizes in space group *C*2/*c* with *Z* = 4. The gold atom lies on the inversion centre 0.75, 0.75, 0.5 and the bromide ion on the twofold axis 0.5, *y*, 0.25. Bis(4-methyl­piperidinium) tetra­chlorido­aurate(III) chloride, (4-Me-pipH)_2_[AuCl_4_]Cl **2**, was obtained as three polymorphs, none of which is isotypic to **1**. Polymorph **2a** (Fig. 3 ▸) crystallizes in space group *P*2_1_/*c* with *Z* = 4; all atoms lie on general positions. Polymorph **2b** (Fig. 4 ▸) crystallizes in space group *P*2/*c* with *Z* = 8; two chloride ions lie on the twofold axes 0, *y*, 0.25 (Cl10) and 0.5, *y*, 0.25 (Cl11). Polymorph **2c** (Fig. 5 ▸) crystallizes in space group *P*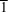 with *Z* = 10; all atoms lie on general positions. The asymmetric unit thus contains 20 residues. The chlorine atoms of the tetra­chlorido­aurate anions are numbered Cl1–Cl20 and the free chloride ions Cl21–Cl25. Hexa­kis­(4-methyl­piperidinium) tetra­kis­{tetra­chlorido­aurate(III)} di­chloro­iodate(I) chloride, (4-Me-pipH)_6_[AuCl_4_]_4_(ICl_2_)Cl **3** (Fig. 6 ▸) crystallizes in space group *P*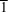 with *Z* = 1; two gold atoms occupy inversion centres, Au2 at 1, 0.5, 0.5 and Au3 at 0.5, 0, 0, as do the iodine atom I1, at 1, 1, 0.5, and one chloride, Cl9, at 0.5, 0.5, 0. This is the first time in our experience that the chlorinating agent PhICl_2_ has proved to be ‘non-innocent’.

Selected mol­ecular dimensions are shown in Tables 1 ▸–5 ▸ ▸ ▸ ▸. The tetra­halogenidoaurate(III) ions show the expected square-planar (4/*mmm*) symmetry to a good approximation, although there is some scatter of the Au—Cl bond lengths, which range from 2.2624 (13) to 2.3007 (8) Å. It is tempting to suggest that the differences are attributable to the short inter­ionic contacts, but no clear pattern can be discerned. In the cations, the methyl substituent is consistently equatorial, with C—C—C—C_meth­yl_ torsion angles around ±180°.

## 3. Supra­molecular features

In the packing diagrams, atom labels indicate atoms of the asymmetric unit (except where otherwise indicated). Hydrogen atoms of CH_2_ and CH groups are omitted (but their contacts are present in the deposited material); we subjectively assess the C—H⋯*X* contacts to be less important than N—H⋯*X*, although there are several of the former type, as would be expected in compounds with many more C—H than N—H moieties. In the text, primes (′) indicate previously defined or generalized symmetry operators. Classical hydrogen bonds are listed in Tables 6 ▸–10 ▸ ▸ ▸ ▸.

**Figure 7:**
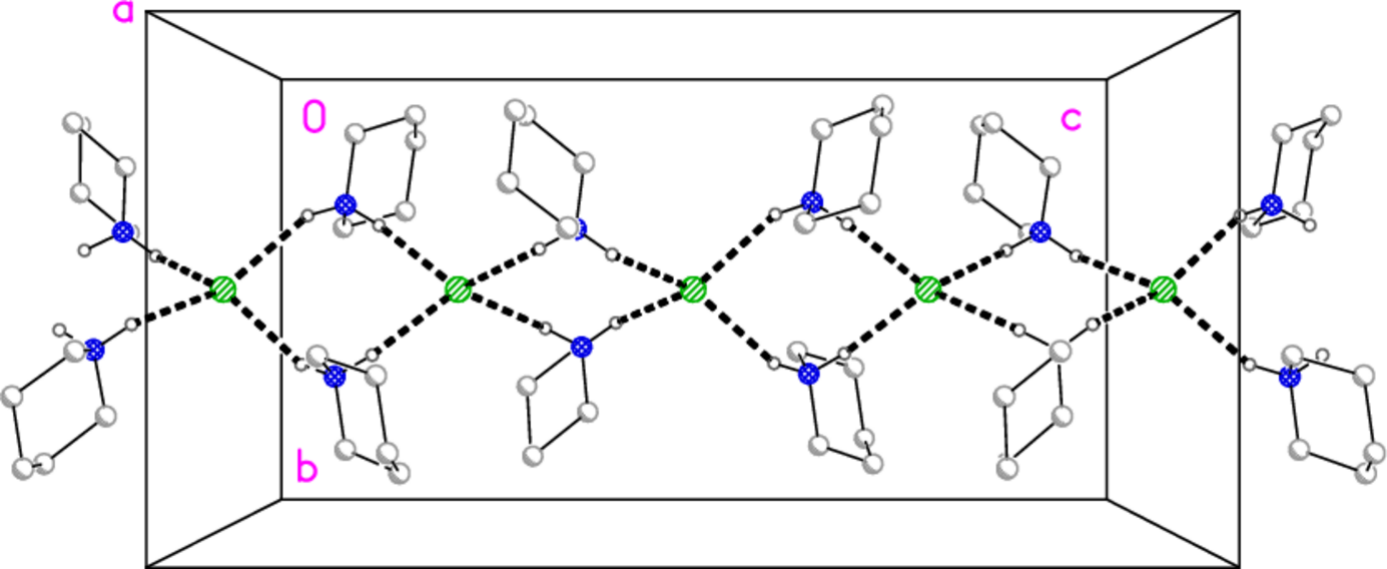
Packing diagram of the cations and chloride ions in the structure of (pipH)_2_[AuCl_4_]Cl (Döring & Jones, 2023 ▸); dashed lines indicate hydrogen bonds.

**Figure 8:**
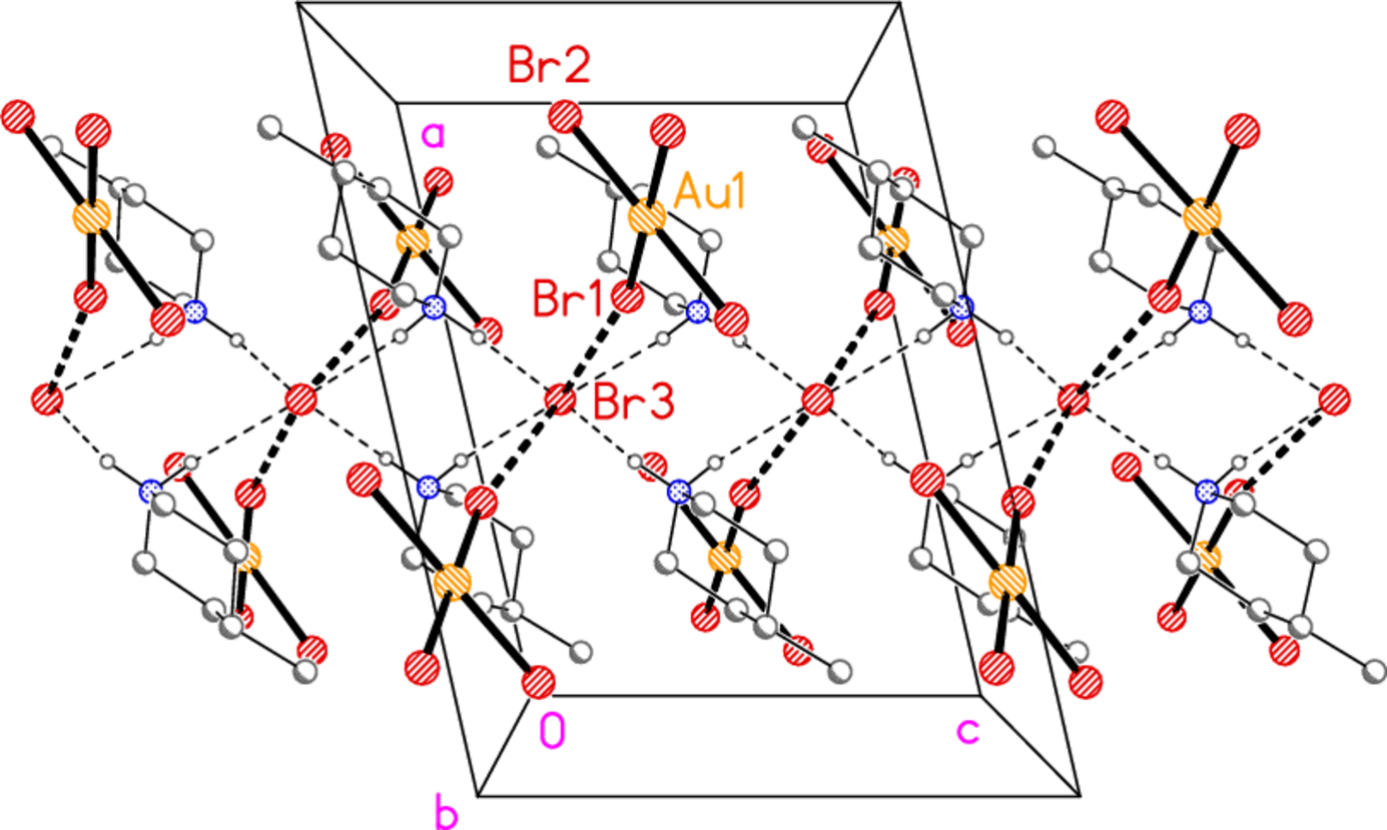
Packing diagram of compound **1** viewed parallel to the *b* axis. Thin dashed lines show H⋯Br hydrogen bonds; thick dashed lines show short Br⋯Br contacts.

**Figure 9:**
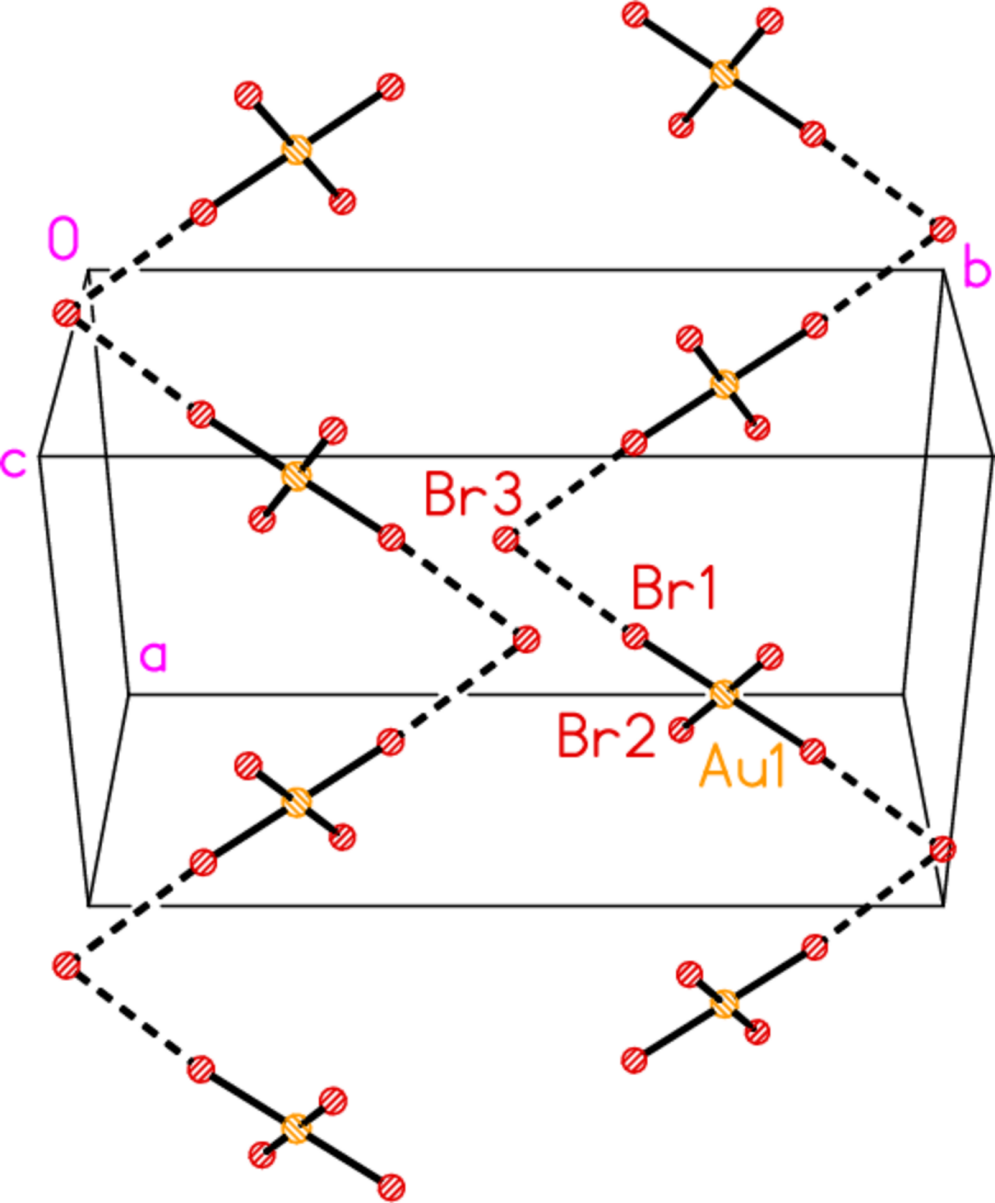
Packing diagram of the anion chains in compound **1**. The view direction is perpendicular to (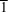
01).

**Figure 10:**
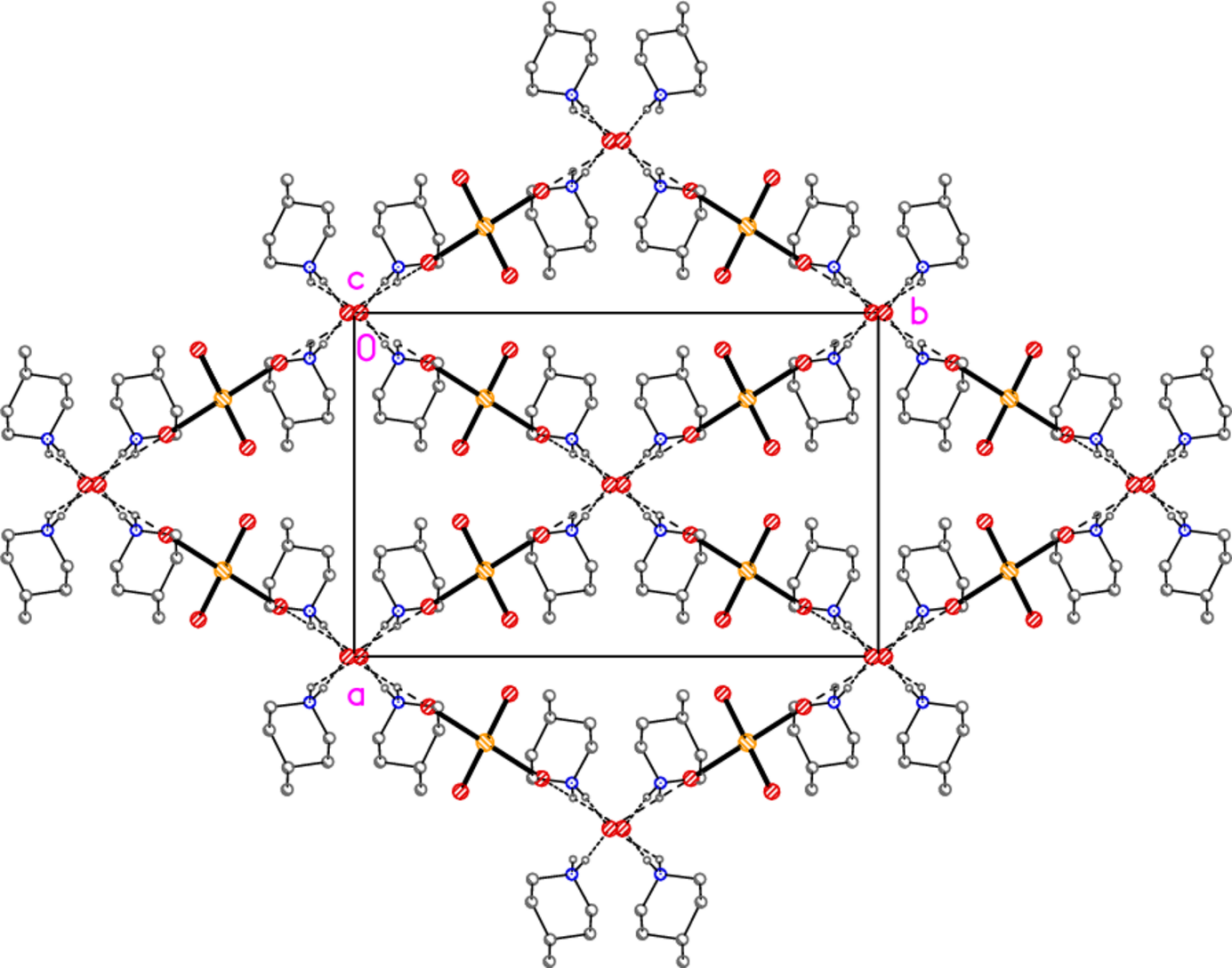
Packing diagram of compound **1** projected parallel to the *c* axis. Dashed lines indicate H⋯Br and Br⋯Br contacts.

A common feature in the packing of compounds **1** and **2** is a chain consisting of cations linked by halide ions. The closely related compounds (pipH)_2_[AuCl_4_]Cl and (pyrrolidinium)_2_[AuBr_4_]Br (Döring & Jones, 2023 ▸) both show related chains; the latter was shown in the original publication, but the former was not shown explicitly, so we provide it here (Fig. 7 ▸). The chains involve hydrogen-bonded rings, each with two cationic NH_2_ groups and two chloride ions, with graph set 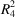
(8). These are connected by the apical chloride anions, which accept four hydrogen bonds, two from each of the two connected rings. The presence of an alkyl­ammonium-type cation is not a prerequisite for such chains; another example is the structure of bis­(cyclo­hexyl­amine)­gold(I) chloride (Döring & Jones, 2018 ▸), which has a formally uncharged NH_2_ group in the coordinated amine. The packing of compound **1** involves exactly analogous chains of NH_2_ groups and bromides, running parallel to the *c* axis (Fig. 8 ▸); each chain is flanked by tetra­bromido­aurate ions via short contacts Br3⋯Br1 of 3.6584 (7) Å, which can be classified as halogen bonds (for reviews see e.g. Metrangelo *et al.*, 2008 ▸ or Cavallo *et al.*, 2016 ▸). Fig. 9 ▸ shows the zigzag chains formed by the anions; the angle Br1⋯Br3⋯Br1(1 − *x*, *y*, 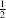 − *z*) is 74.38 (2)° and Au1—Br1⋯Br3′ is 168.10 (2)°. The chains propagate parallel to [101]. Fig. 10 ▸ shows a projection of the complete packing parallel to the *c* axis; the cation/bromide chains occupy the regions at the corners and the centre of the projected cell.

**Figure 11:**
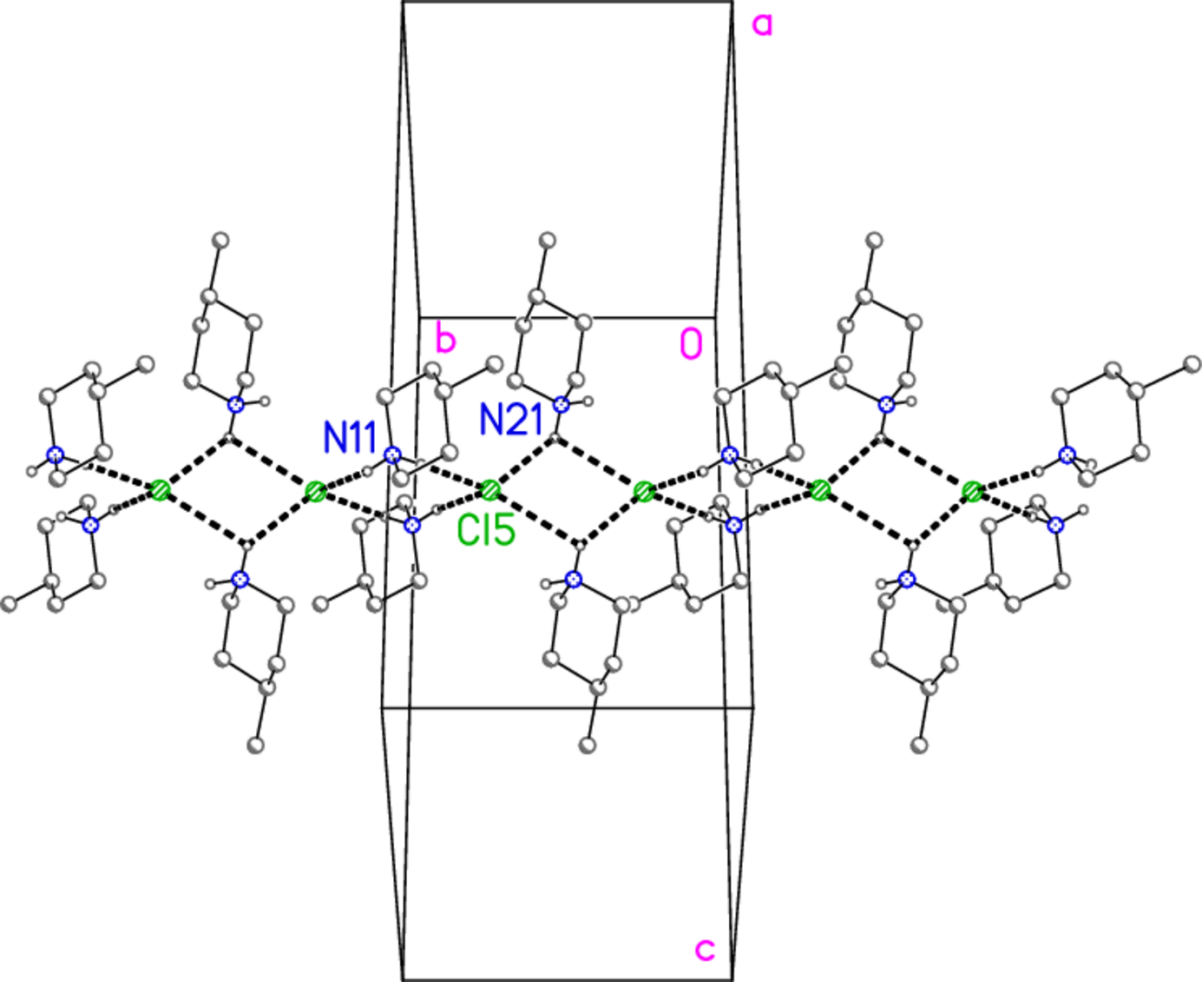
The cation/chloride chain of compound **2**, polymorph **2a**, viewed perpendicular to (101). Dashed lines indicate hydrogen bonds.

**Figure 12:**
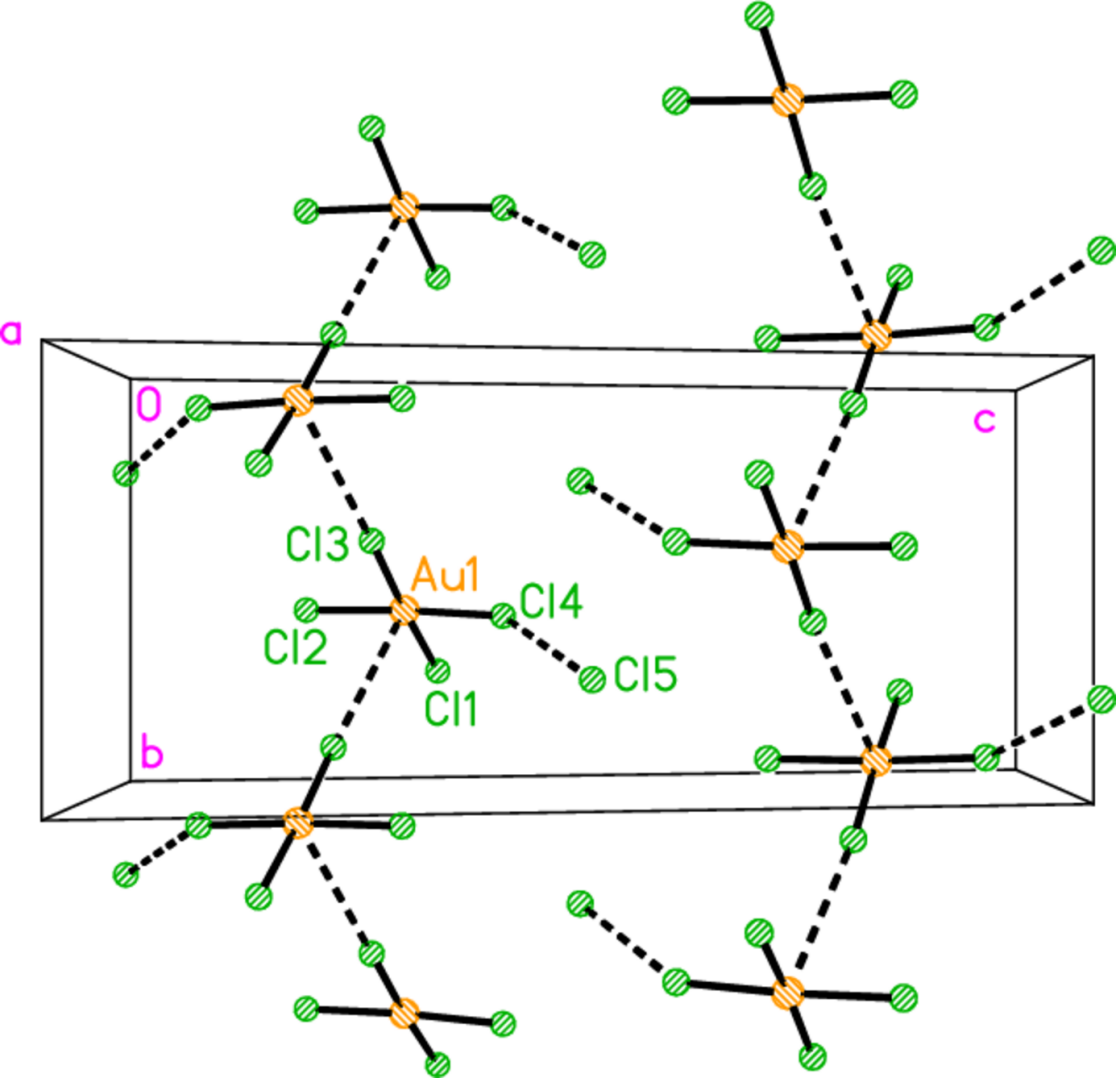
Two tetra­chlorido­aurate(III) chains of compound **2**, polymorph **2a**, with peripherally attached chloride ions, viewed parallel to the *a* axis. Dashed lines indicate Au⋯Cl or Cl⋯Cl contacts.

**Figure 13:**
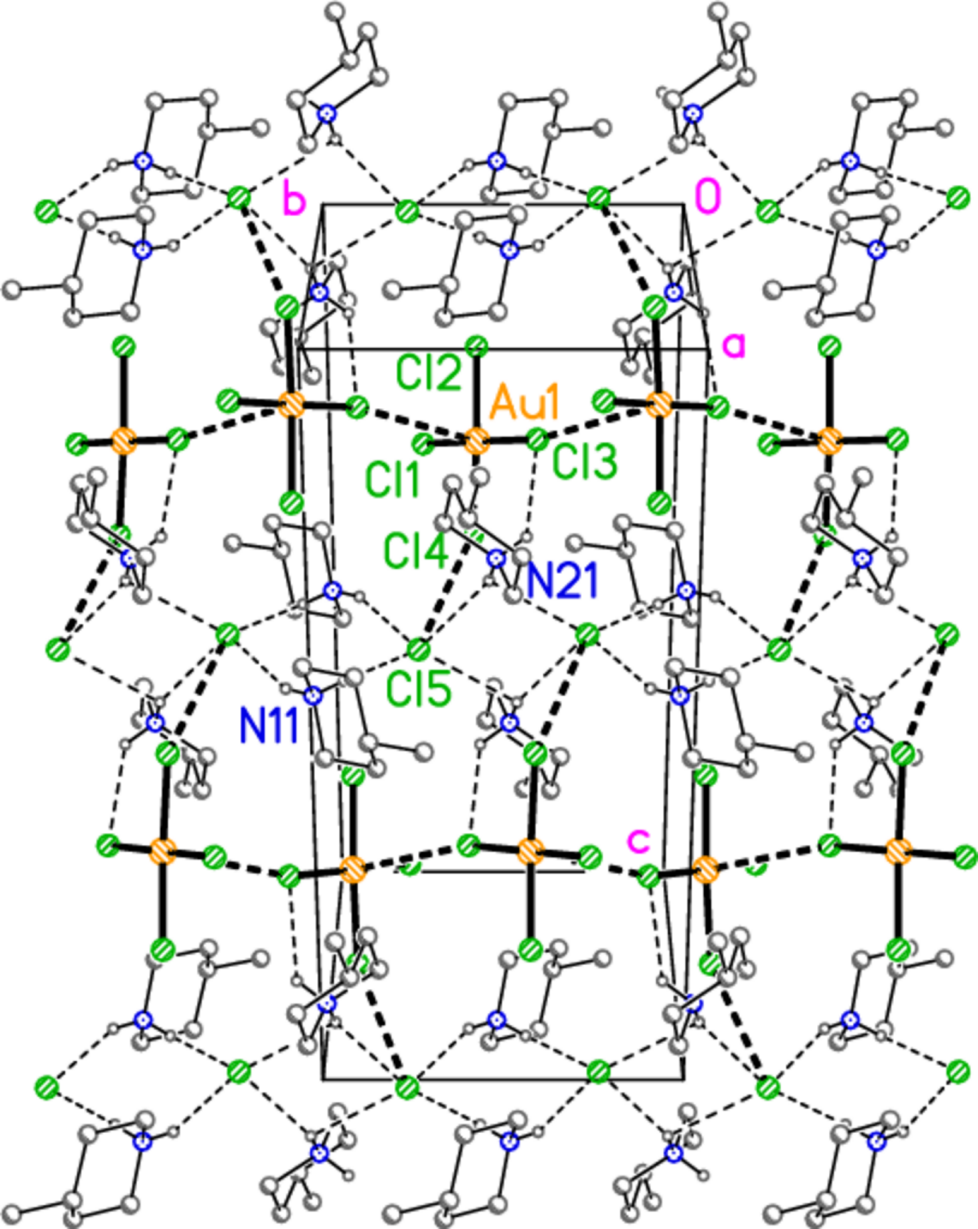
The layer structure of compound **2**, polymorph **2a**, viewed perpendicular to (10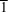). Dashed inter­actions indicate Cl⋯Cl and Au⋯Cl contacts (thick) or hydrogen bonds (thin). The atom Cl4 is partially obscured, and the label N21 is placed some distance to the right of its atom.

In compound **2**, polymorph **2a**, chains of cations and chloride ions run parallel to the *b* axis, but the arrangement differs from that of compound **1** in that the apically linked rings are of two alternating types. One type, involving N11 and its hydrogens, is the same graph set 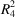
(8) as for **1**, but the other rings only involve one hydrogen H04 at N21, thus forming H_2_Cl_2_ rings of graph set 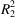
(4) (Fig. 11 ▸). The other hydrogen H03 forms a long hydrogen bond to Cl3 of the tetra­chlorido­aurate ion. A further type of chain, which also runs parallel to the *b* axis, is formed of tetra­chlorido­aurate ions only, with short axial Cl3⋯Au1(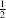 − *x*, −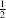 + *y*, 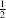 − *z*) contacts of 3.5574 (8) Å and an Au1—Cl3⋯Au1′ angle of 160.69 (3)° (Fig. 12 ▸). Such contacts are well-known for square-planar gold(III) species and have recently been formalized as ‘coinage bonds’ (Daolio *et al.*, 2021 ▸; Pizzi *et al.*, 2022 ▸). The chains are linked by the H03⋯Cl3 hydrogen bond and by a short Cl4⋯Cl5 contact of 3.6319 (11) Å. The two types of chain are linked to form a layer structure parallel to (10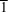) (Fig. 13 ▸, in which the chains run horizontally).

**Figure 14:**
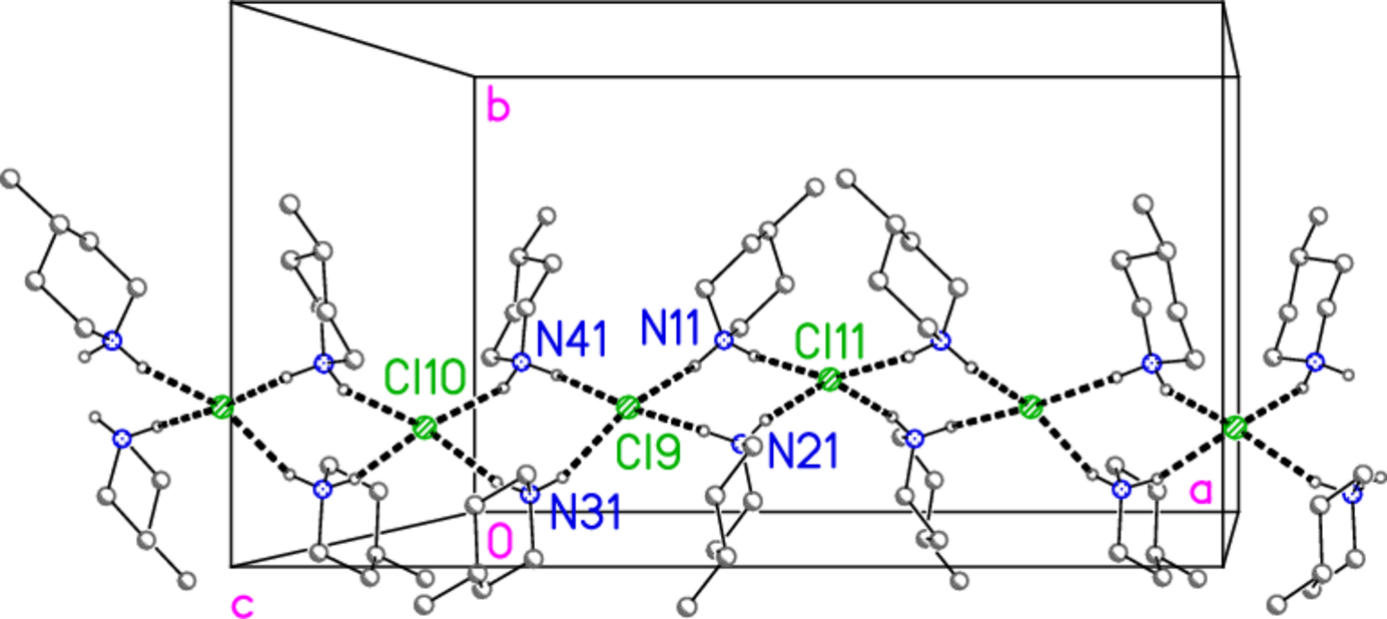
The cation/chloride chain of compound **2**, polymorph **2b**, viewed perpendicular to the *ab* plane. Dashed lines indicate hydrogen bonds. This chain lies at *y*, *z* ≃ 0.25, 0.25; another chain lies at *y*, *z* ≃ 0.75, 0.75.

**Figure 15:**
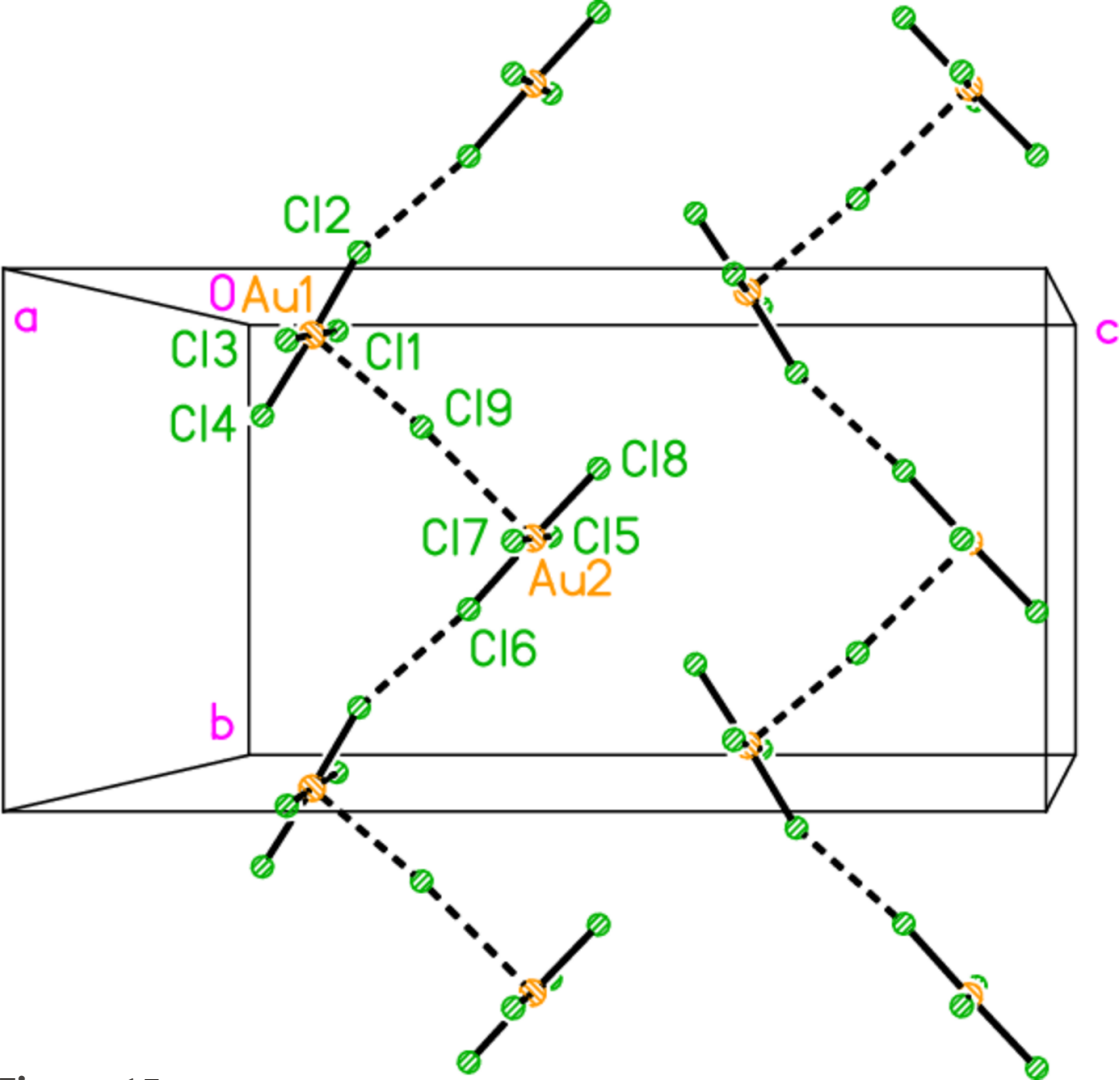
Two tetra­chlorido­aurate(III)/chloride chains of compound **2**, polymorph **2b**, viewed perpendicular to the *bc* plane in the region *x* ≃ 0.25. Dashed lines indicate Au⋯Cl or Cl⋯Cl contacts.

**Figure 16:**
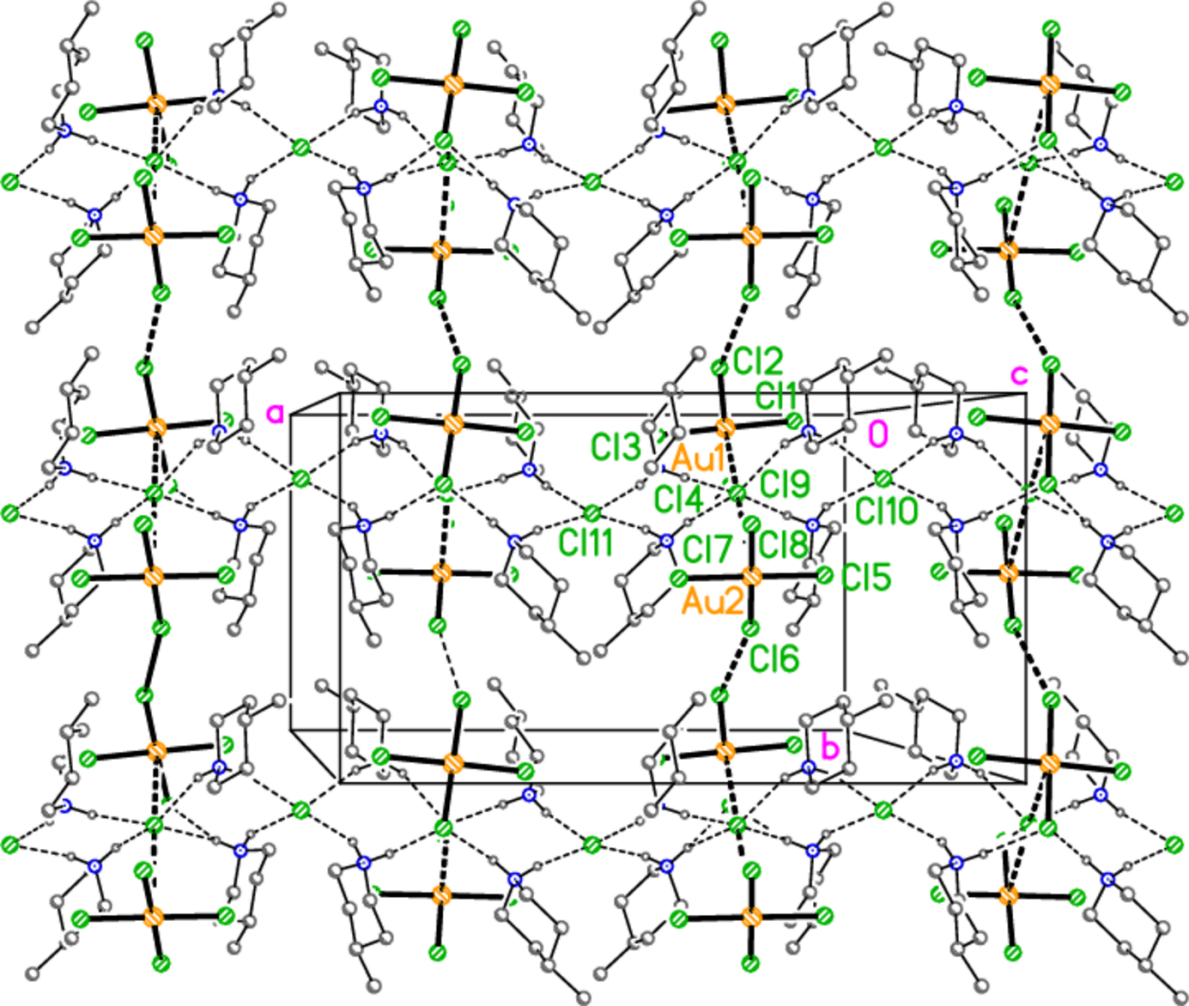
The layer structure of compound **2**, polymorph **2b**, viewed perpendicular to the *ab* plane in the region *z* ≃ 0.25. Dashed inter­actions indicate Cl⋯Cl and Au⋯Cl contacts (thick) or hydrogen bonds (thin).

In polymorph **2b**, the cation/chloride chains again consist solely of apex-linked 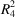
(8) rings, which run parallel to the *a* axis (Fig. 14 ▸). The tetra­chlorido­aurate and chloride anions Cl9 associate to form zigzag chains with overall direction parallel to the *b* axis (Fig. 15 ▸), with short contacts Au1⋯Cl9 = 3.3908 (12), Au2⋯Cl9 = 3.7034 (12) and Cl2⋯Cl6(*x*, −1 + *y*, *z*) = 3.4761 (17) Å. Associated angles are Au1⋯Cl9⋯Au2 = 174.97 (4), Au1—Cl2⋯Cl6′ = 154.66 (5) and Au2—Cl6⋯Cl2(*x*, 1 + *y*, *z*) = 163.48 (5)°, whereby the approximately linear Au⋯Cl^−^⋯Au grouping at the chloride ion Cl9 is striking. The anion chains of the polymorphs **2a**, with propagation *via* axial Au⋯Cl contacts only, and **2b**, with Au⋯Cl^−^⋯Au and Cl⋯Cl contacts, are thus quite different. The two chain types of **2b** combine to form a layer structure parallel to the *ab* plane (Fig. 16 ▸). The inter-chain linkages, in which Cl9 plays a prominent part (it accepts four hydrogen bonds and two coinage bonds), include the three-centre hydrogen bond systems N11—H02⋯(Cl4, Cl9) and N41—H41B⋯(Cl4, Cl9). The second disorder component of the ring at N41, which is not shown in the Figures, forms hydrogen bonds to Cl10 (short) and Cl9 (long).

**Figure 17:**
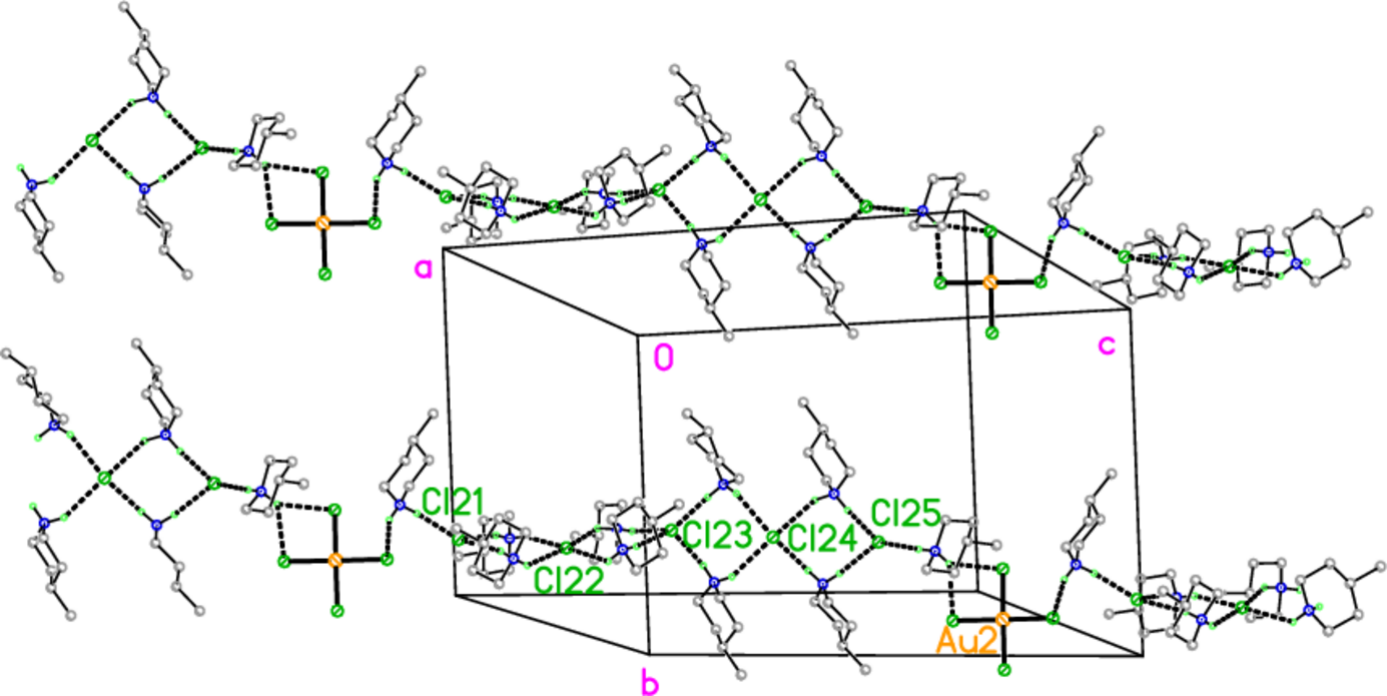
Compound **2**, polymorph **2c**: formation of one-dimensional hydrogen-bonded polymers containing the cations, the chlorides and one tetra­chlorido­aurate (centred on Au2). The inversion-related polymers are omitted for clarity. Dashed inter­actions indicate hydrogen bonds. The view direction is perpendicular to (101).

**Figure 18:**
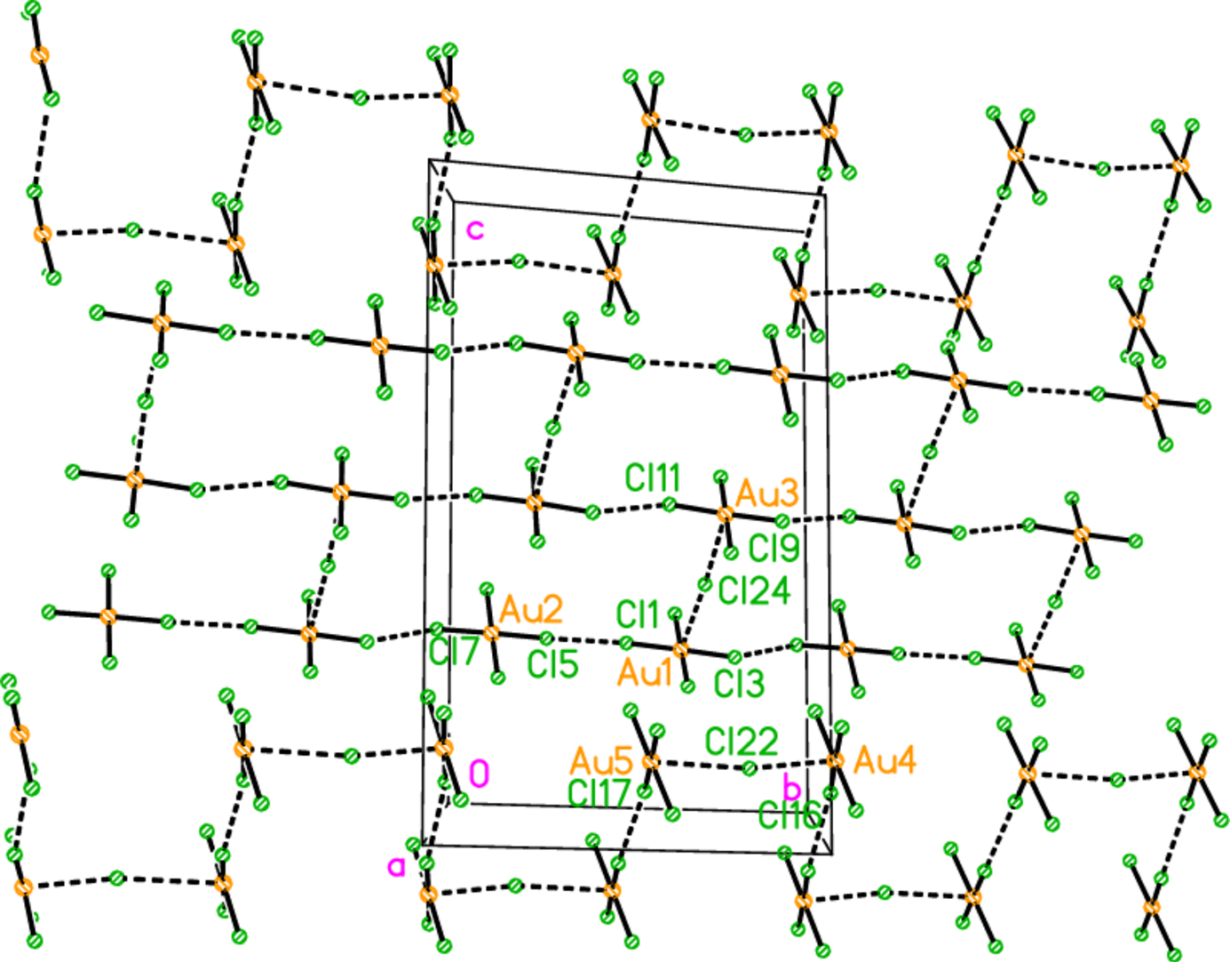
The tetra­chlorido­aurate/chloride substructure of compound **2**, polymorph **2c**, simplified view parallel to the *a* axis. Dashed lines indicate Au⋯Cl or Cl⋯Cl contacts. The atoms Au2, Cl5 and Cl7 are transformed by (−*x*, 1 − *y*, 1 − *z*) from the asymmetric unit.

**Figure 19:**
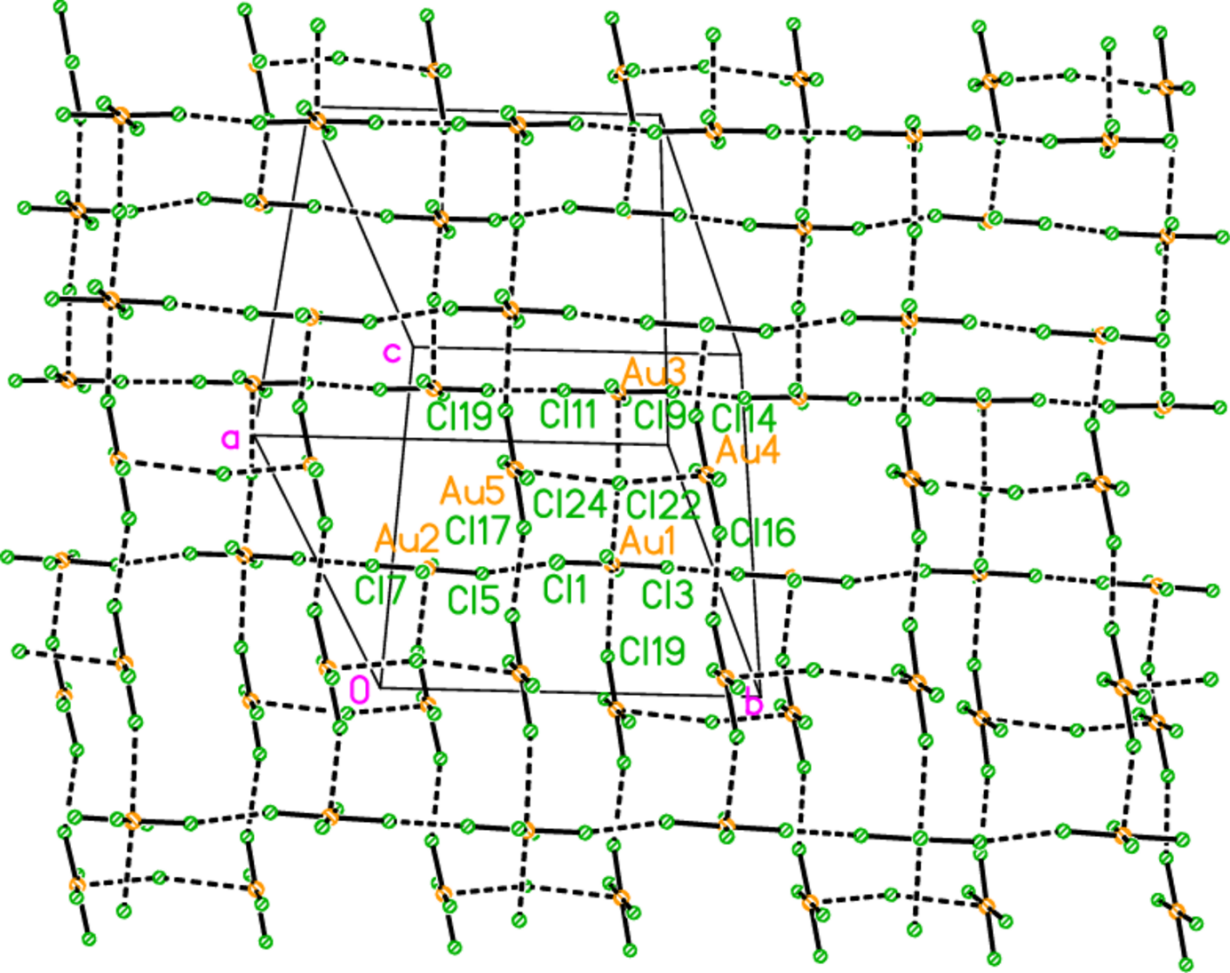
The tetra­chlorido­aurate/chloride substructure of compound **2**, polymorph **2c**, viewed perpendicular to (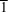 0 2). Dashed lines indicate Au⋯Cl or Cl⋯Cl contacts. In this view direction, the labelled free chlorides Cl22 (bridging Au4 and Au5) and Cl24 (bridging Au1 and Au3) exactly overlap in the centre of the diagram. Further out, towards the edges, they can be distinguished clearly. The atoms Au2, Cl5 and Cl7 are transformed by (−*x*, 1 − *y*, 1 − *z*) from the asymmetric unit.

**Figure 20:**
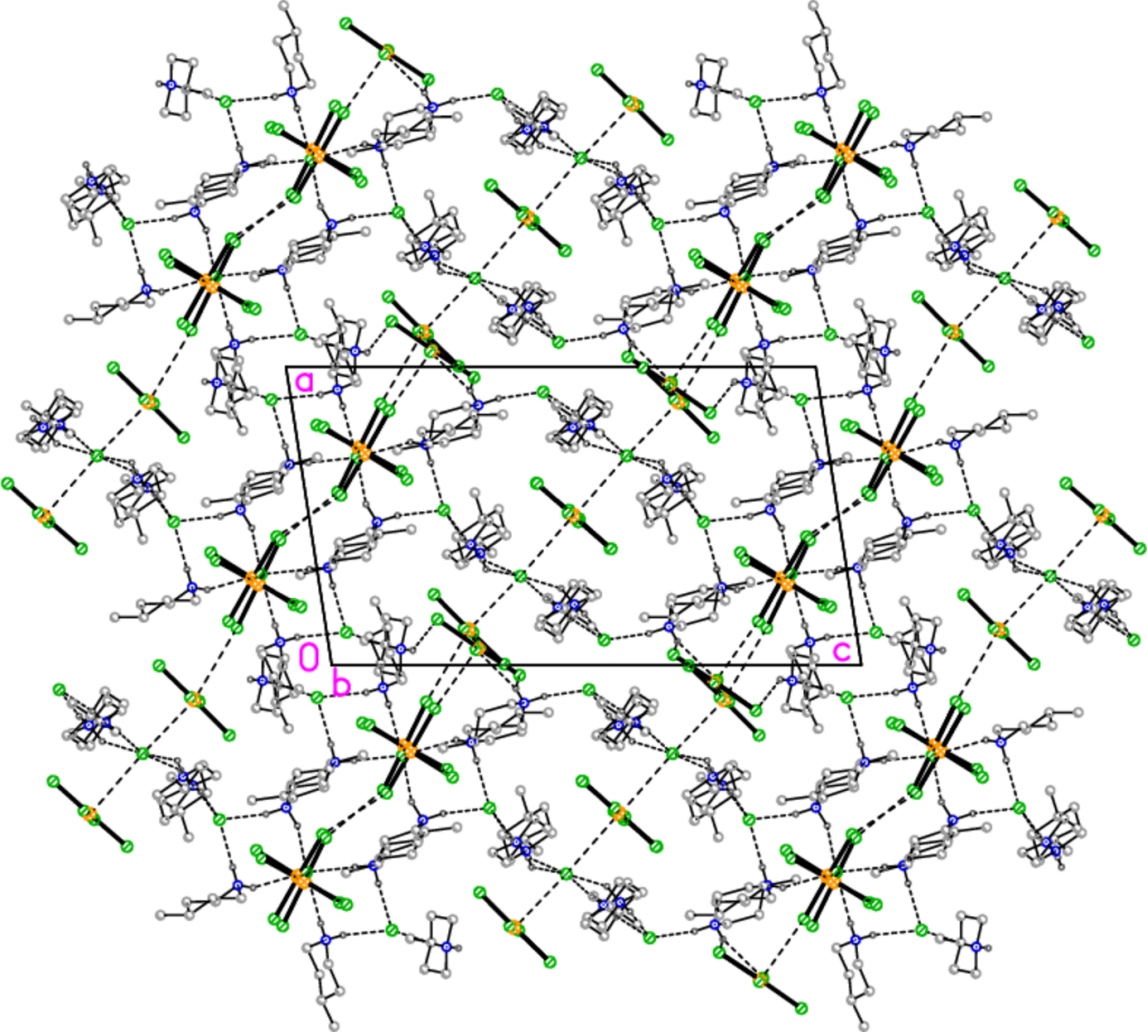
A projection of the entire structure of compound **2**, polymorph **2c**, viewed parallel to the *b* axis. Dashed inter­actions indicate hydrogen bonds, Au⋯Cl and Cl⋯Cl contacts. The tetra­chlorido­aurate substructures run diagonally, top right to bottom left.

The asymmetric unit of polymorph **2c** (Fig. 5 ▸), an ensemble of 20 residues approximately 27 Å long, was chosen to contain a chain of four complete 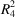
(8) rings, linked at the apices Cl22, Cl23 and Cl24. At the right-hand end of this ensemble, the donor N101—H019 is part of a three-centre hydrogen bond to Cl5 and Cl8, two chlorines of the tetra­chlorido­aurate anion centred on Au2. At the left-hand end, the donor N11—H02 seems at first sight to be unused, but it is linked to Cl6 of the same tetra­chlorido­aurate ion, translated by the operator (1 + *x*, *y*, −1 + *z*). This leads to the formation of a one-dimensional polymer parallel to [10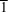] (Fig. 17 ▸). The tetra­chlorido­aurate/chloride substructure is given first as a simplified view (Fig. 18 ▸), in which the inter­actions (Table 11 ▸) are of the type Au⋯Cl^−^ (*via* coinage bonds to the free chlorides) or Cl⋯Cl (between tetra­chlorido­aurate ions). Two separate regions based on Au1–3 and Au4/5 can be recognised, each of which contains an Au⋯Cl^−^⋯Au grouping, both forming one-dimensional arrays parallel to the *b* axis. This view, however, omits the contacts Au1⋯Cl19 and Au2⋯Cl14 between the tetra­chlorido­aurate ions of the two arrays. The view including these contacts (Fig. 19 ▸) is much more complex. It shows the formation of a layer parallel to (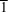
02). The contact lengths have been inter­preted liberally as regards length; one of the former is very long, whereas some of the latter are extremely short. The cation/chloride assemblies of Fig. 17 ▸ inter­sect with the tetra­chlorido­aurate substructure via the hydrogen and coinage bonds at Cl22, the hydrogen bonds H02⋯Cl6′ and H019⋯(Cl5, Cl8) and possibly the borderline contact Cl20⋯Cl21. A projection of the entire structure down the *b* axis (Fig. 20 ▸) shows the tetra­chlorido­aurate/chloride layers edge-on, running diagonally.

**Figure 21:**
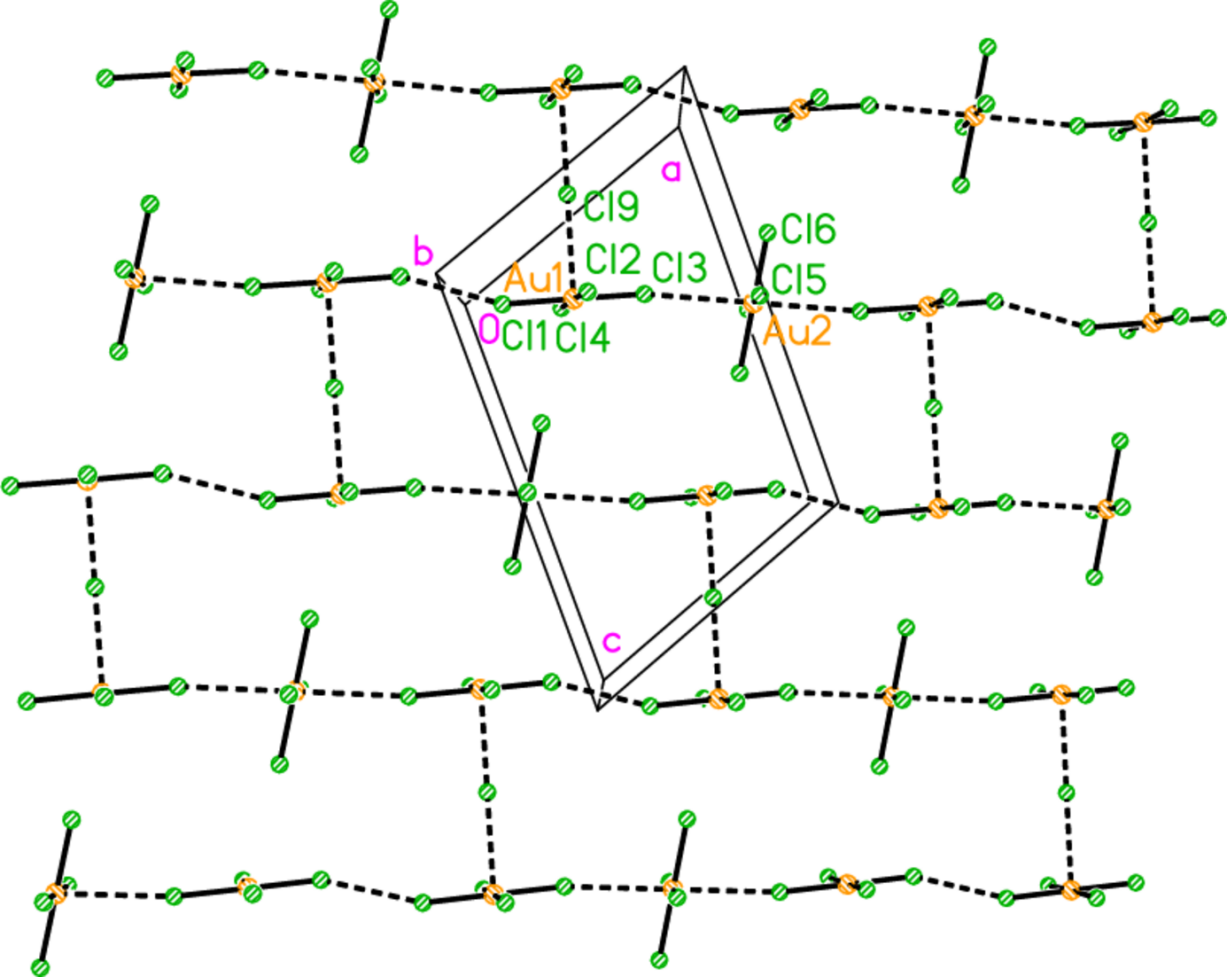
The layer substructure of compound **3**, which involves the two tetra­chlorido­aurate ions at Au1 and Au2 together with the free chloride Cl9, viewed parallel to the *b* axis in the region *y* ≃ 0.5. Dashed inter­actions indicate Cl⋯Cl and Au⋯Cl contacts. Contacts Au1⋯Cl6 (−1 + *x*, *y*, *z*) of 4.0588 (9) Å were considered too long for inclusion.

**Figure 22:**
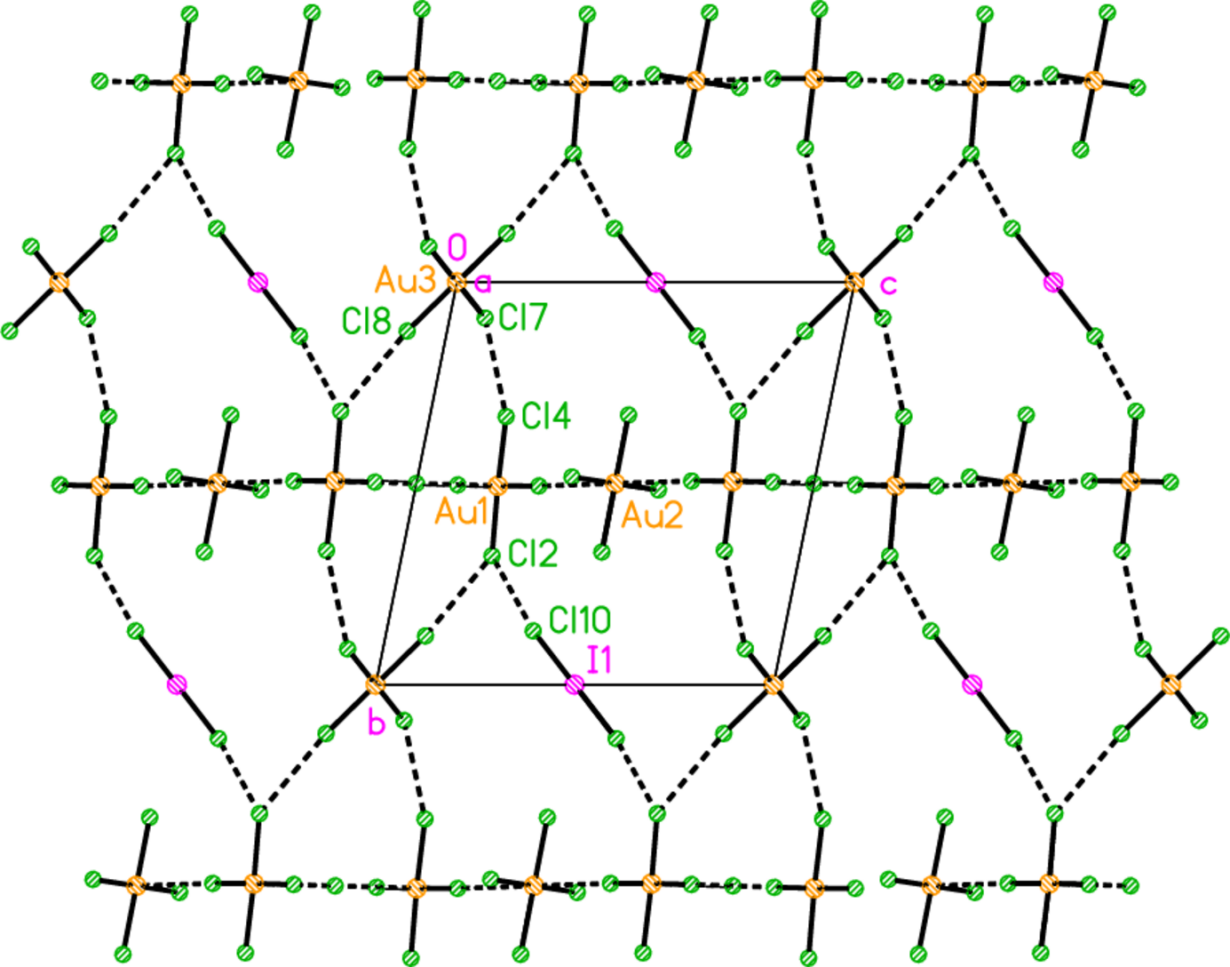
Packing of compound **3**, projected parallel to the *a* axis, showing the linking of the layers of Fig. 19 ▸ by the third tetra­chlorido­aurate and the di­chloro­iodate ions. Dashed inter­actions indicate Cl⋯Cl and Au⋯Cl contacts.

The packing of compound **3** also involves hydrogen bonds and a chloride/tetra­chlorido­aurate substructure. It is more convenient to begin with the latter, for which Au⋯Cl and Cl⋯Cl contacts are listed in Table 12 ▸. The tetra­chlorido­aurate ions centred on Au1 and Au2 associate with the free chloride Cl9 to form a layer structure parallel to the *ac* plane (Fig. 21 ▸), whereby Cl9 again features as part of a linear Au1⋯Cl9⋯Au1′ grouping, cross-linking the chains of tetra­chlorido­aurate ions running parallel to [102]. It is noteworthy that Cl8 participates in two Cl⋯Cl contacts, so that the angles Au1—Cl2⋯Cl(8,10′) are less linear. A projection parallel to the *a* axis (Fig. 22 ▸) shows how the layers are linked via the third tetra­chloro­aurate and the di­chloro­iodate ions.

**Figure 23:**
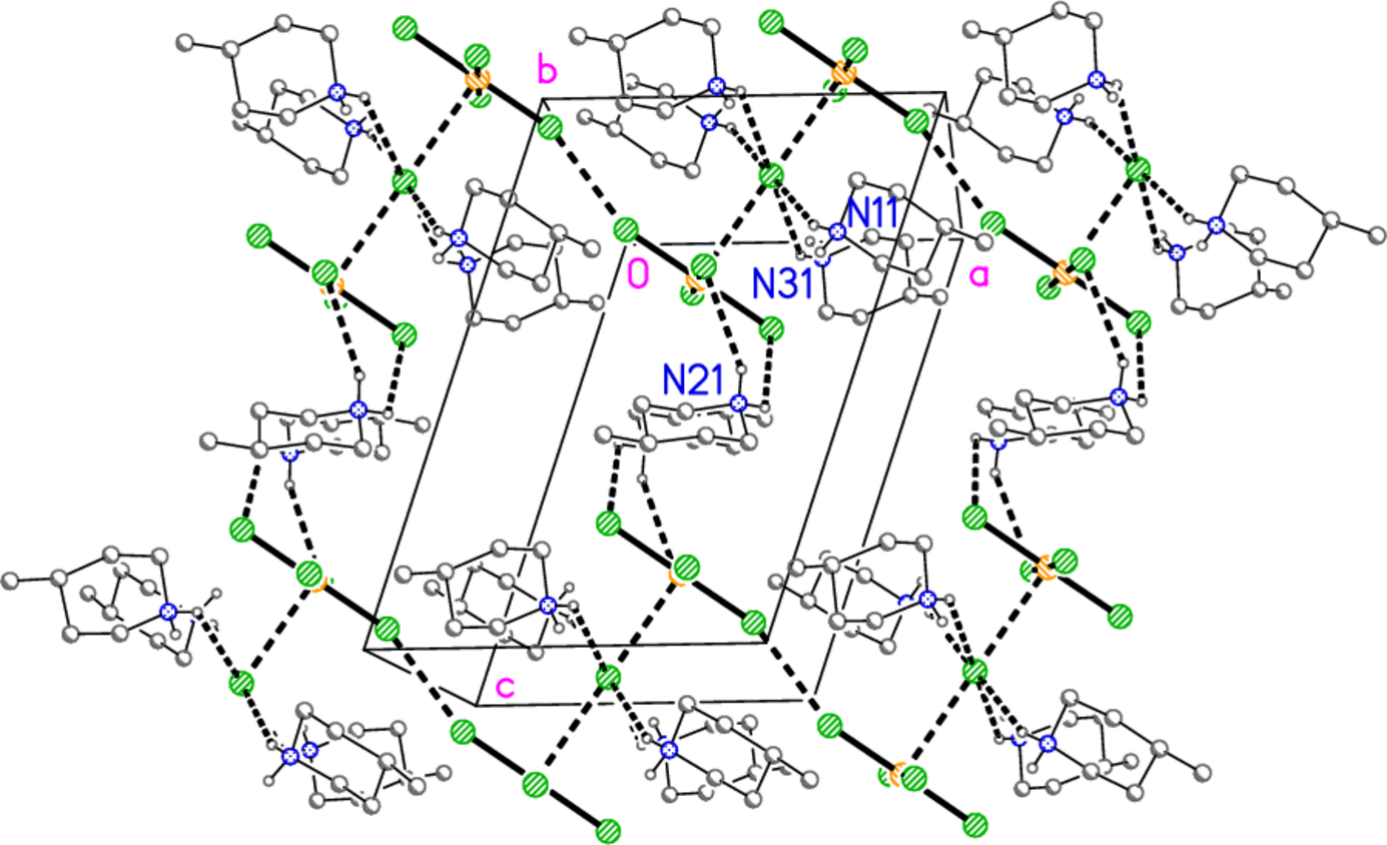
Packing of compound **3**, viewed perpendicular to the *bc* plane. Only the cations, the free chloride Cl9 and the tetra­chlorido­aurate at Au1 are included. Dashed lines indicate hydrogen bonds.

In contrast to the other structures, compound **3** does not form an essentially independent cation/chloride substructure. Instead, the cations may be considered as inter­spersed in the spaces of the anionic substructure, forming hydrogen bonds to chlorine atoms of the anions (Fig. 23 ▸). The hydrogen atoms at N21 are involved in a four-centre and a three-centre hydrogen bonding system, H03⋯(Cl3, Cl5, Cl6′) and H04⋯(Cl2, Cl10); several of the H⋯Cl distances (not only these) are quite long. The free chloride Cl9 accepts four hydrogen bonds and two coinage bonds.

## 4. Database survey

This survey reports on the extent and types of inter­action between the anions of structures involving both halide and tetra­halogenidoaurate(III) ions; these can in principle involve any of the following contact types: Au—*X*⋯*X*—Au; Au—*X*⋯*X*^−^; Au⋯*X*—Au and Au⋯*X*^−^. The search employed the routine ConQuest (Bruno *et al.*, 2002 ▸), part of Version 2024.3.0 of the CSD (Groom *et al.*, 2016 ▸). A search for structures containing an NH^+^ function, an [AuX_4_]^−^ and an X^−^ ion was carried out; it was restricted to non-disordered and error-free structures. Our own previously published structures were excluded, whereafter 24 hits remained. The mere presence of both ion types in a structure is no guarantee of a substructure involving the anions; thus tris­(iso­propyl­ammonium) bis­(tetra­chlorido­aurate(III)) dichloride (refcode DIWYOA; Döring & Jones, 2018 ▸) involves no Cl⋯Cl or Au⋯Cl contacts. One would intuitively expect that the larger the cations, the less chance the anions have to approach each other closely enough to form substructures. Indeed, few of the 24 structures display an anionic framework in more than one dimension. Typical 1D-substructures, axially linked chains of the form ⋯Au⋯Cl^−^⋯Au⋯Cl^−^⋯ with Au⋯Cl = 3.670 or 3.640 Å and linear geometry at the bridging chloride, are seen in 1,2-bis­(4-pyridinium)ethane tetra­chlorido­aurate(III) chloride and the isotypic *trans*-1,2-bis­(4-pyridinium)ethene derivative (CITKIA & CITKOG, Bourne & Moitsheki, 2008 ▸). 4,4′-bipyridinium tetra­chlorido­aurate(III) chloride, with Au⋯Cl 3.683 Å, is similar (NENNIE, Zhang *et al.*, 2006 ▸). In the following, we discuss some of these structures in more detail, giving additional Figures for those structures where the packing was not presented, or in some cases alternative views to those published. At the outset it should be stressed that classical hydrogen bonds, in which the free halide ions often participate, are ignored in this discussion.

**Figure 24:**
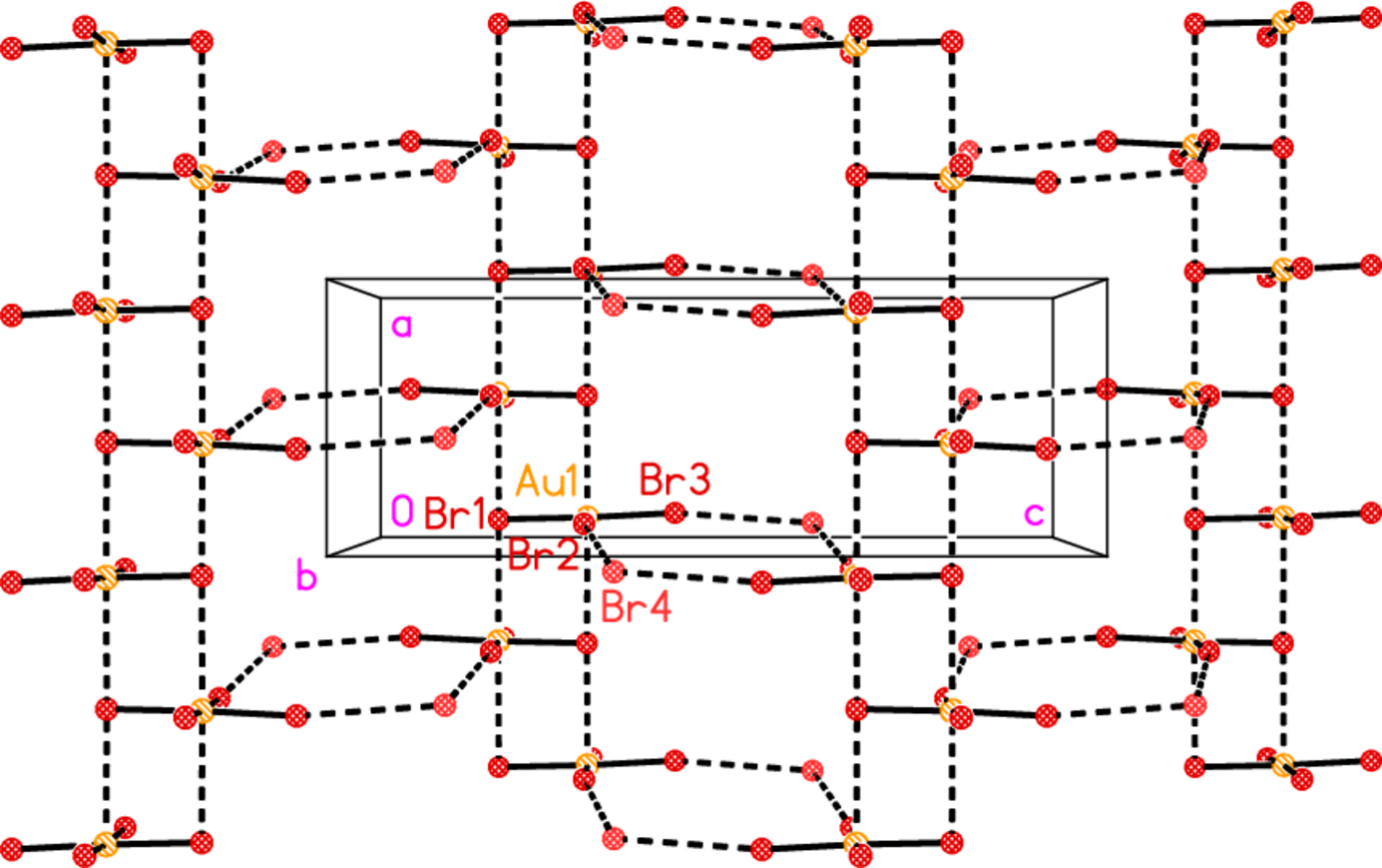
A section of the three-dimensional packing of GEVHAR (Rajeswaran *et al.*, 2007 ▸), drawn from the coordinates stored in the CSD. Dashed lines indicate Br⋯Br contacts. The space group is *Pnma* and the view direction is parallel to the *b* axis. Atoms Au1, Br1 and Br3 lie in the mirror planes at *y* = 0.25; Br4, the free bromide, lies in the mirror plane at *y* = 0.75. Further Br4⋯Br2 and Br4⋯Br3 contacts (3.588 and 3.818 Å respectively) connect the ‘ladder’ substructures, extending the structure in the view direction.

**Figure 25:**
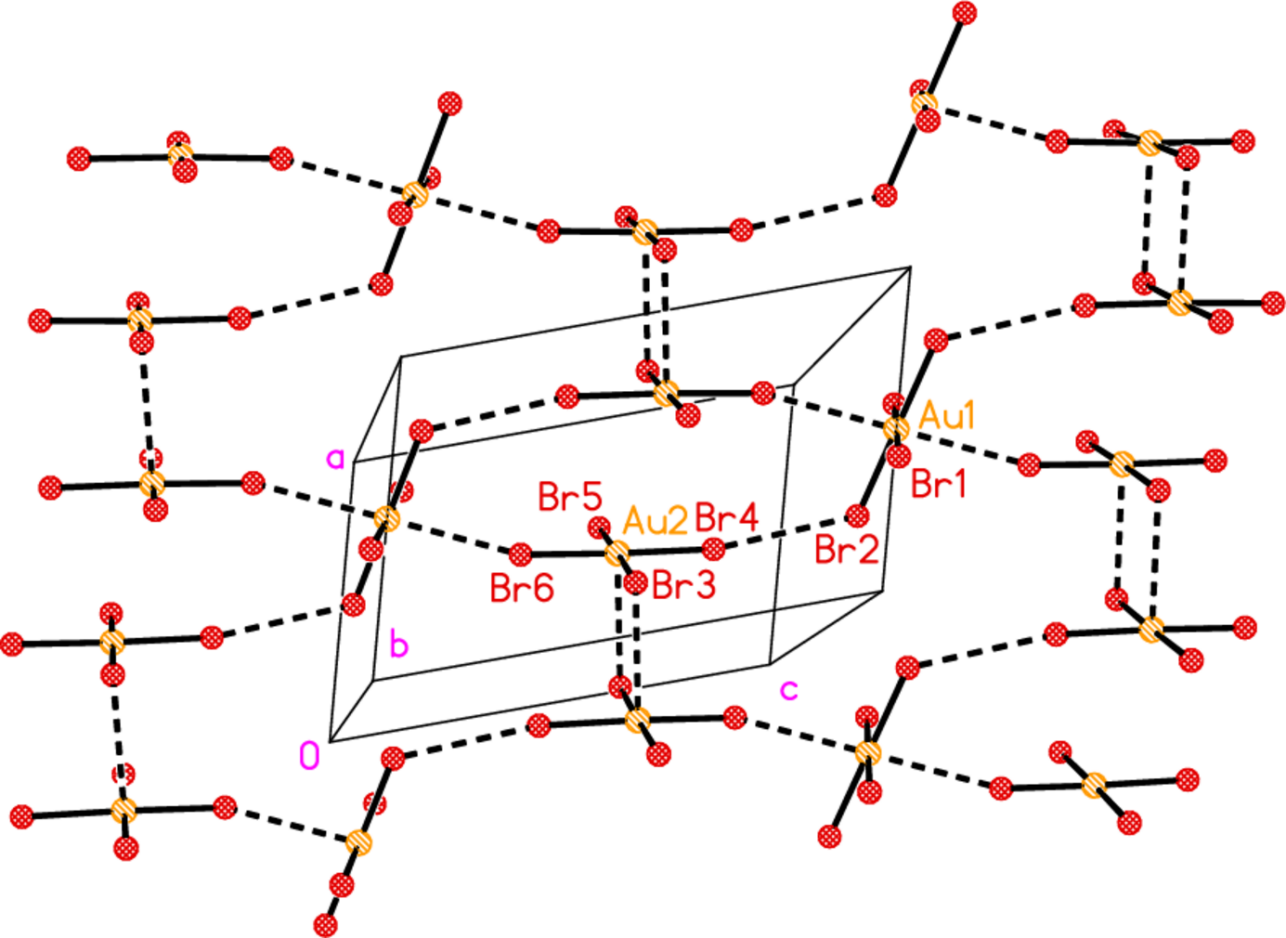
A section of the three-dimensional packing of UYOLAX (Makotchenko *et al.*, 2014 ▸), drawn from the coordinates stored in the CSD. Dashed lines indicate Au⋯Br and Br⋯Br contacts. The space group is *P*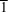 and the view direction is perpendicular to the *ac* plane. Atom Au1 lies on an inversion centre. Contacts Br3⋯Br^−^⋯Br3, not shown here, involve a free bromide on an inversion centre, and link layers in the view direction.

**Figure 26:**
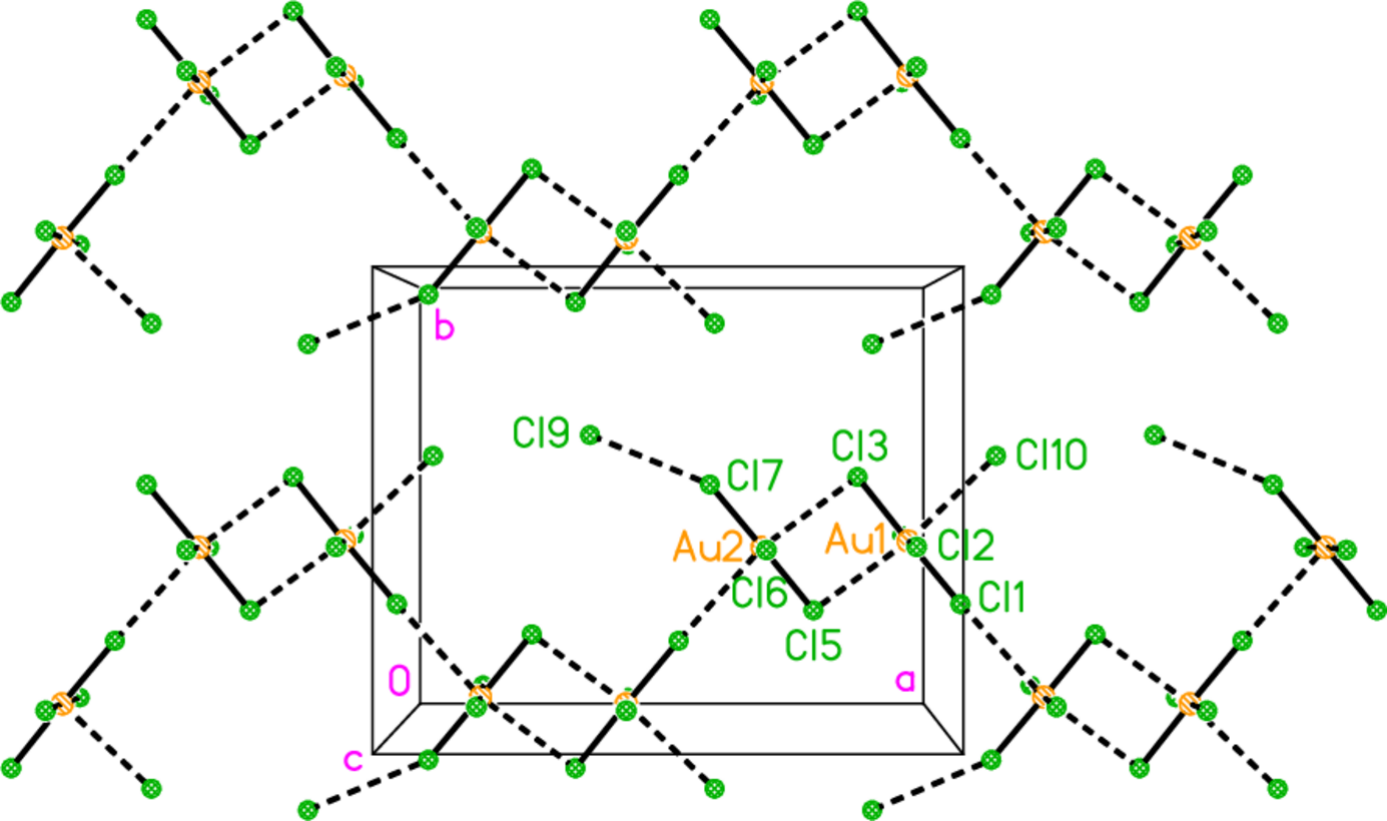
A section of the three-dimensional packing of ZUKTEH (Savchenkov *et al.*, 2020 ▸), drawn from the coordinates stored in the CSD. Atoms Cl4 and Cl8 (both obscured) are not labelled. Dashed lines indicate Au⋯Cl and Cl⋯Cl contacts. The space group is *Pna*2_1_ and the view direction is parallel to the *c* axis in the region *z* ≃ 0.75. Contacts Cl2⋯Cl4, not shown here, link chains in the view direction.

**Figure 27:**
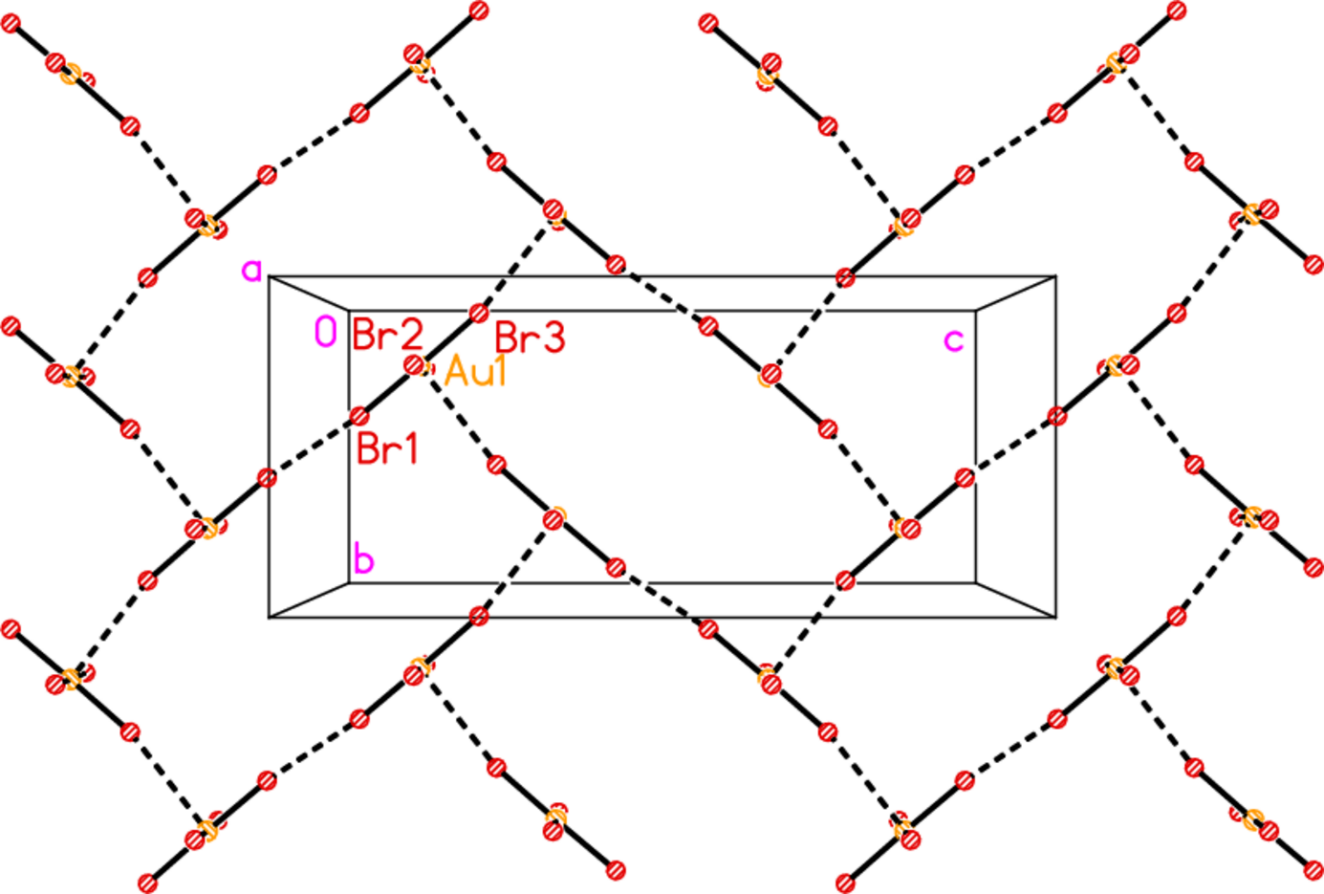
The layer structure of ZUYLEM (Stender *et al.*, 2016 ▸), drawn from the coordinates stored in the CSD. Dashed lines indicate Au⋯Br and Br⋯Br contacts. The space group is *Cmce* (formerly *Cmca*) and the view direction is parallel to the *a* axis in the region *x* ≃ 0.5. The atoms Au1, Br1 and Br3 lie in the mirror plane at *x* = 0.5. Br2 is the atom behind Au1. Contacts Br2⋯Br4 (the free bromide ion on a twofold axis 1/4, *y*, 1/4), not shown here, link layers in the view direction.

In 4,4′-bis­(1*H*-pyrazol-2-ium) tetra­chlorido­aurate(III) chloride (GAZSEH; Domasevitch, 2012 ▸), the tetra­chlorido­aurate ions display the well-known ‘offset stacking’ or ‘ladder’ pattern, whereby one Au—Cl bond of each ion lies anti­parallel to an Au—Cl bond of each stack neighbour, thus enabling two Au⋯Cl coinage bonds to be formed between pairs of ions. This type of substructure has often been reported in neutral trihalogenidogold(III) species such as the four modifications of (tetra­hydro­thio­phene)AuCl_3_ (Upmann *et al.*, 2017 ▸). The same pattern was reported for the tetra­bromido­aurate ions of *p*-phenyl­enedi­ammonium tetra­bromido­aurate(III) bromide (GEVHAR; Rajeswaran *et al.*, 2007 ▸), but a closer inspection shows that the bromide ion also forms Br⋯Br contacts, leading to a three-dimensional packing, a section of which is shown in Fig. 24 ▸. In bis­(ethane-1,2-di­ammonium) tetra­chlorido­aurate(III) trichloride (KIKYOU; Makotchenko *et al.*, 2013 ▸), layers of anions are formed that involve two axial inter­actions in an Au⋯Cl^−^⋯Au grouping (distances of 3.190 and 3.230 Å) and a very short Cl⋯Cl contact of 3.045 Å between tetra­chlorido­aurate ions, leading to an approximately square network. In bis­(di­ethyl­enetri­ammonium) tris­[tetra­bromido­aurate(III)] tribromide (UYOLAX; Makotchenko *et al.*, 2014 ▸), layers consisting solely of tetra­bromido­aurate ions (Fig. 25 ▸) are formed, which contain pairs of offset-stacked ions involving Au1. These are linked in the third dimension by an inversion-symmetric Au—Br⋯Br^−^⋯Br—Au grouping. The second free bromide is attached terminally to the layer, but these contacts are not shown here. The packing was discussed (and contact distances given) in the original paper, but we present it here in a slightly different way. For the structure of 6-amino-7*H*-purine-1,9-diium tetra­choridoaurate(III) chloride hydrate (ZUKTEH; Savchenkov *et al.*, 2020 ▸), the anion substructure was presented without contacts being explicitly drawn, and the contact distances were not complete. Fig. 26 ▸ shows linear chains of residues parallel to the *a* axis in the region *z* ≃ 0.75; further chains occupy the region *z* ≃ 0.25. The layer involves five short contacts: Au1⋯Cl5(−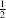 + *x*, 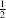 − *y*, *z*) = 3.284, Au1⋯Cl10(1 − *x*, −*y*, 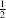 + *z*) =3.438, Au2⋯Cl1 = 3.507, Au2⋯Cl3(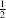 + *x*, 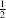 − *y*, *z*) = 3.315, and Cl7⋯Cl9(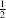 − *x*, 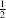 + *y*, −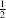 + *z*) = 3.627 Å. The asymmetric unit forms an offset-stacked pair of tetra­chlorido­aurate ions. The layers are joined parallel to the *c* axis by the contact Cl2⋯Cl4(1 − *x*, −*y*, 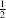 + *z*) 3.635 Å. The free chloride ions Cl9 and Cl10 are terminally linked to the chains (*i.e.* they have no bridging function to other anions, although they play an important role in the hydrogen bonding). The compound bis­(cyclo­hexyl­ammonium) tetra­bromido­aurate(III) bromide is reported in a CSD Communication (ZUYLEM; Stender *et al.*, 2016 ▸). The tetra­bromido­aurate ions assemble *via* the contacts Au1⋯Br3(*x*, 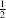 + *y*, 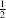 − *z*) = 3.873 and Br1⋯Br1(1 − *x*, 1 − *y*, −*z*) = 3.431 Å to form layers parallel to the *bc* plane at *x* = 0, 0.5, 1, *etc*. (Fig. 27 ▸); layers are linked in the third dimension by the free bromide Br4, with Br2⋯Br4(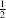 + *x*, 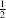 + *y*, *z*) = 3.787 Å.

## 5. Synthesis and crystallization

More details are given in the PhD thesis of CD (Döring, 2016 ▸). Red needles of **1** were obtained from attempts to synthesize (4-Me-pip)AuBr_3_ by the oxidation of [(4-Me-pip)_2_Au][AuBr_2_] with bromine; the solvent system was di­chloro­methane/diisopropyl ether. Similar attempts to obtain (4-Me-pip)AuCl_3_ by the oxidation of [(4-Me-pip)_2_Au][AuCl_2_] with PhICl_2_ in various solvent systems led to (4-Me-pipH)[AuCl_4_] (Döring & Jones, 2016 ▸) and **2a** (yellow plates) as a crystalline mixture from di­chloro­methane/diisopropyl ether; **2c** (irregular orange blocks) from di­chloro­methane/diethyl ether; and **2b** (yellow plates) from aceto­nitrile using a twofold excess of PhICl_2_ (by evaporation). Other solvent systems, in combination with stoichiometric or excess PhICl_2_, led either to **2c** alone or to mixtures of these polymorphs. Finally, **3** (orange plates) was obtained by recrystallizing a sample of ‘(4-Me-pip)AuCl_3_’ from a mixture of nitro­methane and pentane. Clearly a small amount of PhICl_2_ took part at some stage in a reaction other than simple chlorination of the gold(I) species. This was our only observation of this behaviour across a wide range of chlorination reactions.

## 6. Refinement

Details of the measurements and refinements are given in Table 13 ▸.

Structures were refined anisotropically on *F*^2^. Most hydrogen atoms of the NH_2_ groups were refined freely but with N—H distances restrained to be approximately equal (command ‘SADI’; for exceptions, see below). Methyl­ene and methine hydrogens were included at calculated positions and refined using a riding model with C—H = 0.99 or 1.00 Å, respectively. Methyl groups were included as idealized rigid groups with C—H = 0.98 Å and H—C—H = 109.5°, and were allowed to rotate but not tip (command ‘AFIX 137’). *U* values of the hydrogen atoms were fixed at 1.5 × *U*_eq_ of the parent carbon atoms for methyl groups and 1.2 × *U*_eq_ of the parent carbon atoms for other hydrogens. A small number of badly fitting reflections were omitted (**2c**, eight reflections with deviations > 7σ; **3**, three reflections > 7σ).

*Special features and exceptions*: For **2c** and **3**, H⋯H distances across the NH_2_ groups were also restrained with SADI. For **2c**, the hydrogen atoms at N6, N7 and N8 were located in difference maps but could not be refined freely, they were therefore placed at calculated positions (N—H = 0.91 Å) and refined using a riding model. For **2b**, the cation at N4 is disordered over two positions with occupancies 0.538 (7) and 0.462 (7) Å. The two positions were refined isotropically, with hydrogen atoms of the NH_2_ groups included using a riding model (with N—H = 0.91 Å). Appropriate restraints were employed to improve refinement stability, but the dimensions of disordered groups should always be inter­preted with caution.

## Supplementary Material

Crystal structure: contains datablock(s) 1, 2a, 2b, 2c, 3, global. DOI: 10.1107/S2056989025004918/yz2066sup1.cif

Structure factors: contains datablock(s) 1. DOI: 10.1107/S2056989025004918/yz20661sup2.hkl

Structure factors: contains datablock(s) 2a. DOI: 10.1107/S2056989025004918/yz20662asup3.hkl

Structure factors: contains datablock(s) 2b. DOI: 10.1107/S2056989025004918/yz20662bsup4.hkl

Structure factors: contains datablock(s) 2c. DOI: 10.1107/S2056989025004918/yz20662csup5.hkl

Structure factors: contains datablock(s) 3. DOI: 10.1107/S2056989025004918/yz20663sup6.hkl

CCDC references: 2113949, 2113950, 2113951, 2113952, 2113953

Additional supporting information:  crystallographic information; 3D view; checkCIF report

## Figures and Tables

**Table 1:** Selected geometric parameters (Å, °) for **1**[Chem scheme1]

Au1—Br2	2.4259 (4)	Au1—Br1	2.4301 (4)
			
Br2^i^—Au1—Br2	180.0	Br2—Au1—Br1	89.599 (15)
Br2—Au1—Br1^i^	90.400 (15)	Br1^i^—Au1—Br1	180.0
			
C12—C13—C14—C17	−178.7 (4)	C17—C14—C15—C16	177.0 (4)

Symmetry code: (i) 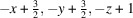.

**Table 2:** Selected geometric parameters (Å, °) for **2a**[Chem scheme1]

Au1—Cl1	2.2752 (8)	Au1—Cl2	2.2872 (8)
Au1—Cl4	2.2802 (7)	Au1—Cl3	2.2879 (8)
			
Cl1—Au1—Cl4	89.91 (3)	Cl4—Au1—Cl3	89.80 (3)
Cl1—Au1—Cl2	89.77 (3)	Cl2—Au1—Cl3	90.72 (3)
Cl4—Au1—Cl2	176.77 (3)	C12—N11—C16	112.8 (3)
Cl1—Au1—Cl3	176.43 (3)		
			
C12—C13—C14—C17	−178.9 (3)	C22—C23—C24—C27	−176.3 (3)
C17—C14—C15—C16	178.1 (3)	C27—C24—C25—C26	177.4 (3)

**Table 3:** Selected geometric parameters (Å, °) for **2b**[Chem scheme1]

Au1—Cl2	2.2701 (11)	Au2—Cl6	2.2751 (11)
Au1—Cl1	2.2856 (11)	Au2—Cl7	2.2792 (11)
Au1—Cl3	2.2879 (11)	Au2—Cl8	2.2832 (11)
Au1—Cl4	2.2904 (11)	Au2—Cl5	2.2842 (11)
			
Cl2—Au1—Cl1	90.12 (4)	Cl6—Au2—Cl7	89.54 (4)
Cl2—Au1—Cl3	90.08 (4)	Cl6—Au2—Cl8	178.35 (4)
Cl1—Au1—Cl3	179.05 (5)	Cl7—Au2—Cl8	89.74 (4)
Cl2—Au1—Cl4	177.98 (5)	Cl6—Au2—Cl5	90.13 (4)
Cl1—Au1—Cl4	89.72 (4)	Cl7—Au2—Cl5	179.55 (5)
Cl3—Au1—Cl4	90.11 (4)	Cl8—Au2—Cl5	90.59 (4)
			
C12—C13—C14—C17	−178.5 (4)	C32—C33—C34—C37	−178.7 (4)
C17—C14—C15—C16	178.8 (4)	C37—C34—C35—C36	180.0 (4)
C22—C23—C24—C27	−178.7 (4)	C42—C43—C44—C47	175.4 (8)
C27—C24—C25—C26	178.8 (4)	C47—C44—C45—C46	−177.9 (9)

**Table 4:** Selected geometric parameters (Å, °) for **2c**[Chem scheme1]

Au1—Cl3	2.2671 (13)	Au3—Cl10	2.2747 (13)
Au1—Cl4	2.2749 (13)	Au3—Cl12	2.2875 (14)
Au1—Cl1	2.2750 (13)	Au4—Cl13	2.2816 (13)
Au1—Cl2	2.2953 (13)	Au4—Cl15	2.2962 (13)
Au2—Cl7	2.2667 (13)	Au4—Cl14	2.2983 (14)
Au2—Cl5	2.2792 (13)	Au4—Cl16	2.2985 (13)
Au2—Cl8	2.2872 (13)	Au5—Cl17	2.2788 (14)
Au2—Cl6	2.2902 (13)	Au5—Cl20	2.2794 (13)
Au3—Cl9	2.2624 (13)	Au5—Cl18	2.2795 (14)
Au3—Cl11	2.2698 (13)	Au5—Cl19	2.2969 (14)
			
Cl3—Au1—Cl4	89.77 (5)	Cl9—Au3—Cl12	90.95 (5)
Cl3—Au1—Cl1	178.58 (6)	Cl11—Au3—Cl12	89.13 (5)
Cl4—Au1—Cl1	89.91 (5)	Cl10—Au3—Cl12	178.86 (6)
Cl3—Au1—Cl2	90.35 (5)	Cl13—Au4—Cl15	179.66 (5)
Cl4—Au1—Cl2	178.72 (5)	Cl13—Au4—Cl14	89.19 (5)
Cl1—Au1—Cl2	89.99 (5)	Cl15—Au4—Cl14	90.76 (5)
Cl7—Au2—Cl5	178.76 (6)	Cl13—Au4—Cl16	89.79 (5)
Cl7—Au2—Cl8	91.29 (5)	Cl15—Au4—Cl16	90.25 (5)
Cl5—Au2—Cl8	89.45 (5)	Cl14—Au4—Cl16	178.41 (5)
Cl7—Au2—Cl6	89.04 (5)	Cl17—Au5—Cl20	90.16 (5)
Cl5—Au2—Cl6	90.24 (5)	Cl17—Au5—Cl18	89.47 (5)
Cl8—Au2—Cl6	178.58 (5)	Cl20—Au5—Cl18	179.45 (6)
Cl9—Au3—Cl11	177.25 (6)	Cl17—Au5—Cl19	179.24 (5)
Cl9—Au3—Cl10	89.01 (5)	Cl20—Au5—Cl19	89.98 (5)
Cl11—Au3—Cl10	90.97 (5)	Cl18—Au5—Cl19	90.40 (5)
			
C12—C13—C14—C17	−179.3 (5)	C62—C63—C64—C67	175.4 (7)
C17—C14—C15—C16	178.2 (5)	C67—C64—C65—C66	−178.5 (6)
C22—C23—C24—C27	180.0 (5)	C72—C73—C74—C77	−179.3 (6)
C27—C24—C25—C26	177.4 (5)	C77—C74—C75—C76	178.6 (5)
C32—C33—C34—C37	179.9 (5)	C82—C83—C84—C87	−178.5 (6)
C37—C34—C35—C36	−179.8 (5)	C87—C84—C85—C86	178.3 (5)
C42—C43—C44—C47	−179.1 (5)	C92—C93—C94—C97	178.1 (5)
C47—C44—C45—C46	179.4 (5)	C97—C94—C95—C96	−177.3 (5)
C52—C53—C54—C57	177.1 (5)	C102—C103—C104—C107	174.2 (5)
C57—C54—C55—C56	−176.5 (5)	C107—C104—C105—C106	−175.4 (5)

**Table 5:** Selected geometric parameters (Å, °) for **3**[Chem scheme1]

Au1—Cl1	2.2733 (8)	Au2—Cl5	2.2794 (8)
Au1—Cl4	2.2792 (9)	Au2—Cl6	2.3052 (8)
Au1—Cl2	2.2882 (9)	Au3—Cl8	2.2837 (9)
Au1—Cl3	2.3003 (8)	I1—Cl10	2.5574 (9)
			
Cl1—Au1—Cl4	89.87 (3)	Cl5—Au2—Cl6^i^	88.49 (3)
Cl1—Au1—Cl2	90.75 (3)	Cl6—Au2—Cl6^i^	180.0
Cl4—Au1—Cl2	177.70 (3)	Cl7^ii^—Au3—Cl7	180.00 (4)
Cl1—Au1—Cl3	179.16 (3)	Cl7—Au3—Cl8^ii^	89.72 (3)
Cl4—Au1—Cl3	89.40 (3)	Cl7—Au3—Cl8	90.28 (3)
Cl2—Au1—Cl3	89.96 (3)	Cl8^ii^—Au3—Cl8	180.0
Cl5—Au2—Cl5^i^	180.0	Cl10—I1—Cl10^iii^	180.0
Cl5—Au2—Cl6	91.51 (3)		
			
C12—C13—C14—C17	−176.0 (3)	C27—C24—C25—C26	178.0 (3)
C17—C14—C15—C16	175.6 (3)	C32—C33—C34—C37	−174.8 (3)
C22—C23—C24—C27	−178.0 (3)	C37—C34—C35—C36	174.3 (3)

Symmetry codes: (i) 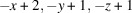; (ii) 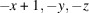; (iii) 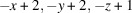.

**Table 6:** Hydrogen-bond geometry (Å, °) for **1**[Chem scheme1]

*D*—H⋯*A*	*D*—H	H⋯*A*	*D*⋯*A*	*D*—H⋯*A*
N11—H02⋯Br3	0.95 (3)	2.36 (3)	3.300 (4)	169 (4)
N11—H01⋯Br3^ii^	0.95 (3)	2.52 (4)	3.281 (4)	137 (4)

Symmetry code: (ii) 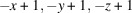.

**Table 7:** Hydrogen-bond geometry (Å, °) for **2a**[Chem scheme1]

*D*—H⋯*A*	*D*—H	H⋯*A*	*D*⋯*A*	*D*—H⋯*A*
N11—H01⋯Cl5	0.89 (2)	2.21 (2)	3.098 (3)	177 (4)
N11—H02⋯Cl5^i^	0.88 (2)	2.32 (3)	3.145 (3)	157 (4)
N21—H03⋯Cl3	0.89 (2)	2.80 (3)	3.453 (3)	131 (3)
N21—H04⋯Cl4	0.89 (2)	2.94 (4)	3.494 (3)	122 (3)
N21—H04⋯Cl5	0.89 (2)	2.43 (3)	3.144 (3)	138 (3)
N21—H04⋯Cl5^ii^	0.89 (2)	2.81 (4)	3.239 (3)	111 (3)

Symmetry codes: (i) 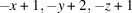; (ii) 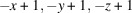.

**Table 8:** Hydrogen-bond geometry (Å, °) for **2b**[Chem scheme1]

*D*—H⋯*A*	*D*—H	H⋯*A*	*D*⋯*A*	*D*—H⋯*A*
N11—H02⋯Cl9	0.92 (2)	2.67 (4)	3.349 (4)	131 (4)
N11—H02⋯Cl4	0.92 (2)	2.68 (4)	3.405 (4)	136 (4)
N11—H01⋯Cl11	0.91 (2)	2.28 (3)	3.177 (4)	169 (6)
N21—H03⋯Cl9	0.91 (2)	2.41 (3)	3.222 (4)	148 (4)
N21—H04⋯Cl11	0.91 (2)	2.24 (3)	3.146 (5)	169 (5)
N31—H05⋯Cl10	0.92 (2)	2.26 (3)	3.147 (4)	163 (5)
N31—H06⋯Cl9	0.91 (2)	2.28 (3)	3.147 (4)	158 (5)
N41—H41*A*⋯Cl10	0.91	2.31	3.158 (7)	155
N41—H41*B*⋯Cl4	0.91	2.68	3.517 (7)	152
N41—H41*B*⋯Cl9	0.91	2.83	3.360 (6)	118
N41′—H41*C*⋯Cl10	0.91	2.20	3.084 (10)	163
N41′—H41*D*⋯Cl9	0.91	2.64	3.430 (9)	146

**Table 9:** Hydrogen-bond geometry (Å, °) for **2c**[Chem scheme1]

*D*—H⋯*A*	*D*—H	H⋯*A*	*D*⋯*A*	*D*—H⋯*A*
N11—H01⋯Cl21	0.90 (2)	2.18 (3)	3.073 (5)	171 (5)
N11—H02⋯Cl6^i^	0.89 (2)	2.60 (5)	3.335 (5)	140 (6)
N21—H03⋯Cl21	0.90 (2)	2.30 (2)	3.182 (5)	167 (4)
N21—H04⋯Cl22	0.90 (2)	2.50 (4)	3.252 (5)	142 (4)
N31—H05⋯Cl21	0.90 (2)	2.22 (3)	3.099 (5)	167 (6)
N31—H06⋯Cl22	0.90 (2)	2.27 (3)	3.134 (5)	162 (7)
N41—H07⋯Cl23	0.89 (2)	2.32 (2)	3.196 (5)	171 (5)
N41—H08⋯Cl22	0.89 (2)	2.59 (6)	3.224 (5)	129 (6)
N51—H09⋯Cl22	0.93 (3)	2.33 (4)	3.180 (5)	151 (6)
N51—H010⋯Cl23	0.93 (3)	2.22 (3)	3.126 (5)	166 (6)
N61—H61*B*⋯Cl23	0.91	2.28	3.161 (6)	164
N61—H61*A*⋯Cl24	0.91	2.57	3.325 (5)	141
N71—H71*A*⋯Cl23	0.91	2.32	3.216 (5)	169
N71—H71*B*⋯Cl24	0.91	2.57	3.254 (5)	132
N81—H81*B*⋯Cl25	0.91	2.26	3.157 (5)	170
N81—H81*A*⋯Cl24	0.91	2.24	3.146 (5)	175
N91—H017⋯Cl24	0.93 (3)	2.56 (6)	3.356 (5)	144 (7)
N91—H018⋯Cl25	0.93 (3)	2.23 (3)	3.134 (5)	163 (7)
N101—H019⋯Cl5	0.93 (3)	2.64 (4)	3.459 (5)	147 (6)
N101—H019⋯Cl8	0.93 (3)	2.72 (5)	3.471 (5)	138 (6)
N101—H020⋯Cl25	0.93 (3)	2.15 (3)	3.075 (5)	170 (4)

Symmetry code: (i) 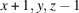.

**Table 10:** Hydrogen-bond geometry (Å, °) for **3**[Chem scheme1]

*D*—H⋯*A*	*D*—H	H⋯*A*	*D*⋯*A*	*D*—H⋯*A*
N11—H01⋯Cl9	0.91 (2)	2.41 (2)	3.270 (3)	157 (3)
N11—H02⋯Cl2	0.92 (2)	2.98 (3)	3.479 (3)	116 (2)
N11—H02⋯Cl10	0.92 (2)	2.58 (2)	3.465 (3)	164 (3)
N21—H03⋯Cl3	0.91 (2)	2.82 (3)	3.317 (3)	116 (3)
N21—H03⋯Cl5	0.91 (2)	2.72 (3)	3.402 (3)	133 (3)
N21—H03⋯Cl6^i^	0.91 (2)	2.79 (2)	3.547 (3)	142 (3)
N21—H04⋯Cl2	0.91 (2)	2.83 (3)	3.566 (3)	139 (3)
N21—H04⋯Cl10	0.91 (2)	2.63 (3)	3.371 (3)	139 (3)
N31—H05⋯Cl3	0.91 (2)	2.92 (3)	3.569 (3)	130 (3)
N31—H05⋯Cl9	0.91 (2)	2.83 (3)	3.538 (3)	136 (3)
N31—H06⋯Cl8	0.91 (2)	2.54 (2)	3.406 (3)	161 (3)

Symmetry code: (i) 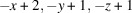.

**Table 11:** Short Au⋯Cl and Cl⋯Cl contacts (Å, °) in the structure of **2c**

Contact	Distance	Operator	Associated angles
Au1⋯Cl19*^*a*^*	3.8488 (14)	−1 + *x*, *y*, *z*	Au1⋯Cl19*^*a*^*—Au5*^*a*^* 167.56 (6)
Au1⋯Cl24	3.4365 (15)		Au1⋯Cl24⋯Au3 174.92 (4)
Au2⋯Cl14*^*a*^*	3.4556 (14)	1 − *x*, 2 − *y*, 1 − *z*	Au2⋯Cl14*^*a*^*—Au4*^*a*^* 161.47 (6)
Au3⋯Cl24	3.7048 (15)		
Au4⋯Cl22	3.3764 (13)		Au4⋯Cl22⋯Au5 167.40 (4)
Au5⋯Cl22	4.0102 (13)		
Cl1⋯Cl5*^*a*^*	3.2111 (18)	−*x*, 1 − *y*, 1 − *z*	Au1—Cl1⋯Cl5^*a*^ 168.60 (7), Cl1⋯Cl5*^*a*^*—Au2*^*a*^* 163.62 (7)
Cl9⋯Cl9*^*a*^*	3.079 (3)	1 − *x*, 2 − *y*, 1 − *z*	Au3—Cl9⋯Cl9*^*a*^* 159.28 (9)
Cl11⋯Cl11*^*a*^*	3.204 (3)	1 − *x*, 1 − *y*, 1 − *z*	Au3—Cl11⋯Cl11*^*a*^* 161.66 (9)
Cl3⋯Cl7*^*a*^*	3.1490 (18)	−*x*, 2 − *y*, 1 − *z*	Au1—Cl3⋯Cl7^*a*^ 154.91 (7), Cl3⋯Cl7*^*a*^*—Au2*^*a*^* 160.34 (7)
Cl16⋯Cl16*^*a*^*	3.516 (3)	1 − *x*, 2 − *y*, −*z*	Au4—Cl16⋯Cl16*^*a*^* 148.13 (8)
Cl17⋯Cl17*^*a*^*	3.469 (3)	1 − *x*, 1 − *y*, −*z*	Au5—Cl17⋯Cl17*^*a*^* 151.45 (8)
Cl20⋯Cl21	3.748 (2)		Au5—Cl20⋯Cl21 142.97 (6)

Note: (*a*) see column 3 for operators.

**Table 12:** Short Au⋯Cl and Cl⋯Cl contacts (Å, °) in the structure of **3**

Contact	Distance	Operator	Associated angles
Au1⋯Cl9	3.2909 (2)		
Au2⋯Cl3	3.6082 (9)		Au2⋯Cl3—Au1 171.71 (4)
Cl1⋯Cl1*^*a*^*	3.3258 (17)	−*x*, 1 − *y*, −*z*	Au1—Cl1⋯Cl1*^*a*^* 160.62 (5)
Cl2⋯Cl8*^*a*^*	3.4819 (13)	1 − *x*, 1 − *y*, −*z*	Au1—Cl2⋯Cl8^*a*^ 144.06 (4), Cl2⋯Cl8*^*a*^*—Au3*^*a*^* 170.85 (4)
Cl2⋯Cl10	3.5880 (13)		Au1—Cl2⋯Cl10 133.20 (4), Cl2⋯Cl10—I1 168.62 (4)
Cl4⋯Cl7	3.4911 (13)		Au1—Cl4⋯Cl7 149.78 (4), Cl4⋯Cl7—Au3 143.72 (4)

Note: (*a*) see column 3 for operators.

**Table 13:** Experimental details

	**1**	**2a**	**2b**	**2c**	**3**
Crystal data					
Chemical formula	(C_6_H_14_N)_2_[AuBr_4_]Br	(C_6_H_14_N)_2_[AuCl_4_]Cl	(C_6_H_14_N)_2_[AuCl_4_]Cl	(C_6_H_14_N)_2_[AuCl_4_]Cl	(C_6_H_14_N)_6_[AuCl_4_]_4_(Cl_2_I)Cl
*M*_r_	796.88	574.58	574.58	574.58	2189.40
Crystal system, space group	Monoclinic, *C*2/*c*	Monoclinic, *P*2_1_/*n*	Monoclinic, *P*2/*c*	Triclinic, *P* 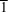	Triclinic, *P* 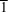
Temperature (K)	100	100	100	100	100
*a*, *b*, *c* (Å)	12.6882 (8), 18.8530 (12), 9.3914 (6)	11.9196 (4), 8.5545 (3), 19.9052 (7)	18.7771 (8), 10.6891 (4), 20.5603 (9)	14.4553 (6), 15.1302 (5), 24.3885 (6)	9.5362 (5), 13.4772 (6), 13.7179 (7)
α, β, γ (°)	90, 102.806 (6), 90	90, 102.955 (4), 90	90, 99.284 (5), 90	90.797 (3), 98.137 (3), 106.407 (4)	98.422 (4), 108.961 (5), 96.954 (4)
*V* (Å^3^)	2190.6 (2)	1977.99 (12)	4072.6 (3)	5057.2 (3)	1622.36 (15)
*Z*	4	4	8	10	1
Radiation type	Mo *K*α	Mo *K*α	Mo *K*α	Mo *K*α	Mo *K*α
μ (mm^−1^)	15.83	8.11	7.87	7.93	10.31
Crystal size (mm)	0.3 × 0.04 × 0.04	0.20 × 0.18 × 0.02	0.15 × 0.10 × 0.03	0.2 × 0.1 × 0.08	0.1 × 0.1 × 0.04

Data collection					
Diffractometer	Oxford Diffraction Xcalibur, Eos	Oxford Diffraction Xcalibur, Eos	Oxford Diffraction Xcalibur, Eos	Oxford Diffraction Xcalibur, Eos	Oxford Diffraction Xcalibur, Eos
Absorption correction	Multi-scan (*CrysAlis PRO*; Rigaku OD, 2013 ▸)	Multi-scan (*CrysAlis PRO*; Rigaku OD, 2013 ▸)	Multi-scan (*CrysAlis PRO*; Rigaku OD, 2013 ▸)	Multi-scan (*CrysAlis PRO*; Rigaku OD, 2013 ▸)	Multi-scan (*CrysAlis PRO*; Rigaku OD, 2013 ▸)
*T*_min_, *T*_max_	0.471, 1.000	0.611, 1.000	0.628, 1.000	0.683, 1.000	0.667, 1.000
No. of measured, independent and observed [*I* > 2σ(*I*)] reflections	30092, 3172, 2592	109437, 5771, 4730	146147, 11802, 8670	316587, 29180, 22450	97738, 9693, 8260
*R*_int_	0.078	0.093	0.115	0.098	0.072
θ values (°)	θ_max_ = 30.0, θ_min_ = 2.2	θ_max_ = 30.0, θ_min_ = 2.2	θ_max_ = 30.0, θ_min_ = 2.2	θ_max_ = 30.0, θ_min_ = 2.3	θ_max_ = 30.9, θ_min_ = 2.3
(sin θ/λ)_max_ (Å^−1^)	0.704	0.704	0.704	0.704	0.722

Refinement					
*R*[*F*^2^ > 2σ(*F*^2^)], *wR*(*F*^2^), *S*	0.032, 0.053, 1.06	0.027, 0.051, 1.05	0.039, 0.065, 1.04	0.041, 0.084, 1.06	0.029, 0.046, 1.05
No. of reflections	3172	5771	11802	29180	9693
No. of parameters	102	196	385	967	331
No. of restraints	1	6	55	64	18
H-atom treatment	H atoms treated by a mixture of independent and constrained refinement	H atoms treated by a mixture of independent and constrained refinement	H atoms treated by a mixture of independent and constrained refinement	H atoms treated by a mixture of independent and constrained refinement	H atoms treated by a mixture of independent and constrained refinement
Δρ_max_, Δρ_min_ (e Å^−3^)	1.27, −0.88	1.36, −1.19	1.47, −1.52	2.67, −1.82	1.16, −0.96

Computer programs: *CrysAlis PRO* (Rigaku OD, 2013 ▸), *SHELXS97* (Sheldrick, 2008 ▸), *SHELXL2019/3* (Sheldrick, 2015 ▸), *XP* (Bruker, 1998 ▸) and *publCIF* (Westrip, 2010 ▸).

## supplementary crystallographic information

### (1) Crystal data

**Table:**

2(C_6_H_14_N)·AuBr_4_·Br	*F*(000) = 1472
*M**_r_* = 796.88	*D*_x_ = 2.416 Mg m^−^^3^
Monoclinic, *C*2/*c*	Mo *K*α radiation, λ = 0.71073 Å
*a* = 12.6882 (8) Å	Cell parameters from 4204 reflections
*b* = 18.8530 (12) Å	θ = 2.7–29.3°
*c* = 9.3914 (6) Å	µ = 15.83 mm^−^^1^
β = 102.806 (6)°	*T* = 100 K
*V* = 2190.6 (2) Å^3^	Needle, red
*Z* = 4	0.3 × 0.04 × 0.04 mm

### (1) Data collection

**Table:**

Oxford Diffraction Xcalibur, Eos diffractometer	3172 independent reflections
Radiation source: Enhance (Mo) X-ray Source	2592 reflections with *I* > 2σ(*I*)
Detector resolution: 16.1419 pixels mm^-1^	*R*_int_ = 0.078
ω scan	θ_max_ = 30.0°, θ_min_ = 2.2°
Absorption correction: multi-scan (CrysAlisPro; Rigaku OD, 2013)	*h* = −17→17
*T*_min_ = 0.471, *T*_max_ = 1.000	*k* = −26→26
30092 measured reflections	*l* = −13→13

### (1) Refinement

**Table:**

Refinement on *F*^2^	Primary atom site location: structure-invariant direct methods
Least-squares matrix: full	Secondary atom site location: difference Fourier map
*R*[*F*^2^ > 2σ(*F*^2^)] = 0.032	Hydrogen site location: mixed
*wR*(*F*^2^) = 0.053	H atoms treated by a mixture of independent and constrained refinement
*S* = 1.06	*w* = 1/[σ^2^(*F*_o_^2^) + (0.0128*P*)^2^ + 4.1505*P*] where *P* = (*F*_o_^2^ + 2*F*_c_^2^)/3
3172 reflections	(Δ/σ)_max_ = 0.001
102 parameters	Δρ_max_ = 1.27 e Å^−^^3^
1 restraint	Δρ_min_ = −0.88 e Å^−^^3^

### (1) Fractional atomic coordinates and isotropic or equivalent isotropic displacement parameters (Å^2^)

**Table:**

	*x*	*y*	*z*	*U*_iso_*/*U*_eq_	
Au1	0.750000	0.750000	0.500000	0.01472 (6)	
Br1	0.64570 (3)	0.64250 (2)	0.43001 (5)	0.02164 (10)	
Br2	0.89211 (3)	0.70305 (2)	0.39160 (5)	0.02257 (11)	
Br3	0.500000	0.48791 (3)	0.250000	0.01525 (12)	
N11	0.6326 (3)	0.4154 (2)	0.5576 (4)	0.0190 (8)	
H01	0.589 (4)	0.418 (3)	0.628 (5)	0.046 (16)*	
H02	0.592 (4)	0.441 (3)	0.477 (4)	0.042 (15)*	
C12	0.6470 (4)	0.3404 (2)	0.5168 (5)	0.0238 (10)	
H12A	0.575564	0.318325	0.478557	0.029*	
H12B	0.684189	0.313433	0.603913	0.029*	
C13	0.7133 (4)	0.3384 (2)	0.4015 (5)	0.0260 (11)	
H13A	0.672914	0.362451	0.312348	0.031*	
H13B	0.724353	0.288375	0.376137	0.031*	
C14	0.8225 (4)	0.3739 (3)	0.4522 (5)	0.0253 (11)	
H14	0.864305	0.347337	0.538553	0.030*	
C15	0.8057 (4)	0.4499 (3)	0.4997 (5)	0.0247 (10)	
H15A	0.876943	0.471726	0.540513	0.030*	
H15B	0.770085	0.477910	0.413254	0.030*	
C16	0.7371 (4)	0.4529 (2)	0.6135 (5)	0.0225 (10)	
H16A	0.776354	0.430193	0.705026	0.027*	
H16B	0.723022	0.502950	0.635220	0.027*	
C17	0.8863 (4)	0.3725 (3)	0.3325 (5)	0.0406 (14)	
H17A	0.844929	0.396712	0.245474	0.061*	
H17B	0.899308	0.323236	0.308113	0.061*	
H17C	0.955581	0.396763	0.366854	0.061*	

### (1) Atomic displacement parameters (Å^2^)

**Table:**

	*U*^11^	*U*^22^	*U*^33^	*U*^12^	*U*^13^	*U*^23^
Au1	0.01436 (11)	0.01629 (12)	0.01283 (11)	−0.00014 (9)	0.00160 (8)	−0.00163 (9)
Br1	0.0225 (2)	0.0208 (2)	0.0227 (2)	−0.00606 (18)	0.00738 (18)	−0.00630 (18)
Br2	0.0175 (2)	0.0269 (3)	0.0239 (2)	0.00131 (18)	0.00587 (18)	−0.00617 (19)
Br3	0.0161 (3)	0.0163 (3)	0.0140 (3)	0.000	0.0048 (2)	0.000
N11	0.020 (2)	0.021 (2)	0.0169 (19)	0.0065 (16)	0.0038 (16)	0.0010 (16)
C12	0.021 (2)	0.018 (2)	0.030 (3)	0.0035 (18)	0.003 (2)	−0.004 (2)
C13	0.033 (3)	0.027 (3)	0.017 (2)	0.018 (2)	0.005 (2)	−0.001 (2)
C14	0.023 (2)	0.041 (3)	0.014 (2)	0.015 (2)	0.0089 (19)	0.007 (2)
C15	0.022 (2)	0.033 (3)	0.019 (2)	−0.002 (2)	0.0044 (19)	0.002 (2)
C16	0.027 (3)	0.022 (2)	0.018 (2)	−0.0019 (19)	0.0054 (19)	−0.0033 (19)
C17	0.032 (3)	0.069 (4)	0.025 (3)	0.019 (3)	0.015 (2)	0.007 (3)

### (1) Geometric parameters (Å, º)

**Table:**

Au1—Br2^i^	2.4259 (4)	C13—H13B	0.9900
Au1—Br2	2.4259 (4)	C14—C17	1.524 (6)
Au1—Br1^i^	2.4301 (4)	C14—C15	1.529 (6)
Au1—Br1	2.4301 (4)	C14—H14	1.0000
N11—C12	1.486 (5)	C15—C16	1.522 (5)
N11—C16	1.491 (6)	C15—H15A	0.9900
N11—H01	0.95 (3)	C15—H15B	0.9900
N11—H02	0.95 (3)	C16—H16A	0.9900
C12—C13	1.512 (6)	C16—H16B	0.9900
C12—H12A	0.9900	C17—H17A	0.9800
C12—H12B	0.9900	C17—H17B	0.9800
C13—C14	1.517 (6)	C17—H17C	0.9800
C13—H13A	0.9900		
			
Br2^i^—Au1—Br2	180.0	C13—C14—C17	111.0 (4)
Br2^i^—Au1—Br1^i^	89.602 (15)	C13—C14—C15	109.2 (4)
Br2—Au1—Br1^i^	90.400 (15)	C17—C14—C15	111.3 (4)
Br2^i^—Au1—Br1	90.399 (15)	C13—C14—H14	108.4
Br2—Au1—Br1	89.599 (15)	C17—C14—H14	108.4
Br1^i^—Au1—Br1	180.0	C15—C14—H14	108.4
C12—N11—C16	113.0 (3)	C16—C15—C14	112.2 (4)
C12—N11—H01	110 (3)	C16—C15—H15A	109.2
C16—N11—H01	110 (3)	C14—C15—H15A	109.2
C12—N11—H02	111 (3)	C16—C15—H15B	109.2
C16—N11—H02	109 (3)	C14—C15—H15B	109.2
H01—N11—H02	104 (4)	H15A—C15—H15B	107.9
N11—C12—C13	109.2 (4)	N11—C16—C15	109.7 (3)
N11—C12—H12A	109.8	N11—C16—H16A	109.7
C13—C12—H12A	109.8	C15—C16—H16A	109.7
N11—C12—H12B	109.8	N11—C16—H16B	109.7
C13—C12—H12B	109.8	C15—C16—H16B	109.7
H12A—C12—H12B	108.3	H16A—C16—H16B	108.2
C12—C13—C14	112.2 (4)	C14—C17—H17A	109.5
C12—C13—H13A	109.2	C14—C17—H17B	109.5
C14—C13—H13A	109.2	H17A—C17—H17B	109.5
C12—C13—H13B	109.2	C14—C17—H17C	109.5
C14—C13—H13B	109.2	H17A—C17—H17C	109.5
H13A—C13—H13B	107.9	H17B—C17—H17C	109.5
			
C16—N11—C12—C13	−58.3 (5)	C13—C14—C15—C16	54.1 (5)
N11—C12—C13—C14	57.5 (5)	C17—C14—C15—C16	177.0 (4)
C12—C13—C14—C17	−178.7 (4)	C12—N11—C16—C15	57.1 (5)
C12—C13—C14—C15	−55.6 (5)	C14—C15—C16—N11	−54.7 (5)

Symmetry code: (i) −*x*+3/2, −*y*+3/2, −*z*+1.

### (1) Hydrogen-bond geometry (Å, º)

**Table:**

*D*—H···*A*	*D*—H	H···*A*	*D*···*A*	*D*—H···*A*
N11—H02···Br3	0.95 (3)	2.36 (3)	3.300 (4)	169 (4)
N11—H01···Br3^ii^	0.95 (3)	2.52 (4)	3.281 (4)	137 (4)

Symmetry code: (ii) −*x*+1, −*y*+1, −*z*+1.

### Bis(4-methylpiperidinium) tetrabromidoaurate(III) bromide (2a) Crystal data

**Table:**

(C_6_H_14_N)_2_[AuCl_4_]Cl	*F*(000) = 1112
*M**_r_* = 574.58	*D*_x_ = 1.929 Mg m^−^^3^
Monoclinic, *P*2_1_/*n*	Mo *K*α radiation, λ = 0.71073 Å
*a* = 11.9196 (4) Å	Cell parameters from 14350 reflections
*b* = 8.5545 (3) Å	θ = 2.6–29.0°
*c* = 19.9052 (7) Å	µ = 8.11 mm^−^^1^
β = 102.955 (4)°	*T* = 100 K
*V* = 1977.99 (12) Å^3^	Plate, yellow
*Z* = 4	0.20 × 0.18 × 0.02 mm

### Bis(4-methylpiperidinium) tetrabromidoaurate(III) bromide (2a) Data collection

**Table:**

Oxford Diffraction Xcalibur, Eos diffractometer	5771 independent reflections
Radiation source: Enhance (Mo) X-ray Source	4730 reflections with *I* > 2σ(*I*)
Detector resolution: 16.1419 pixels mm^-1^	*R*_int_ = 0.093
ω scan	θ_max_ = 30.0°, θ_min_ = 2.2°
Absorption correction: multi-scan (CrysAlisPro; Rigaku OD, 2013)	*h* = −16→16
*T*_min_ = 0.611, *T*_max_ = 1.000	*k* = −11→12
109437 measured reflections	*l* = −27→28

### Bis(4-methylpiperidinium) tetrabromidoaurate(III) bromide (2a) Refinement

**Table:**

Refinement on *F*^2^	Primary atom site location: structure-invariant direct methods
Least-squares matrix: full	Secondary atom site location: difference Fourier map
*R*[*F*^2^ > 2σ(*F*^2^)] = 0.027	Hydrogen site location: mixed
*wR*(*F*^2^) = 0.051	H atoms treated by a mixture of independent and constrained refinement
*S* = 1.05	*w* = 1/[σ^2^(*F*_o_^2^) + (0.0188*P*)^2^ + 0.4543*P*] where *P* = (*F*_o_^2^ + 2*F*_c_^2^)/3
5771 reflections	(Δ/σ)_max_ = 0.002
196 parameters	Δρ_max_ = 1.36 e Å^−^^3^
6 restraints	Δρ_min_ = −1.19 e Å^−^^3^

### Bis(4-methylpiperidinium) tetrabromidoaurate(III) bromide (2a) Fractional atomic coordinates and isotropic or equivalent isotropic displacement parameters (Å^2^)

**Table:**

	*x*	*y*	*z*	*U*_iso_*/*U*_eq_	
Au1	0.12963 (2)	0.57480 (2)	0.30094 (2)	0.01413 (4)	
Cl1	−0.00345 (7)	0.73055 (10)	0.33360 (4)	0.02595 (19)	
Cl2	0.02152 (7)	0.57500 (10)	0.19040 (4)	0.02372 (18)	
Cl3	0.25817 (7)	0.40513 (9)	0.27059 (4)	0.02354 (18)	
Cl4	0.24188 (7)	0.58758 (9)	0.40983 (4)	0.02000 (17)	
Cl5	0.51690 (7)	0.73443 (9)	0.50633 (4)	0.02119 (17)	
N11	0.6828 (3)	1.0179 (3)	0.52815 (17)	0.0249 (7)	
H01	0.635 (3)	0.937 (3)	0.520 (2)	0.040 (6)*	
H02	0.636 (3)	1.096 (3)	0.531 (2)	0.040 (6)*	
C12	0.7612 (3)	0.9856 (4)	0.59581 (18)	0.0252 (8)	
H12A	0.715745	0.969530	0.631218	0.030*	
H12B	0.813085	1.075855	0.609923	0.030*	
C13	0.8313 (3)	0.8411 (4)	0.59031 (17)	0.0222 (8)	
H13A	0.779165	0.749955	0.579664	0.027*	
H13B	0.884969	0.821518	0.635221	0.027*	
C14	0.8999 (3)	0.8573 (4)	0.53461 (18)	0.0218 (7)	
H14	0.955107	0.946081	0.547316	0.026*	
C15	0.8171 (3)	0.8967 (4)	0.46645 (17)	0.0234 (8)	
H15A	0.861708	0.914951	0.430811	0.028*	
H15B	0.765487	0.806449	0.451607	0.028*	
C16	0.7453 (3)	1.0391 (4)	0.47174 (19)	0.0265 (8)	
H16A	0.795594	1.132348	0.481286	0.032*	
H16B	0.689271	1.056020	0.427467	0.032*	
C17	0.9689 (3)	0.7088 (4)	0.5285 (2)	0.0383 (10)	
H17A	0.915964	0.620502	0.515775	0.057*	
H17B	1.021796	0.687146	0.572749	0.057*	
H17C	1.012957	0.723801	0.492892	0.057*	
N21	0.5144 (3)	0.5186 (3)	0.37818 (15)	0.0209 (6)	
H03	0.478 (3)	0.436 (3)	0.3564 (19)	0.040 (6)*	
H04	0.488 (3)	0.539 (4)	0.4156 (15)	0.040 (6)*	
C22	0.6383 (3)	0.4730 (4)	0.39781 (19)	0.0249 (8)	
H22A	0.646442	0.374722	0.424704	0.030*	
H22B	0.682588	0.555456	0.427249	0.030*	
C23	0.6862 (3)	0.4506 (4)	0.3344 (2)	0.0286 (8)	
H23A	0.648522	0.358872	0.308443	0.034*	
H23B	0.769593	0.427975	0.348859	0.034*	
C24	0.6685 (3)	0.5933 (4)	0.28683 (18)	0.0251 (8)	
H24	0.713218	0.682951	0.311747	0.030*	
C25	0.5407 (3)	0.6361 (4)	0.26959 (17)	0.0239 (8)	
H25A	0.529406	0.732463	0.241350	0.029*	
H25B	0.496473	0.551134	0.241838	0.029*	
C26	0.4947 (3)	0.6617 (4)	0.33334 (18)	0.0234 (8)	
H26A	0.533984	0.752402	0.359341	0.028*	
H26B	0.411326	0.684907	0.320002	0.028*	
C27	0.7111 (4)	0.5607 (5)	0.2217 (2)	0.0366 (10)	
H27A	0.671625	0.468478	0.198415	0.055*	
H27B	0.794261	0.541498	0.233775	0.055*	
H27C	0.694875	0.651169	0.190796	0.055*	

### Bis(4-methylpiperidinium) tetrabromidoaurate(III) bromide (2a) Atomic displacement parameters (Å^2^)

**Table:**

	*U*^11^	*U*^22^	*U*^33^	*U*^12^	*U*^13^	*U*^23^
Au1	0.01380 (6)	0.01309 (6)	0.01494 (6)	−0.00028 (5)	0.00206 (4)	−0.00053 (5)
Cl1	0.0203 (4)	0.0283 (5)	0.0282 (5)	0.0069 (3)	0.0032 (3)	−0.0063 (4)
Cl2	0.0197 (4)	0.0313 (5)	0.0175 (4)	0.0013 (4)	−0.0014 (3)	−0.0023 (4)
Cl3	0.0216 (4)	0.0251 (4)	0.0231 (4)	0.0062 (3)	0.0033 (3)	−0.0067 (3)
Cl4	0.0217 (4)	0.0194 (4)	0.0168 (4)	0.0027 (3)	−0.0002 (3)	−0.0010 (3)
Cl5	0.0214 (4)	0.0167 (4)	0.0232 (4)	−0.0015 (3)	0.0003 (3)	0.0011 (3)
N11	0.0186 (15)	0.0162 (15)	0.0405 (19)	0.0002 (12)	0.0081 (14)	−0.0022 (14)
C12	0.031 (2)	0.0240 (19)	0.0215 (18)	−0.0058 (16)	0.0079 (15)	−0.0050 (15)
C13	0.0225 (18)	0.0207 (18)	0.0210 (18)	−0.0025 (14)	0.0002 (14)	0.0023 (14)
C14	0.0196 (17)	0.0211 (18)	0.0248 (19)	−0.0018 (14)	0.0052 (14)	0.0011 (14)
C15	0.0224 (17)	0.0277 (19)	0.0212 (18)	−0.0040 (14)	0.0075 (14)	−0.0023 (14)
C16	0.0248 (18)	0.0255 (19)	0.0263 (19)	−0.0026 (15)	−0.0005 (15)	0.0079 (15)
C17	0.027 (2)	0.032 (2)	0.059 (3)	0.0072 (17)	0.018 (2)	0.008 (2)
N21	0.0192 (15)	0.0213 (15)	0.0218 (16)	0.0022 (12)	0.0036 (12)	0.0054 (13)
C22	0.0206 (18)	0.0210 (18)	0.030 (2)	0.0042 (14)	−0.0018 (15)	0.0052 (15)
C23	0.0227 (18)	0.023 (2)	0.038 (2)	0.0017 (15)	0.0033 (16)	−0.0015 (16)
C24	0.0247 (18)	0.0242 (19)	0.0277 (19)	−0.0079 (15)	0.0089 (15)	−0.0040 (15)
C25	0.0273 (19)	0.0241 (18)	0.0191 (17)	−0.0017 (15)	0.0023 (15)	0.0018 (14)
C26	0.0215 (17)	0.0194 (18)	0.0278 (19)	0.0061 (14)	0.0024 (15)	0.0055 (15)
C27	0.038 (2)	0.037 (2)	0.040 (2)	−0.0165 (19)	0.0203 (19)	−0.0084 (19)

### Bis(4-methylpiperidinium) tetrabromidoaurate(III) bromide (2a) Geometric parameters (Å, º)

**Table:**

Au1—Cl1	2.2752 (8)	C17—H17B	0.9800
Au1—Cl4	2.2802 (7)	C17—H17C	0.9800
Au1—Cl2	2.2872 (8)	N21—C22	1.493 (4)
Au1—Cl3	2.2879 (8)	N21—C26	1.502 (4)
N11—C12	1.483 (4)	N21—H03	0.89 (2)
N11—C16	1.491 (5)	N21—H04	0.89 (2)
N11—H01	0.89 (2)	C22—C23	1.509 (5)
N11—H02	0.88 (2)	C22—H22A	0.9900
C12—C13	1.510 (5)	C22—H22B	0.9900
C12—H12A	0.9900	C23—C24	1.530 (5)
C12—H12B	0.9900	C23—H23A	0.9900
C13—C14	1.524 (5)	C23—H23B	0.9900
C13—H13A	0.9900	C24—C27	1.521 (5)
C13—H13B	0.9900	C24—C25	1.530 (5)
C14—C15	1.526 (4)	C24—H24	1.0000
C14—C17	1.533 (5)	C25—C26	1.507 (5)
C14—H14	1.0000	C25—H25A	0.9900
C15—C16	1.505 (5)	C25—H25B	0.9900
C15—H15A	0.9900	C26—H26A	0.9900
C15—H15B	0.9900	C26—H26B	0.9900
C16—H16A	0.9900	C27—H27A	0.9800
C16—H16B	0.9900	C27—H27B	0.9800
C17—H17A	0.9800	C27—H27C	0.9800
			
Cl1—Au1—Cl4	89.91 (3)	C14—C17—H17C	109.5
Cl1—Au1—Cl2	89.77 (3)	H17A—C17—H17C	109.5
Cl4—Au1—Cl2	176.77 (3)	H17B—C17—H17C	109.5
Cl1—Au1—Cl3	176.43 (3)	C22—N21—C26	112.4 (3)
Cl4—Au1—Cl3	89.80 (3)	C22—N21—H03	105 (3)
Cl2—Au1—Cl3	90.72 (3)	C26—N21—H03	111 (3)
C12—N11—C16	112.8 (3)	C22—N21—H04	110 (3)
C12—N11—H01	105 (3)	C26—N21—H04	108 (3)
C16—N11—H01	112 (3)	H03—N21—H04	110 (4)
C12—N11—H02	111 (3)	N21—C22—C23	110.6 (3)
C16—N11—H02	113 (3)	N21—C22—H22A	109.5
H01—N11—H02	102 (4)	C23—C22—H22A	109.5
N11—C12—C13	109.5 (3)	N21—C22—H22B	109.5
N11—C12—H12A	109.8	C23—C22—H22B	109.5
C13—C12—H12A	109.8	H22A—C22—H22B	108.1
N11—C12—H12B	109.8	C22—C23—C24	113.0 (3)
C13—C12—H12B	109.8	C22—C23—H23A	109.0
H12A—C12—H12B	108.2	C24—C23—H23A	109.0
C12—C13—C14	112.0 (3)	C22—C23—H23B	109.0
C12—C13—H13A	109.2	C24—C23—H23B	109.0
C14—C13—H13A	109.2	H23A—C23—H23B	107.8
C12—C13—H13B	109.2	C27—C24—C25	111.0 (3)
C14—C13—H13B	109.2	C27—C24—C23	110.8 (3)
H13A—C13—H13B	107.9	C25—C24—C23	108.8 (3)
C13—C14—C15	108.8 (3)	C27—C24—H24	108.7
C13—C14—C17	111.6 (3)	C25—C24—H24	108.7
C15—C14—C17	111.3 (3)	C23—C24—H24	108.7
C13—C14—H14	108.4	C26—C25—C24	112.2 (3)
C15—C14—H14	108.4	C26—C25—H25A	109.2
C17—C14—H14	108.4	C24—C25—H25A	109.2
C16—C15—C14	112.4 (3)	C26—C25—H25B	109.2
C16—C15—H15A	109.1	C24—C25—H25B	109.2
C14—C15—H15A	109.1	H25A—C25—H25B	107.9
C16—C15—H15B	109.1	N21—C26—C25	110.1 (3)
C14—C15—H15B	109.1	N21—C26—H26A	109.6
H15A—C15—H15B	107.9	C25—C26—H26A	109.6
N11—C16—C15	110.0 (3)	N21—C26—H26B	109.6
N11—C16—H16A	109.7	C25—C26—H26B	109.6
C15—C16—H16A	109.7	H26A—C26—H26B	108.1
N11—C16—H16B	109.7	C24—C27—H27A	109.5
C15—C16—H16B	109.7	C24—C27—H27B	109.5
H16A—C16—H16B	108.2	H27A—C27—H27B	109.5
C14—C17—H17A	109.5	C24—C27—H27C	109.5
C14—C17—H17B	109.5	H27A—C27—H27C	109.5
H17A—C17—H17B	109.5	H27B—C27—H27C	109.5
			
C16—N11—C12—C13	−57.9 (4)	C26—N21—C22—C23	−55.6 (4)
N11—C12—C13—C14	57.4 (4)	N21—C22—C23—C24	54.7 (4)
C12—C13—C14—C15	−55.7 (4)	C22—C23—C24—C27	−176.3 (3)
C12—C13—C14—C17	−178.9 (3)	C22—C23—C24—C25	−54.0 (4)
C13—C14—C15—C16	54.7 (4)	C27—C24—C25—C26	177.4 (3)
C17—C14—C15—C16	178.1 (3)	C23—C24—C25—C26	55.1 (4)
C12—N11—C16—C15	57.0 (4)	C22—N21—C26—C25	56.9 (4)
C14—C15—C16—N11	−55.4 (4)	C24—C25—C26—N21	−57.0 (4)

### Bis(4-methylpiperidinium) tetrabromidoaurate(III) bromide (2a) Hydrogen-bond geometry (Å, º)

**Table:**

*D*—H···*A*	*D*—H	H···*A*	*D*···*A*	*D*—H···*A*
N11—H01···Cl5	0.89 (2)	2.21 (2)	3.098 (3)	177 (4)
N11—H02···Cl5^i^	0.88 (2)	2.32 (3)	3.145 (3)	157 (4)
N21—H03···Cl3	0.89 (2)	2.80 (3)	3.453 (3)	131 (3)
N21—H04···Cl4	0.89 (2)	2.94 (4)	3.494 (3)	122 (3)
N21—H04···Cl5	0.89 (2)	2.43 (3)	3.144 (3)	138 (3)
N21—H04···Cl5^ii^	0.89 (2)	2.81 (4)	3.239 (3)	111 (3)
C12—H12*A*···Cl2^iii^	0.99	2.85	3.793 (4)	160
C12—H12*A*···Cl3^iii^	0.99	2.91	3.610 (4)	128
C12—H12*B*···Cl1^i^	0.99	2.83	3.794 (4)	164
C12—H12*B*···Cl4^i^	0.99	2.96	3.653 (4)	128
C13—H13*A*···Cl4^ii^	0.99	2.91	3.769 (3)	146
C22—H22*A*···Cl5^ii^	0.99	2.78	3.435 (4)	124

Symmetry codes: (i) −*x*+1, −*y*+2, −*z*+1; (ii) −*x*+1, −*y*+1, −*z*+1; (iii) *x*+1/2, −*y*+3/2, *z*+1/2.

### Bis(4-methylpiperidinium) tetrachloridoaurate(III) chloride (2b) Crystal data

**Table:**

(C_6_H_14_N)_2_[AuCl_4_]Cl	*F*(000) = 2224
*M**_r_* = 574.58	*D*_x_ = 1.874 Mg m^−^^3^
Monoclinic, *P*2/*c*	Mo *K*α radiation, λ = 0.71073 Å
*a* = 18.7771 (8) Å	Cell parameters from 12440 reflections
*b* = 10.6891 (4) Å	θ = 2.7–27.2°
*c* = 20.5603 (9) Å	µ = 7.87 mm^−^^1^
β = 99.284 (5)°	*T* = 100 K
*V* = 4072.6 (3) Å^3^	Plate, yellow
*Z* = 8	0.15 × 0.10 × 0.03 mm

### Bis(4-methylpiperidinium) tetrachloridoaurate(III) chloride (2b) Data collection

**Table:**

Oxford Diffraction Xcalibur, Eos diffractometer	11802 independent reflections
Radiation source: Enhance (Mo) X-ray Source	8670 reflections with *I* > 2σ(*I*)
Detector resolution: 16.1419 pixels mm^-1^	*R*_int_ = 0.115
ω scan	θ_max_ = 30.0°, θ_min_ = 2.2°
Absorption correction: multi-scan (CrysAlisPro; Rigaku OD, 2013)	*h* = −26→26
*T*_min_ = 0.628, *T*_max_ = 1.000	*k* = −14→15
146147 measured reflections	*l* = −28→28

### Bis(4-methylpiperidinium) tetrachloridoaurate(III) chloride (2b) Refinement

**Table:**

Refinement on *F*^2^	Primary atom site location: structure-invariant direct methods
Least-squares matrix: full	Secondary atom site location: difference Fourier map
*R*[*F*^2^ > 2σ(*F*^2^)] = 0.039	Hydrogen site location: mixed
*wR*(*F*^2^) = 0.065	H atoms treated by a mixture of independent and constrained refinement
*S* = 1.04	*w* = 1/[σ^2^(*F*_o_^2^) + (0.015*P*)^2^ + 4.8198*P*] where *P* = (*F*_o_^2^ + 2*F*_c_^2^)/3
11802 reflections	(Δ/σ)_max_ = 0.001
385 parameters	Δρ_max_ = 1.47 e Å^−^^3^
55 restraints	Δρ_min_ = −1.52 e Å^−^^3^

### Bis(4-methylpiperidinium) tetrachloridoaurate(III) chloride (2b) Fractional atomic coordinates and isotropic or equivalent isotropic displacement parameters (Å^2^)

**Table:**

	*x*	*y*	*z*	*U*_iso_*/*U*_eq_	Occ. (<1)
Au1	0.24529 (2)	0.04755 (2)	0.13021 (2)	0.01701 (5)	
Au2	0.25780 (2)	0.49624 (2)	0.38559 (2)	0.01591 (4)	
Cl1	0.12472 (6)	0.02656 (11)	0.13312 (6)	0.0263 (3)	
Cl2	0.26764 (6)	−0.13211 (11)	0.18882 (6)	0.0252 (3)	
Cl3	0.36612 (6)	0.07102 (11)	0.12864 (6)	0.0244 (3)	
Cl4	0.22195 (6)	0.22470 (11)	0.06773 (6)	0.0250 (3)	
Cl5	0.13645 (6)	0.49207 (12)	0.38710 (6)	0.0293 (3)	
Cl6	0.24182 (6)	0.65284 (11)	0.30969 (6)	0.0234 (3)	
Cl7	0.37886 (6)	0.50207 (11)	0.38407 (6)	0.0260 (3)	
Cl8	0.27572 (6)	0.34294 (11)	0.46385 (6)	0.0243 (3)	
Cl9	0.25246 (6)	0.25136 (11)	0.25747 (6)	0.0254 (3)	
Cl10	0.000000	0.20725 (15)	0.250000	0.0235 (4)	
Cl11	0.500000	0.31169 (15)	0.250000	0.0238 (4)	
N11	0.3539 (2)	0.3958 (4)	0.1605 (2)	0.0219 (9)	
H01	0.395 (2)	0.361 (6)	0.183 (3)	0.08 (2)*	
H02	0.3172 (19)	0.339 (4)	0.160 (2)	0.029 (14)*	
C12	0.3367 (3)	0.5092 (5)	0.1970 (3)	0.0295 (12)	
H12A	0.333650	0.486678	0.243135	0.035*	
H12B	0.289246	0.542995	0.176412	0.035*	
C13	0.3945 (3)	0.6082 (4)	0.1958 (2)	0.0266 (11)	
H13A	0.440750	0.577255	0.220565	0.032*	
H13B	0.380906	0.684637	0.218102	0.032*	
C14	0.4044 (2)	0.6404 (4)	0.1256 (3)	0.0241 (11)	
H14	0.358175	0.676706	0.102211	0.029*	
C15	0.4204 (2)	0.5213 (4)	0.0899 (2)	0.0238 (11)	
H15A	0.424070	0.541647	0.043619	0.029*	
H15B	0.467382	0.486906	0.111086	0.029*	
C16	0.3620 (3)	0.4232 (5)	0.0912 (2)	0.0250 (11)	
H16A	0.315700	0.454077	0.066563	0.030*	
H16B	0.375204	0.345683	0.069533	0.030*	
C17	0.4636 (3)	0.7368 (5)	0.1254 (3)	0.0398 (15)	
H17A	0.508700	0.704821	0.150559	0.060*	
H17B	0.450034	0.814468	0.145542	0.060*	
H17C	0.470331	0.753292	0.079887	0.060*	
N21	0.4060 (2)	0.1773 (4)	0.3427 (2)	0.0314 (11)	
H03	0.3593 (14)	0.205 (4)	0.336 (2)	0.027 (14)*	
H04	0.431 (3)	0.227 (4)	0.318 (2)	0.051 (19)*	
C22	0.4314 (3)	0.1771 (5)	0.4146 (2)	0.0271 (12)	
H22A	0.422442	0.260137	0.433006	0.033*	
H22B	0.484036	0.161213	0.423367	0.033*	
C23	0.3926 (3)	0.0771 (4)	0.4479 (2)	0.0254 (11)	
H23A	0.340685	0.098369	0.443102	0.030*	
H23B	0.412265	0.075504	0.495601	0.030*	
C24	0.4008 (2)	−0.0522 (4)	0.4187 (2)	0.0202 (10)	
H24	0.453243	−0.074325	0.426765	0.024*	
C25	0.3759 (3)	−0.0474 (5)	0.3446 (2)	0.0260 (11)	
H25A	0.384721	−0.129638	0.325253	0.031*	
H25B	0.323361	−0.031068	0.335636	0.031*	
C26	0.4148 (3)	0.0533 (5)	0.3119 (3)	0.0341 (13)	
H26A	0.466715	0.032453	0.316499	0.041*	
H26B	0.394932	0.056967	0.264329	0.041*	
C27	0.3602 (3)	−0.1520 (5)	0.4513 (2)	0.0332 (13)	
H27A	0.308502	−0.133034	0.443323	0.050*	
H27B	0.377769	−0.152829	0.498862	0.050*	
H27C	0.368428	−0.234112	0.432664	0.050*	
N31	0.1434 (2)	0.0681 (4)	0.3099 (2)	0.0322 (11)	
H05	0.0981 (16)	0.092 (5)	0.290 (2)	0.045 (17)*	
H06	0.178 (2)	0.102 (5)	0.289 (3)	0.06 (2)*	
C32	0.1549 (3)	0.1151 (5)	0.3785 (3)	0.0385 (15)	
H32A	0.148115	0.206942	0.378270	0.046*	
H32B	0.205093	0.096941	0.399533	0.046*	
C33	0.1030 (3)	0.0546 (5)	0.4178 (3)	0.0313 (12)	
H33A	0.053386	0.082972	0.400478	0.038*	
H33B	0.114730	0.082091	0.464298	0.038*	
C34	0.1056 (2)	−0.0880 (4)	0.4152 (2)	0.0221 (11)	
H34	0.155093	−0.115193	0.435798	0.027*	
C35	0.0935 (3)	−0.1296 (4)	0.3437 (2)	0.0231 (11)	
H35A	0.097893	−0.221819	0.341824	0.028*	
H35B	0.043956	−0.106711	0.322900	0.028*	
C36	0.1470 (3)	−0.0705 (5)	0.3057 (3)	0.0334 (13)	
H36A	0.196358	−0.099095	0.323892	0.040*	
H36B	0.136269	−0.096779	0.258959	0.040*	
C37	0.0521 (3)	−0.1471 (5)	0.4535 (2)	0.0316 (13)	
H37A	0.003109	−0.121608	0.434179	0.047*	
H37B	0.062408	−0.119536	0.499491	0.047*	
H37C	0.056135	−0.238342	0.451541	0.047*	
N41	0.0992 (3)	0.3477 (6)	0.1633 (3)	0.0130 (16)*	0.538 (7)
H41A	0.083171	0.291119	0.190701	0.016*	0.538 (7)
H41B	0.141419	0.318329	0.152894	0.016*	0.538 (7)
C42	0.0459 (5)	0.3571 (8)	0.1026 (4)	0.024 (2)*	0.538 (7)
H42A	−0.002065	0.376160	0.114311	0.029*	0.538 (7)
H42B	0.042474	0.275461	0.079562	0.029*	0.538 (7)
C43	0.0652 (6)	0.4569 (8)	0.0566 (5)	0.036 (3)*	0.538 (7)
H43A	0.025247	0.465499	0.019028	0.043*	0.538 (7)
H43B	0.108774	0.430456	0.038808	0.043*	0.538 (7)
C44	0.0793 (6)	0.5828 (9)	0.0900 (4)	0.016 (3)*	0.538 (7)
H44	0.033537	0.613615	0.103415	0.020*	0.538 (7)
C45	0.1355 (5)	0.5663 (9)	0.1513 (5)	0.039 (3)*	0.538 (7)
H45A	0.143130	0.647300	0.174721	0.047*	0.538 (7)
H45B	0.181908	0.541164	0.138090	0.047*	0.538 (7)
C46	0.1132 (6)	0.4671 (9)	0.1986 (5)	0.040 (3)*	0.538 (7)
H46A	0.152188	0.456149	0.236738	0.048*	0.538 (7)
H46B	0.069182	0.494924	0.215244	0.048*	0.538 (7)
C47	0.1044 (6)	0.6775 (9)	0.0434 (5)	0.022 (2)*	0.538 (7)
H47A	0.150717	0.650696	0.031962	0.034*	0.538 (7)
H47B	0.110110	0.759520	0.064941	0.034*	0.538 (7)
H47C	0.068496	0.683485	0.003231	0.034*	0.538 (7)
N41'	0.0955 (5)	0.4055 (9)	0.1972 (5)	0.035 (3)*	0.462 (7)
H41C	0.073285	0.350302	0.221041	0.042*	0.462 (7)
H41D	0.143855	0.399844	0.211217	0.042*	0.462 (7)
C42'	0.0795 (7)	0.3707 (10)	0.1265 (5)	0.036 (3)*	0.462 (7)
H42C	0.026579	0.365926	0.112565	0.043*	0.462 (7)
H42D	0.100002	0.287057	0.120314	0.043*	0.462 (7)
C43'	0.1110 (6)	0.4657 (8)	0.0837 (5)	0.027 (3)*	0.462 (7)
H43C	0.164283	0.459346	0.092399	0.032*	0.462 (7)
H43D	0.094681	0.444815	0.036754	0.032*	0.462 (7)
C44'	0.0894 (7)	0.5987 (9)	0.0959 (5)	0.016 (3)*	0.462 (7)
H44'	0.036117	0.605794	0.081188	0.019*	0.462 (7)
C45'	0.1045 (5)	0.6279 (9)	0.1687 (4)	0.021 (2)*	0.462 (7)
H45C	0.085175	0.711957	0.176178	0.026*	0.462 (7)
H45D	0.157311	0.629940	0.183454	0.026*	0.462 (7)
C46'	0.0713 (6)	0.5332 (9)	0.2096 (5)	0.032 (3)*	0.462 (7)
H46C	0.085205	0.553499	0.256962	0.038*	0.462 (7)
H46D	0.018022	0.537670	0.198696	0.038*	0.462 (7)
C47'	0.1256 (6)	0.6933 (10)	0.0575 (5)	0.020 (3)*	0.462 (7)
H47D	0.177809	0.692177	0.073001	0.030*	0.462 (7)
H47E	0.106630	0.776904	0.064098	0.030*	0.462 (7)
H47F	0.116000	0.672082	0.010548	0.030*	0.462 (7)

### Bis(4-methylpiperidinium) tetrachloridoaurate(III) chloride (2b) Atomic displacement parameters (Å^2^)

**Table:**

	*U*^11^	*U*^22^	*U*^33^	*U*^12^	*U*^13^	*U*^23^
Au1	0.01700 (8)	0.01640 (9)	0.01697 (9)	−0.00062 (7)	0.00075 (6)	0.00041 (7)
Au2	0.01534 (8)	0.01526 (9)	0.01698 (9)	0.00069 (7)	0.00214 (6)	−0.00016 (7)
Cl1	0.0164 (5)	0.0262 (7)	0.0345 (7)	−0.0040 (5)	−0.0016 (5)	0.0062 (5)
Cl2	0.0267 (6)	0.0203 (6)	0.0286 (7)	0.0041 (5)	0.0047 (5)	0.0049 (5)
Cl3	0.0178 (5)	0.0336 (7)	0.0222 (6)	0.0026 (5)	0.0044 (5)	0.0038 (5)
Cl4	0.0209 (6)	0.0234 (6)	0.0284 (7)	−0.0046 (5)	−0.0025 (5)	0.0085 (5)
Cl5	0.0165 (5)	0.0401 (8)	0.0324 (7)	0.0047 (5)	0.0069 (5)	0.0091 (6)
Cl6	0.0258 (6)	0.0199 (6)	0.0248 (7)	0.0040 (5)	0.0049 (5)	0.0049 (5)
Cl7	0.0159 (5)	0.0233 (6)	0.0385 (7)	0.0003 (5)	0.0032 (5)	0.0059 (6)
Cl8	0.0222 (6)	0.0255 (6)	0.0241 (7)	−0.0009 (5)	0.0003 (5)	0.0078 (5)
Cl9	0.0253 (6)	0.0281 (7)	0.0239 (7)	−0.0107 (5)	0.0077 (5)	−0.0019 (5)
Cl10	0.0184 (8)	0.0246 (9)	0.0274 (10)	0.000	0.0038 (7)	0.000
Cl11	0.0262 (9)	0.0193 (9)	0.0258 (10)	0.000	0.0040 (7)	0.000
N11	0.022 (2)	0.016 (2)	0.029 (3)	−0.0062 (17)	0.0081 (19)	0.0012 (18)
C12	0.039 (3)	0.026 (3)	0.028 (3)	0.000 (2)	0.017 (2)	−0.008 (2)
C13	0.030 (3)	0.018 (3)	0.032 (3)	0.004 (2)	0.005 (2)	−0.003 (2)
C14	0.017 (2)	0.019 (3)	0.035 (3)	0.0001 (19)	0.003 (2)	0.004 (2)
C15	0.023 (2)	0.029 (3)	0.022 (3)	−0.002 (2)	0.008 (2)	0.008 (2)
C16	0.029 (3)	0.026 (3)	0.020 (3)	−0.003 (2)	0.005 (2)	−0.004 (2)
C17	0.032 (3)	0.024 (3)	0.063 (4)	−0.007 (2)	0.008 (3)	0.012 (3)
N21	0.026 (2)	0.033 (3)	0.034 (3)	−0.003 (2)	0.001 (2)	0.017 (2)
C22	0.024 (3)	0.023 (3)	0.034 (3)	0.002 (2)	0.001 (2)	−0.003 (2)
C23	0.029 (3)	0.020 (3)	0.028 (3)	0.003 (2)	0.005 (2)	−0.003 (2)
C24	0.025 (2)	0.019 (2)	0.017 (2)	0.003 (2)	0.0037 (19)	−0.001 (2)
C25	0.033 (3)	0.026 (3)	0.019 (3)	−0.005 (2)	0.005 (2)	−0.004 (2)
C26	0.037 (3)	0.043 (3)	0.023 (3)	−0.007 (3)	0.007 (2)	−0.001 (3)
C27	0.056 (4)	0.026 (3)	0.019 (3)	−0.007 (3)	0.010 (3)	0.002 (2)
N31	0.016 (2)	0.034 (3)	0.048 (3)	0.0022 (19)	0.011 (2)	0.019 (2)
C32	0.025 (3)	0.019 (3)	0.067 (4)	−0.005 (2)	−0.006 (3)	0.003 (3)
C33	0.034 (3)	0.028 (3)	0.030 (3)	−0.006 (2)	0.000 (2)	−0.008 (2)
C34	0.017 (2)	0.021 (3)	0.027 (3)	−0.0014 (19)	−0.001 (2)	0.006 (2)
C35	0.026 (3)	0.019 (3)	0.026 (3)	0.000 (2)	0.007 (2)	0.001 (2)
C36	0.037 (3)	0.024 (3)	0.043 (4)	0.012 (2)	0.017 (3)	0.008 (2)
C37	0.025 (3)	0.045 (4)	0.024 (3)	−0.003 (2)	0.003 (2)	0.007 (2)

### Bis(4-methylpiperidinium) tetrachloridoaurate(III) chloride (2b) Geometric parameters (Å, º)

**Table:**

Au1—Cl2	2.2701 (11)	C33—C34	1.526 (7)
Au1—Cl1	2.2856 (11)	C33—H33A	0.9900
Au1—Cl3	2.2879 (11)	C33—H33B	0.9900
Au1—Cl4	2.2904 (11)	C34—C37	1.510 (6)
Au2—Cl6	2.2751 (11)	C34—C35	1.517 (6)
Au2—Cl7	2.2792 (11)	C34—H34	1.0000
Au2—Cl8	2.2832 (11)	C35—C36	1.509 (7)
Au2—Cl5	2.2842 (11)	C35—H35A	0.9900
N11—C16	1.487 (6)	C35—H35B	0.9900
N11—C12	1.489 (6)	C36—H36A	0.9900
N11—H01	0.91 (2)	C36—H36B	0.9900
N11—H02	0.92 (2)	C37—H37A	0.9800
C12—C13	1.518 (6)	C37—H37B	0.9800
C12—H12A	0.9900	C37—H37C	0.9800
C12—H12B	0.9900	N41—C46	1.470 (10)
C13—C14	1.525 (7)	N41—C42	1.473 (9)
C13—H13A	0.9900	N41—H41A	0.9100
C13—H13B	0.9900	N41—H41B	0.9100
C14—C17	1.516 (6)	C42—C43	1.508 (10)
C14—C15	1.524 (6)	C42—H42A	0.9900
C14—H14	1.0000	C42—H42B	0.9900
C15—C16	1.520 (6)	C43—C44	1.515 (11)
C15—H15A	0.9900	C43—H43A	0.9900
C15—H15B	0.9900	C43—H43B	0.9900
C16—H16A	0.9900	C44—C45	1.519 (10)
C16—H16B	0.9900	C44—C47	1.520 (10)
C17—H17A	0.9800	C44—H44	1.0000
C17—H17B	0.9800	C45—C46	1.541 (11)
C17—H17C	0.9800	C45—H45A	0.9900
N21—C22	1.478 (6)	C45—H45B	0.9900
N21—C26	1.489 (7)	C46—H46A	0.9900
N21—H03	0.91 (2)	C46—H46B	0.9900
N21—H04	0.91 (2)	C47—H47A	0.9800
C22—C23	1.519 (7)	C47—H47B	0.9800
C22—H22A	0.9900	C47—H47C	0.9800
C22—H22B	0.9900	N41'—C46'	1.474 (11)
C23—C24	1.524 (6)	N41'—C42'	1.483 (11)
C23—H23A	0.9900	N41'—H41C	0.9100
C23—H23B	0.9900	N41'—H41D	0.9100
C24—C25	1.521 (6)	C42'—C43'	1.524 (11)
C24—C27	1.527 (6)	C42'—H42C	0.9900
C24—H24	1.0000	C42'—H42D	0.9900
C25—C26	1.517 (7)	C43'—C44'	1.511 (11)
C25—H25A	0.9900	C43'—H43C	0.9900
C25—H25B	0.9900	C43'—H43D	0.9900
C26—H26A	0.9900	C44'—C45'	1.510 (11)
C26—H26B	0.9900	C44'—C47'	1.511 (11)
C27—H27A	0.9800	C44'—H44'	1.0000
C27—H27B	0.9800	C45'—C46'	1.513 (11)
C27—H27C	0.9800	C45'—H45C	0.9900
N31—C32	1.480 (7)	C45'—H45D	0.9900
N31—C36	1.486 (6)	C46'—H46C	0.9900
N31—H05	0.92 (2)	C46'—H46D	0.9900
N31—H06	0.91 (2)	C47'—H47D	0.9800
C32—C33	1.510 (7)	C47'—H47E	0.9800
C32—H32A	0.9900	C47'—H47F	0.9800
C32—H32B	0.9900		
			
Cl2—Au1—Cl1	90.12 (4)	C32—C33—H33B	109.1
Cl2—Au1—Cl3	90.08 (4)	C34—C33—H33B	109.1
Cl1—Au1—Cl3	179.05 (5)	H33A—C33—H33B	107.8
Cl2—Au1—Cl4	177.98 (5)	C37—C34—C35	111.9 (4)
Cl1—Au1—Cl4	89.72 (4)	C37—C34—C33	111.8 (4)
Cl3—Au1—Cl4	90.11 (4)	C35—C34—C33	109.1 (4)
Cl6—Au2—Cl7	89.54 (4)	C37—C34—H34	108.0
Cl6—Au2—Cl8	178.35 (4)	C35—C34—H34	108.0
Cl7—Au2—Cl8	89.74 (4)	C33—C34—H34	108.0
Cl6—Au2—Cl5	90.13 (4)	C36—C35—C34	111.8 (4)
Cl7—Au2—Cl5	179.55 (5)	C36—C35—H35A	109.3
Cl8—Au2—Cl5	90.59 (4)	C34—C35—H35A	109.3
C16—N11—C12	112.8 (4)	C36—C35—H35B	109.3
C16—N11—H01	111 (4)	C34—C35—H35B	109.3
C12—N11—H01	108 (4)	H35A—C35—H35B	107.9
C16—N11—H02	108 (3)	N31—C36—C35	110.3 (4)
C12—N11—H02	109 (3)	N31—C36—H36A	109.6
H01—N11—H02	108 (5)	C35—C36—H36A	109.6
N11—C12—C13	110.2 (4)	N31—C36—H36B	109.6
N11—C12—H12A	109.6	C35—C36—H36B	109.6
C13—C12—H12A	109.6	H36A—C36—H36B	108.1
N11—C12—H12B	109.6	C34—C37—H37A	109.5
C13—C12—H12B	109.6	C34—C37—H37B	109.5
H12A—C12—H12B	108.1	H37A—C37—H37B	109.5
C12—C13—C14	111.6 (4)	C34—C37—H37C	109.5
C12—C13—H13A	109.3	H37A—C37—H37C	109.5
C14—C13—H13A	109.3	H37B—C37—H37C	109.5
C12—C13—H13B	109.3	C46—N41—C42	113.7 (7)
C14—C13—H13B	109.3	C46—N41—H41A	108.8
H13A—C13—H13B	108.0	C42—N41—H41A	108.8
C17—C14—C15	111.4 (4)	C46—N41—H41B	108.8
C17—C14—C13	111.0 (4)	C42—N41—H41B	108.8
C15—C14—C13	109.3 (4)	H41A—N41—H41B	107.7
C17—C14—H14	108.4	N41—C42—C43	112.4 (7)
C15—C14—H14	108.4	N41—C42—H42A	109.1
C13—C14—H14	108.4	C43—C42—H42A	109.1
C16—C15—C14	111.7 (4)	N41—C42—H42B	109.1
C16—C15—H15A	109.3	C43—C42—H42B	109.1
C14—C15—H15A	109.3	H42A—C42—H42B	107.9
C16—C15—H15B	109.3	C42—C43—C44	112.7 (8)
C14—C15—H15B	109.3	C42—C43—H43A	109.0
H15A—C15—H15B	107.9	C44—C43—H43A	109.0
N11—C16—C15	109.7 (4)	C42—C43—H43B	109.0
N11—C16—H16A	109.7	C44—C43—H43B	109.0
C15—C16—H16A	109.7	H43A—C43—H43B	107.8
N11—C16—H16B	109.7	C43—C44—C45	108.7 (8)
C15—C16—H16B	109.7	C43—C44—C47	110.8 (8)
H16A—C16—H16B	108.2	C45—C44—C47	110.9 (8)
C14—C17—H17A	109.5	C43—C44—H44	108.8
C14—C17—H17B	109.5	C45—C44—H44	108.8
H17A—C17—H17B	109.5	C47—C44—H44	108.8
C14—C17—H17C	109.5	C44—C45—C46	112.3 (8)
H17A—C17—H17C	109.5	C44—C45—H45A	109.1
H17B—C17—H17C	109.5	C46—C45—H45A	109.1
C22—N21—C26	112.6 (4)	C44—C45—H45B	109.1
C22—N21—H03	108 (3)	C46—C45—H45B	109.1
C26—N21—H03	113 (3)	H45A—C45—H45B	107.9
C22—N21—H04	116 (4)	N41—C46—C45	109.3 (8)
C26—N21—H04	100 (4)	N41—C46—H46A	109.8
H03—N21—H04	107 (4)	C45—C46—H46A	109.8
N21—C22—C23	110.6 (4)	N41—C46—H46B	109.8
N21—C22—H22A	109.5	C45—C46—H46B	109.8
C23—C22—H22A	109.5	H46A—C46—H46B	108.3
N21—C22—H22B	109.5	C44—C47—H47A	109.5
C23—C22—H22B	109.5	C44—C47—H47B	109.5
H22A—C22—H22B	108.1	H47A—C47—H47B	109.5
C22—C23—C24	111.9 (4)	C44—C47—H47C	109.5
C22—C23—H23A	109.2	H47A—C47—H47C	109.5
C24—C23—H23A	109.2	H47B—C47—H47C	109.5
C22—C23—H23B	109.2	C46'—N41'—C42'	112.5 (9)
C24—C23—H23B	109.2	C46'—N41'—H41C	109.1
H23A—C23—H23B	107.9	C42'—N41'—H41C	109.1
C25—C24—C23	109.3 (4)	C46'—N41'—H41D	109.1
C25—C24—C27	111.6 (4)	C42'—N41'—H41D	109.1
C23—C24—C27	111.7 (4)	H41C—N41'—H41D	107.8
C25—C24—H24	108.0	N41'—C42'—C43'	111.3 (9)
C23—C24—H24	108.0	N41'—C42'—H42C	109.4
C27—C24—H24	108.0	C43'—C42'—H42C	109.4
C26—C25—C24	112.1 (4)	N41'—C42'—H42D	109.4
C26—C25—H25A	109.2	C43'—C42'—H42D	109.4
C24—C25—H25A	109.2	H42C—C42'—H42D	108.0
C26—C25—H25B	109.2	C44'—C43'—C42'	113.0 (8)
C24—C25—H25B	109.2	C44'—C43'—H43C	109.0
H25A—C25—H25B	107.9	C42'—C43'—H43C	109.0
N21—C26—C25	110.4 (4)	C44'—C43'—H43D	109.0
N21—C26—H26A	109.6	C42'—C43'—H43D	109.0
C25—C26—H26A	109.6	H43C—C43'—H43D	107.8
N21—C26—H26B	109.6	C45'—C44'—C43'	110.3 (8)
C25—C26—H26B	109.6	C45'—C44'—C47'	110.6 (9)
H26A—C26—H26B	108.1	C43'—C44'—C47'	112.8 (9)
C24—C27—H27A	109.5	C45'—C44'—H44'	107.7
C24—C27—H27B	109.5	C43'—C44'—H44'	107.7
H27A—C27—H27B	109.5	C47'—C44'—H44'	107.7
C24—C27—H27C	109.5	C44'—C45'—C46'	112.6 (8)
H27A—C27—H27C	109.5	C44'—C45'—H45C	109.1
H27B—C27—H27C	109.5	C46'—C45'—H45C	109.1
C32—N31—C36	113.1 (4)	C44'—C45'—H45D	109.1
C32—N31—H05	108 (3)	C46'—C45'—H45D	109.1
C36—N31—H05	108 (3)	H45C—C45'—H45D	107.8
C32—N31—H06	108 (4)	N41'—C46'—C45'	110.9 (9)
C36—N31—H06	109 (4)	N41'—C46'—H46C	109.5
H05—N31—H06	112 (5)	C45'—C46'—H46C	109.5
N31—C32—C33	110.9 (4)	N41'—C46'—H46D	109.5
N31—C32—H32A	109.5	C45'—C46'—H46D	109.5
C33—C32—H32A	109.5	H46C—C46'—H46D	108.0
N31—C32—H32B	109.5	C44'—C47'—H47D	109.5
C33—C32—H32B	109.5	C44'—C47'—H47E	109.5
H32A—C32—H32B	108.0	H47D—C47'—H47E	109.5
C32—C33—C34	112.5 (4)	C44'—C47'—H47F	109.5
C32—C33—H33A	109.1	H47D—C47'—H47F	109.5
C34—C33—H33A	109.1	H47E—C47'—H47F	109.5
			
C16—N11—C12—C13	−57.1 (5)	C37—C34—C35—C36	180.0 (4)
N11—C12—C13—C14	55.8 (5)	C33—C34—C35—C36	55.8 (5)
C12—C13—C14—C17	−178.5 (4)	C32—N31—C36—C35	56.1 (6)
C12—C13—C14—C15	−55.3 (5)	C34—C35—C36—N31	−56.9 (5)
C17—C14—C15—C16	178.8 (4)	C46—N41—C42—C43	53.8 (10)
C13—C14—C15—C16	55.8 (5)	N41—C42—C43—C44	−52.7 (11)
C12—N11—C16—C15	57.3 (5)	C42—C43—C44—C45	53.3 (11)
C14—C15—C16—N11	−56.6 (5)	C42—C43—C44—C47	175.4 (8)
C26—N21—C22—C23	−56.6 (5)	C43—C44—C45—C46	−55.8 (12)
N21—C22—C23—C24	55.8 (5)	C47—C44—C45—C46	−177.9 (9)
C22—C23—C24—C25	−54.6 (5)	C42—N41—C46—C45	−54.8 (10)
C22—C23—C24—C27	−178.7 (4)	C44—C45—C46—N41	56.7 (12)
C23—C24—C25—C26	54.7 (5)	C46'—N41'—C42'—C43'	54.8 (13)
C27—C24—C25—C26	178.8 (4)	N41'—C42'—C43'—C44'	−52.4 (14)
C22—N21—C26—C25	56.5 (5)	C42'—C43'—C44'—C45'	51.1 (13)
C24—C25—C26—N21	−55.7 (6)	C42'—C43'—C44'—C47'	175.3 (10)
C36—N31—C32—C33	−54.7 (6)	C43'—C44'—C45'—C46'	−52.7 (13)
N31—C32—C33—C34	53.9 (6)	C47'—C44'—C45'—C46'	−178.1 (9)
C32—C33—C34—C37	−178.7 (4)	C42'—N41'—C46'—C45'	−56.4 (12)
C32—C33—C34—C35	−54.4 (5)	C44'—C45'—C46'—N41'	55.6 (12)

### Bis(4-methylpiperidinium) tetrachloridoaurate(III) chloride (2b) Hydrogen-bond geometry (Å, º)

**Table:**

*D*—H···*A*	*D*—H	H···*A*	*D*···*A*	*D*—H···*A*
N11—H02···Cl9	0.92 (2)	2.67 (4)	3.349 (4)	131 (4)
N11—H02···Cl4	0.92 (2)	2.68 (4)	3.405 (4)	136 (4)
N11—H01···Cl11	0.91 (2)	2.28 (3)	3.177 (4)	169 (6)
N21—H03···Cl9	0.91 (2)	2.41 (3)	3.222 (4)	148 (4)
N21—H04···Cl11	0.91 (2)	2.24 (3)	3.146 (5)	169 (5)
N31—H05···Cl10	0.92 (2)	2.26 (3)	3.147 (4)	163 (5)
N31—H06···Cl9	0.91 (2)	2.28 (3)	3.147 (4)	158 (5)
N41—H41*A*···Cl10	0.91	2.31	3.158 (7)	155
N41—H41*B*···Cl4	0.91	2.68	3.517 (7)	152
N41—H41*B*···Cl9	0.91	2.83	3.360 (6)	118
N41′—H41*C*···Cl10	0.91	2.20	3.084 (10)	163
N41′—H41*D*···Cl9	0.91	2.64	3.430 (9)	146
C36—H36*B*···Cl1	0.99	2.88	3.655 (6)	136
C37—H37*A*···Cl1^i^	0.98	3.02	3.966 (5)	163
C37—H37*B*···Cl1^ii^	0.98	2.98	3.942 (5)	168
C42′—H42*D*···Cl1	0.99	2.83	3.773 (11)	160
C13—H13*B*···Cl2^iii^	0.99	2.88	3.645 (5)	135
C26—H26*B*···Cl3	0.99	2.76	3.736 (5)	169
C27—H27*B*···Cl3^ii^	0.98	2.85	3.732 (5)	150
C42′—H42*D*···Cl4	0.99	2.77	3.478 (12)	129
C43′—H43*C*···Cl4	0.99	2.81	3.362 (10)	116
C42—H42*A*···Cl5^i^	0.99	2.81	3.752 (9)	160
C46′—H46*C*···Cl5	0.99	2.77	3.683 (11)	153
C12—H12*A*···Cl6	0.99	2.96	3.497 (5)	115
C36—H36*A*···Cl6^iv^	0.99	2.82	3.446 (5)	122
C46—H46*A*···Cl6	0.99	2.95	3.635 (10)	127
C45′—H45*D*···Cl6	0.99	2.83	3.566 (9)	132
C12—H12*A*···Cl7	0.99	2.89	3.801 (5)	154
C15—H15*B*···Cl7^v^	0.99	2.88	3.727 (5)	144
C27—H27*C*···Cl7^iv^	0.98	3.01	3.983 (5)	173
C14—H14···Cl8^vi^	1.00	3.02	3.787 (5)	134
C23—H23*A*···Cl8	0.99	2.94	3.637 (5)	128
C47—H47*A*···Cl8^vi^	0.98	2.92	3.843 (10)	157
C12—H12*A*···Cl9	0.99	2.98	3.504 (5)	114
C46—H46*A*···Cl9	0.99	2.87	3.550 (11)	126

Symmetry codes: (i) −*x*, *y*, −*z*+1/2; (ii) *x*, −*y*, *z*+1/2; (iii) *x*, *y*+1, *z*; (iv) *x*, *y*−1, *z*; (v) −*x*+1, *y*, −*z*+1/2; (vi) *x*, −*y*+1, *z*−1/2.

### Bis(4-methylpiperidinium) tetrachloridoaurate(III) chloride (2c) Crystal data

**Table:**

(C_6_H_14_N)_2_[AuCl_4_]Cl	*Z* = 10
*M**_r_* = 574.58	*F*(000) = 2780
Triclinic, *P*1	*D*_x_ = 1.887 Mg m^−^^3^
*a* = 14.4553 (6) Å	Mo *K*α radiation, λ = 0.71073 Å
*b* = 15.1302 (5) Å	Cell parameters from 31287 reflections
*c* = 24.3885 (6) Å	θ = 2.4–29.3°
α = 90.797 (3)°	µ = 7.93 mm^−^^1^
β = 98.137 (3)°	*T* = 100 K
γ = 106.407 (4)°	Block, yellow
*V* = 5057.2 (3) Å^3^	0.2 × 0.1 × 0.08 mm

### Bis(4-methylpiperidinium) tetrachloridoaurate(III) chloride (2c) Data collection

**Table:**

Oxford Diffraction Xcalibur, Eos diffractometer	29180 independent reflections
Radiation source: Enhance (Mo) X-ray Source	22450 reflections with *I* > 2σ(*I*)
Detector resolution: 16.1419 pixels mm^-1^	*R*_int_ = 0.098
ω scan	θ_max_ = 30.0°, θ_min_ = 2.3°
Absorption correction: multi-scan (CrysAlisPro; Rigaku OD, 2013)	*h* = −20→20
*T*_min_ = 0.683, *T*_max_ = 1.000	*k* = −21→21
316587 measured reflections	*l* = −34→34

### Bis(4-methylpiperidinium) tetrachloridoaurate(III) chloride (2c) Refinement

**Table:**

Refinement on *F*^2^	Primary atom site location: structure-invariant direct methods
Least-squares matrix: full	Secondary atom site location: difference Fourier map
*R*[*F*^2^ > 2σ(*F*^2^)] = 0.041	Hydrogen site location: mixed
*wR*(*F*^2^) = 0.084	H atoms treated by a mixture of independent and constrained refinement
*S* = 1.06	*w* = 1/[σ^2^(*F*_o_^2^) + (0.0268*P*)^2^ + 13.6626*P*] where *P* = (*F*_o_^2^ + 2*F*_c_^2^)/3
29180 reflections	(Δ/σ)_max_ = 0.002
967 parameters	Δρ_max_ = 2.67 e Å^−^^3^
64 restraints	Δρ_min_ = −1.82 e Å^−^^3^

### Bis(4-methylpiperidinium) tetrachloridoaurate(III) chloride (2c) Fractional atomic coordinates and isotropic or equivalent isotropic displacement parameters (Å^2^)

**Table:**

	*x*	*y*	*z*	*U*_iso_*/*U*_eq_	
Au1	0.11750 (2)	0.63845 (2)	0.26925 (2)	0.01458 (4)	
Cl1	0.10877 (10)	0.48662 (9)	0.27627 (6)	0.0228 (3)	
Cl2	0.23608 (10)	0.65461 (9)	0.21364 (6)	0.0240 (3)	
Cl3	0.12269 (11)	0.78873 (9)	0.26076 (6)	0.0272 (3)	
Cl4	0.00259 (10)	0.62298 (9)	0.32607 (6)	0.0224 (3)	
Au2	−0.05782 (2)	0.88299 (2)	0.71855 (2)	0.01638 (5)	
Cl5	−0.05144 (11)	0.73445 (9)	0.72336 (6)	0.0248 (3)	
Cl6	−0.15343 (10)	0.86170 (10)	0.78731 (6)	0.0248 (3)	
Cl7	−0.06299 (11)	1.03123 (9)	0.71577 (6)	0.0286 (3)	
Cl8	0.03434 (11)	0.90231 (9)	0.64835 (6)	0.0250 (3)	
Au3	0.50187 (2)	0.75319 (2)	0.49964 (2)	0.01549 (4)	
Cl9	0.51949 (11)	0.90589 (9)	0.49301 (6)	0.0297 (3)	
Cl10	0.60967 (10)	0.76302 (9)	0.43888 (6)	0.0265 (3)	
Cl11	0.49027 (11)	0.60171 (9)	0.50970 (7)	0.0299 (3)	
Cl12	0.39100 (12)	0.74279 (10)	0.55928 (6)	0.0315 (3)	
Au4	0.71049 (2)	1.03097 (2)	0.12450 (2)	0.01662 (5)	
Cl13	0.79786 (10)	1.07813 (10)	0.05394 (6)	0.0261 (3)	
Cl14	0.85352 (10)	1.03877 (11)	0.18181 (6)	0.0272 (3)	
Cl15	0.62278 (10)	0.98439 (10)	0.19570 (6)	0.0243 (3)	
Cl16	0.56937 (10)	1.02724 (10)	0.06646 (6)	0.0244 (3)	
Au5	0.72767 (2)	0.55071 (2)	0.11465 (2)	0.01646 (5)	
Cl17	0.58038 (10)	0.53456 (10)	0.06141 (6)	0.0254 (3)	
Cl18	0.64953 (12)	0.49818 (12)	0.18783 (6)	0.0354 (4)	
Cl19	0.87584 (10)	0.56492 (10)	0.16812 (6)	0.0273 (3)	
Cl20	0.80491 (10)	0.60417 (10)	0.04130 (5)	0.0269 (3)	
Cl21	0.89073 (10)	0.81577 (9)	−0.03711 (5)	0.0211 (3)	
Cl22	0.69597 (9)	0.80536 (9)	0.10738 (5)	0.0196 (3)	
Cl23	0.51959 (10)	0.72230 (10)	0.25681 (6)	0.0255 (3)	
Cl24	0.29808 (10)	0.70124 (9)	0.38233 (6)	0.0239 (3)	
Cl25	0.08329 (10)	0.67624 (10)	0.52152 (6)	0.0271 (3)	
N11	0.9469 (4)	0.7232 (3)	−0.13518 (19)	0.0228 (10)	
H01	0.934 (4)	0.746 (4)	−0.1040 (14)	0.028 (17)*	
H02	0.952 (6)	0.768 (4)	−0.159 (2)	0.07 (3)*	
C12	0.8550 (4)	0.6481 (4)	−0.1547 (2)	0.0232 (12)	
H12A	0.857057	0.624359	−0.192447	0.028*	
H12B	0.798496	0.673348	−0.156771	0.028*	
C13	0.8424 (4)	0.5700 (4)	−0.1158 (2)	0.0222 (12)	
H13A	0.783834	0.519213	−0.131105	0.027*	
H13B	0.831874	0.592040	−0.079459	0.027*	
C14	0.9317 (4)	0.5333 (4)	−0.1077 (2)	0.0230 (12)	
H14	0.939656	0.509503	−0.144658	0.028*	
C15	1.0217 (4)	0.6126 (4)	−0.0870 (2)	0.0248 (12)	
H15A	1.015558	0.636916	−0.050240	0.030*	
H15B	1.079916	0.589666	−0.082514	0.030*	
C16	1.0358 (4)	0.6898 (4)	−0.1265 (2)	0.0256 (12)	
H16A	1.093473	0.741328	−0.111125	0.031*	
H16B	1.047446	0.667322	−0.162531	0.031*	
C17	0.9187 (5)	0.4541 (4)	−0.0691 (3)	0.0334 (14)	
H17A	0.978182	0.434396	−0.063436	0.050*	
H17B	0.863539	0.402287	−0.085754	0.050*	
H17C	0.905851	0.474504	−0.033397	0.050*	
N21	0.9236 (3)	0.8811 (3)	0.09055 (19)	0.0230 (10)	
H03	0.908 (3)	0.854 (3)	0.0560 (12)	0.006 (13)*	
H04	0.867 (2)	0.890 (3)	0.0969 (18)	0.007 (13)*	
C22	0.9501 (4)	0.8124 (4)	0.1287 (2)	0.0246 (12)	
H22A	0.894977	0.755292	0.125761	0.030*	
H22B	0.964069	0.838061	0.167567	0.030*	
C23	1.0396 (4)	0.7905 (4)	0.1130 (2)	0.0235 (12)	
H23A	1.024066	0.761602	0.074836	0.028*	
H23B	1.058082	0.745866	0.138467	0.028*	
C24	1.1252 (4)	0.8777 (4)	0.1159 (2)	0.0206 (11)	
H24	1.142003	0.904353	0.155015	0.025*	
C25	1.0951 (4)	0.9477 (4)	0.0791 (2)	0.0222 (12)	
H25A	1.149303	1.005636	0.083024	0.027*	
H25B	1.082981	0.923940	0.039937	0.027*	
C26	1.0046 (4)	0.9683 (4)	0.0931 (3)	0.0240 (12)	
H26A	1.018350	0.998520	0.130825	0.029*	
H26B	0.985254	1.011029	0.066515	0.029*	
C27	1.2136 (4)	0.8539 (5)	0.1000 (3)	0.0341 (15)	
H27A	1.228839	0.806838	0.123944	0.051*	
H27B	1.269563	0.909372	0.104524	0.051*	
H27C	1.199331	0.830018	0.061202	0.051*	
N31	0.6735 (4)	0.7700 (4)	−0.0214 (2)	0.0324 (13)	
H05	0.735 (2)	0.773 (5)	−0.027 (2)	0.05 (2)*	
H06	0.675 (5)	0.766 (5)	0.0155 (11)	0.05 (2)*	
C32	0.6029 (4)	0.6873 (4)	−0.0502 (2)	0.0294 (13)	
H32A	0.539750	0.676899	−0.036330	0.035*	
H32B	0.626737	0.632834	−0.042232	0.035*	
C33	0.5883 (4)	0.6978 (4)	−0.1122 (2)	0.0268 (13)	
H33A	0.649483	0.699797	−0.126620	0.032*	
H33B	0.536783	0.643353	−0.130393	0.032*	
C34	0.5597 (4)	0.7843 (4)	−0.1271 (2)	0.0231 (12)	
H34	0.496265	0.779575	−0.113799	0.028*	
C35	0.6360 (5)	0.8682 (4)	−0.0966 (3)	0.0310 (14)	
H35A	0.698713	0.876202	−0.110638	0.037*	
H35B	0.614852	0.924127	−0.104345	0.037*	
C36	0.6508 (5)	0.8576 (4)	−0.0345 (3)	0.0362 (16)	
H36A	0.704896	0.910136	−0.016360	0.043*	
H36B	0.590855	0.858692	−0.019524	0.043*	
C37	0.5452 (5)	0.7948 (5)	−0.1895 (2)	0.0426 (18)	
H37A	0.525220	0.850682	−0.197315	0.064*	
H37B	0.494502	0.740894	−0.207775	0.064*	
H37C	0.606518	0.799680	−0.203670	0.064*	
N41	0.7399 (3)	0.8221 (3)	0.24105 (19)	0.0221 (10)	
H07	0.677 (2)	0.801 (4)	0.245 (2)	0.027 (17)*	
H08	0.744 (5)	0.857 (5)	0.212 (2)	0.07 (3)*	
C42	0.7940 (5)	0.8916 (4)	0.2875 (2)	0.0321 (14)	
H42A	0.761717	0.940989	0.289160	0.038*	
H42B	0.861588	0.920047	0.280577	0.038*	
C43	0.7959 (5)	0.8454 (4)	0.3423 (2)	0.0309 (14)	
H43A	0.728516	0.822475	0.350861	0.037*	
H43B	0.834578	0.891310	0.372262	0.037*	
C44	0.8400 (4)	0.7647 (4)	0.3409 (2)	0.0246 (12)	
H44	0.909122	0.789604	0.334269	0.029*	
C45	0.7840 (4)	0.6965 (4)	0.2925 (2)	0.0252 (12)	
H45A	0.814948	0.646250	0.290106	0.030*	
H45B	0.716172	0.668796	0.299168	0.030*	
C46	0.7830 (5)	0.7437 (4)	0.2383 (2)	0.0298 (14)	
H46A	0.850430	0.766937	0.229967	0.036*	
H46B	0.744311	0.698532	0.207901	0.036*	
C47	0.8403 (5)	0.7178 (5)	0.3955 (2)	0.0348 (15)	
H47A	0.773541	0.696410	0.403934	0.052*	
H47B	0.881376	0.761666	0.425194	0.052*	
H47C	0.866227	0.664882	0.392609	0.052*	
N51	0.4789 (4)	0.7064 (4)	0.1272 (2)	0.0301 (12)	
H09	0.537 (3)	0.717 (5)	0.113 (2)	0.05 (2)*	
H010	0.501 (5)	0.718 (6)	0.1650 (11)	0.08 (3)*	
C52	0.4032 (4)	0.6177 (4)	0.1104 (2)	0.0292 (13)	
H52A	0.347409	0.612742	0.130737	0.035*	
H52B	0.430313	0.565823	0.120134	0.035*	
C53	0.3683 (4)	0.6119 (4)	0.0485 (2)	0.0275 (13)	
H53A	0.314619	0.554221	0.038550	0.033*	
H53B	0.422432	0.609072	0.028503	0.033*	
C54	0.3322 (4)	0.6943 (4)	0.0298 (2)	0.0221 (12)	
H54	0.273391	0.692619	0.047397	0.027*	
C55	0.4104 (4)	0.7832 (4)	0.0496 (2)	0.0266 (13)	
H55A	0.384364	0.835931	0.040511	0.032*	
H55B	0.466547	0.788642	0.029536	0.032*	
C56	0.4455 (5)	0.7883 (4)	0.1114 (3)	0.0321 (14)	
H56A	0.499961	0.845276	0.121794	0.038*	
H56B	0.391809	0.790964	0.131910	0.038*	
C57	0.3028 (4)	0.6883 (4)	−0.0330 (2)	0.0308 (14)	
H57A	0.279925	0.741453	−0.044032	0.046*	
H57B	0.250307	0.631217	−0.044214	0.046*	
H57C	0.359193	0.688297	−0.051031	0.046*	
N61	0.4047 (5)	0.8541 (4)	0.2973 (2)	0.0456 (16)	
H61B	0.425898	0.808024	0.284001	0.055*	
H61A	0.353360	0.827802	0.315117	0.055*	
C62	0.4844 (7)	0.9149 (6)	0.3377 (3)	0.054 (2)	
H62A	0.542808	0.939348	0.319590	0.065*	
H62B	0.501915	0.878724	0.369004	0.065*	
C63	0.4525 (7)	0.9952 (6)	0.3598 (3)	0.059 (2)	
H63A	0.400573	0.970957	0.382730	0.071*	
H63B	0.508623	1.038075	0.383840	0.071*	
C64	0.4152 (5)	1.0471 (4)	0.3136 (2)	0.0289 (13)	
H64	0.470928	1.077495	0.293794	0.035*	
C65	0.3374 (5)	0.9814 (5)	0.2723 (3)	0.0439 (18)	
H65A	0.317848	1.016149	0.240832	0.053*	
H65B	0.279240	0.954860	0.290354	0.053*	
C66	0.3712 (5)	0.9034 (5)	0.2503 (3)	0.0376 (16)	
H66A	0.316866	0.860190	0.225240	0.045*	
H66B	0.425226	0.928587	0.228830	0.045*	
C67	0.3758 (6)	1.1222 (5)	0.3352 (3)	0.0446 (18)	
H67A	0.346560	1.150081	0.303811	0.067*	
H67B	0.429249	1.169706	0.357211	0.067*	
H67C	0.326158	1.095027	0.358468	0.067*	
N71	0.3752 (4)	0.5584 (4)	0.3128 (2)	0.0291 (11)	
H71A	0.409481	0.602672	0.292562	0.035*	
H71B	0.321284	0.574327	0.318997	0.035*	
C72	0.3435 (5)	0.4690 (4)	0.2794 (3)	0.0340 (15)	
H72A	0.309776	0.477073	0.242407	0.041*	
H72B	0.296948	0.422653	0.298041	0.041*	
C73	0.4306 (5)	0.4351 (5)	0.2729 (3)	0.0364 (15)	
H73A	0.472132	0.477287	0.249602	0.044*	
H73B	0.407367	0.373198	0.253357	0.044*	
C74	0.4913 (5)	0.4294 (5)	0.3280 (2)	0.0321 (14)	
H74	0.449533	0.383693	0.350091	0.039*	
C75	0.5224 (4)	0.5242 (4)	0.3602 (2)	0.0261 (13)	
H75A	0.558010	0.519334	0.397285	0.031*	
H75B	0.567328	0.569615	0.340133	0.031*	
C76	0.4361 (5)	0.5576 (5)	0.3668 (3)	0.0351 (15)	
H76A	0.396098	0.517080	0.391422	0.042*	
H76B	0.459184	0.620810	0.384586	0.042*	
C77	0.5782 (5)	0.3965 (6)	0.3200 (3)	0.0472 (19)	
H77A	0.555726	0.336802	0.298973	0.071*	
H77B	0.613735	0.389893	0.356325	0.071*	
H77C	0.621462	0.441595	0.299625	0.071*	
N81	0.1936 (4)	0.8210 (4)	0.4435 (2)	0.0383 (15)	
H81B	0.155611	0.782620	0.465134	0.046*	
H81A	0.221498	0.786522	0.423938	0.046*	
C82	0.2710 (5)	0.8907 (6)	0.4794 (3)	0.051 (2)	
H82A	0.317605	0.927738	0.456559	0.061*	
H82B	0.307168	0.859495	0.506423	0.061*	
C83	0.2296 (5)	0.9530 (5)	0.5099 (3)	0.0432 (18)	
H83A	0.283438	1.000769	0.532390	0.052*	
H83B	0.188062	0.916767	0.535487	0.052*	
C84	0.1695 (4)	0.9993 (4)	0.4706 (3)	0.0302 (14)	
H84	0.212788	1.035969	0.445379	0.036*	
C85	0.0898 (5)	0.9251 (4)	0.4357 (2)	0.0297 (14)	
H85A	0.050836	0.954391	0.409049	0.036*	
H85B	0.045884	0.888329	0.459929	0.036*	
C86	0.1307 (5)	0.8616 (4)	0.4039 (3)	0.0306 (14)	
H86A	0.076552	0.811682	0.383266	0.037*	
H86B	0.169456	0.896724	0.376803	0.037*	
C87	0.1292 (5)	1.0648 (5)	0.5011 (3)	0.0419 (17)	
H87A	0.084531	1.029921	0.525078	0.063*	
H87B	0.093891	1.095747	0.474114	0.063*	
H87C	0.183030	1.111007	0.523738	0.063*	
N91	0.2027 (4)	0.5556 (3)	0.4758 (2)	0.0269 (11)	
H017	0.254 (5)	0.590 (5)	0.459 (3)	0.10 (3)*	
H018	0.179 (5)	0.601 (4)	0.490 (3)	0.07 (3)*	
C92	0.2369 (5)	0.5005 (4)	0.5205 (2)	0.0302 (14)	
H92A	0.183364	0.473461	0.541900	0.036*	
H92B	0.292081	0.541207	0.546250	0.036*	
C93	0.2688 (4)	0.4248 (4)	0.4954 (2)	0.0264 (13)	
H93A	0.289633	0.387990	0.525482	0.032*	
H93B	0.325910	0.452658	0.476835	0.032*	
C94	0.1894 (4)	0.3611 (4)	0.4539 (2)	0.0265 (13)	
H94	0.133933	0.330215	0.473683	0.032*	
C95	0.1529 (4)	0.4198 (4)	0.4097 (2)	0.0244 (12)	
H95A	0.205373	0.445982	0.387405	0.029*	
H95B	0.096681	0.379536	0.384415	0.029*	
C96	0.1225 (4)	0.4975 (4)	0.4339 (2)	0.0271 (13)	
H96A	0.105398	0.536084	0.403920	0.033*	
H96B	0.063876	0.471733	0.451748	0.033*	
C97	0.2236 (5)	0.2872 (4)	0.4275 (3)	0.0369 (15)	
H97A	0.168843	0.245662	0.402704	0.055*	
H97B	0.248177	0.251887	0.456608	0.055*	
H97C	0.275786	0.316068	0.406291	0.055*	
N101	0.1281 (4)	0.7149 (3)	0.6481 (2)	0.0245 (10)	
H019	0.081 (4)	0.741 (5)	0.659 (2)	0.06 (2)*	
H020	0.107 (4)	0.704 (4)	0.6100 (11)	0.019 (15)*	
C102	0.1313 (4)	0.6254 (4)	0.6715 (2)	0.0255 (12)	
H10A	0.178534	0.601533	0.654676	0.031*	
H10B	0.066173	0.579831	0.662541	0.031*	
C103	0.1615 (4)	0.6384 (4)	0.7345 (2)	0.0268 (13)	
H10C	0.109249	0.654011	0.751332	0.032*	
H10D	0.168299	0.579464	0.749062	0.032*	
C104	0.2574 (4)	0.7142 (4)	0.7518 (2)	0.0266 (13)	
H10E	0.311341	0.693546	0.739319	0.032*	
C105	0.2529 (5)	0.8032 (4)	0.7233 (3)	0.0309 (14)	
H10F	0.205301	0.828533	0.738773	0.037*	
H10G	0.317620	0.849601	0.731408	0.037*	
C106	0.2235 (5)	0.7871 (4)	0.6609 (2)	0.0297 (14)	
H10H	0.218285	0.845197	0.644422	0.036*	
H10I	0.273836	0.767189	0.644675	0.036*	
C107	0.2789 (5)	0.7311 (5)	0.8141 (2)	0.0347 (15)	
H10J	0.340238	0.780212	0.824249	0.052*	
H10K	0.284630	0.674344	0.831310	0.052*	
H10L	0.225729	0.749670	0.827194	0.052*	

### Bis(4-methylpiperidinium) tetrachloridoaurate(III) chloride (2c) Atomic displacement parameters (Å^2^)

**Table:**

	*U*^11^	*U*^22^	*U*^33^	*U*^12^	*U*^13^	*U*^23^
Au1	0.01767 (10)	0.01336 (9)	0.01383 (9)	0.00604 (7)	0.00287 (7)	0.00030 (7)
Cl1	0.0305 (7)	0.0147 (6)	0.0268 (7)	0.0087 (5)	0.0109 (6)	0.0039 (5)
Cl2	0.0287 (7)	0.0225 (7)	0.0233 (7)	0.0069 (6)	0.0132 (6)	0.0012 (5)
Cl3	0.0440 (9)	0.0166 (6)	0.0258 (7)	0.0136 (6)	0.0108 (6)	0.0045 (5)
Cl4	0.0210 (7)	0.0231 (7)	0.0273 (7)	0.0103 (5)	0.0097 (5)	0.0020 (5)
Au2	0.01852 (10)	0.01361 (9)	0.01754 (10)	0.00654 (7)	0.00062 (8)	0.00070 (7)
Cl5	0.0409 (8)	0.0156 (6)	0.0221 (7)	0.0113 (6)	0.0118 (6)	0.0030 (5)
Cl6	0.0298 (7)	0.0251 (7)	0.0242 (7)	0.0126 (6)	0.0093 (6)	0.0038 (5)
Cl7	0.0339 (8)	0.0179 (7)	0.0412 (9)	0.0157 (6)	0.0119 (7)	0.0073 (6)
Cl8	0.0314 (8)	0.0219 (7)	0.0250 (7)	0.0098 (6)	0.0102 (6)	0.0050 (5)
Au3	0.01522 (10)	0.01378 (9)	0.01862 (10)	0.00552 (7)	0.00353 (8)	0.00166 (7)
Cl9	0.0379 (8)	0.0151 (6)	0.0385 (8)	0.0100 (6)	0.0085 (7)	0.0019 (6)
Cl10	0.0232 (7)	0.0233 (7)	0.0363 (8)	0.0066 (6)	0.0159 (6)	0.0036 (6)
Cl11	0.0321 (8)	0.0159 (6)	0.0467 (9)	0.0105 (6)	0.0145 (7)	0.0074 (6)
Cl12	0.0373 (9)	0.0312 (8)	0.0328 (8)	0.0132 (7)	0.0206 (7)	0.0042 (6)
Au4	0.01613 (10)	0.01694 (10)	0.01605 (10)	0.00409 (7)	0.00157 (7)	−0.00141 (7)
Cl13	0.0282 (7)	0.0275 (7)	0.0212 (7)	0.0038 (6)	0.0077 (6)	0.0025 (6)
Cl14	0.0186 (7)	0.0412 (8)	0.0208 (7)	0.0076 (6)	0.0020 (5)	−0.0015 (6)
Cl15	0.0219 (7)	0.0279 (7)	0.0217 (7)	0.0032 (5)	0.0066 (5)	−0.0031 (5)
Cl16	0.0166 (6)	0.0261 (7)	0.0275 (7)	0.0050 (5)	−0.0044 (5)	0.0038 (6)
Au5	0.01774 (10)	0.01631 (10)	0.01430 (10)	0.00262 (7)	0.00379 (7)	0.00045 (7)
Cl17	0.0200 (7)	0.0303 (7)	0.0242 (7)	0.0056 (6)	0.0017 (5)	−0.0003 (6)
Cl18	0.0326 (8)	0.0455 (9)	0.0202 (7)	−0.0043 (7)	0.0097 (6)	0.0046 (6)
Cl19	0.0225 (7)	0.0313 (8)	0.0245 (7)	0.0037 (6)	−0.0009 (6)	0.0082 (6)
Cl20	0.0242 (7)	0.0357 (8)	0.0177 (7)	0.0016 (6)	0.0074 (5)	0.0011 (6)
Cl21	0.0228 (7)	0.0228 (6)	0.0180 (6)	0.0047 (5)	0.0082 (5)	−0.0002 (5)
Cl22	0.0162 (6)	0.0249 (7)	0.0160 (6)	0.0026 (5)	0.0035 (5)	−0.0020 (5)
Cl23	0.0208 (7)	0.0356 (8)	0.0217 (7)	0.0078 (6)	0.0081 (5)	0.0052 (6)
Cl24	0.0260 (7)	0.0257 (7)	0.0240 (7)	0.0110 (6)	0.0097 (6)	0.0005 (5)
Cl25	0.0270 (7)	0.0339 (8)	0.0218 (7)	0.0071 (6)	0.0118 (6)	0.0027 (6)
N11	0.033 (3)	0.023 (2)	0.014 (2)	0.007 (2)	0.010 (2)	0.0019 (19)
C12	0.021 (3)	0.029 (3)	0.021 (3)	0.010 (2)	0.003 (2)	0.000 (2)
C13	0.018 (3)	0.028 (3)	0.020 (3)	0.005 (2)	0.008 (2)	0.004 (2)
C14	0.025 (3)	0.022 (3)	0.022 (3)	0.005 (2)	0.003 (2)	0.000 (2)
C15	0.024 (3)	0.027 (3)	0.024 (3)	0.010 (2)	0.000 (2)	0.004 (2)
C16	0.022 (3)	0.028 (3)	0.024 (3)	0.000 (2)	0.007 (2)	−0.002 (2)
C17	0.039 (4)	0.028 (3)	0.032 (3)	0.008 (3)	0.005 (3)	0.011 (3)
N21	0.021 (2)	0.030 (3)	0.018 (2)	0.005 (2)	0.008 (2)	−0.003 (2)
C22	0.023 (3)	0.027 (3)	0.022 (3)	0.002 (2)	0.008 (2)	0.005 (2)
C23	0.027 (3)	0.018 (3)	0.025 (3)	0.005 (2)	0.007 (2)	0.006 (2)
C24	0.018 (3)	0.019 (3)	0.026 (3)	0.006 (2)	0.005 (2)	0.001 (2)
C25	0.016 (3)	0.021 (3)	0.033 (3)	0.007 (2)	0.011 (2)	0.008 (2)
C26	0.017 (3)	0.021 (3)	0.035 (3)	0.005 (2)	0.008 (2)	0.004 (2)
C27	0.025 (3)	0.036 (4)	0.045 (4)	0.014 (3)	0.010 (3)	0.011 (3)
N31	0.017 (3)	0.058 (4)	0.018 (3)	0.002 (2)	0.005 (2)	0.001 (2)
C32	0.025 (3)	0.038 (4)	0.026 (3)	0.007 (3)	0.011 (3)	0.009 (3)
C33	0.025 (3)	0.028 (3)	0.023 (3)	0.000 (2)	0.006 (2)	−0.004 (2)
C34	0.016 (3)	0.030 (3)	0.021 (3)	0.002 (2)	0.004 (2)	0.002 (2)
C35	0.030 (3)	0.026 (3)	0.035 (4)	0.000 (3)	0.016 (3)	−0.002 (3)
C36	0.034 (4)	0.034 (4)	0.030 (3)	−0.009 (3)	0.012 (3)	−0.018 (3)
C37	0.033 (4)	0.067 (5)	0.022 (3)	0.004 (3)	0.006 (3)	0.015 (3)
N41	0.019 (2)	0.029 (3)	0.017 (2)	0.004 (2)	0.0044 (19)	−0.001 (2)
C42	0.036 (4)	0.026 (3)	0.027 (3)	0.002 (3)	0.000 (3)	0.000 (3)
C43	0.047 (4)	0.031 (3)	0.016 (3)	0.015 (3)	0.000 (3)	−0.001 (2)
C44	0.023 (3)	0.031 (3)	0.022 (3)	0.009 (2)	0.009 (2)	0.008 (2)
C45	0.019 (3)	0.028 (3)	0.031 (3)	0.009 (2)	0.004 (2)	0.001 (2)
C46	0.037 (4)	0.041 (4)	0.019 (3)	0.025 (3)	0.002 (3)	−0.007 (3)
C47	0.045 (4)	0.045 (4)	0.025 (3)	0.025 (3)	0.014 (3)	0.013 (3)
N51	0.019 (3)	0.049 (3)	0.016 (3)	0.001 (2)	0.002 (2)	−0.002 (2)
C52	0.024 (3)	0.037 (4)	0.029 (3)	0.009 (3)	0.013 (3)	0.005 (3)
C53	0.028 (3)	0.035 (3)	0.020 (3)	0.010 (3)	0.006 (2)	−0.003 (2)
C54	0.011 (3)	0.028 (3)	0.027 (3)	0.005 (2)	0.003 (2)	−0.007 (2)
C55	0.018 (3)	0.031 (3)	0.027 (3)	0.002 (2)	0.002 (2)	−0.001 (2)
C56	0.025 (3)	0.028 (3)	0.035 (4)	−0.005 (3)	0.004 (3)	−0.010 (3)
C57	0.025 (3)	0.035 (4)	0.031 (3)	0.012 (3)	−0.007 (3)	−0.003 (3)
N61	0.069 (4)	0.027 (3)	0.047 (4)	0.010 (3)	0.039 (3)	0.003 (3)
C62	0.091 (7)	0.066 (6)	0.021 (4)	0.050 (5)	0.005 (4)	0.010 (3)
C63	0.110 (8)	0.059 (5)	0.024 (4)	0.051 (5)	0.010 (4)	0.007 (3)
C64	0.038 (4)	0.022 (3)	0.021 (3)	0.003 (3)	0.000 (3)	−0.002 (2)
C65	0.033 (4)	0.040 (4)	0.055 (5)	0.008 (3)	0.005 (3)	−0.014 (3)
C66	0.026 (3)	0.041 (4)	0.045 (4)	0.007 (3)	0.010 (3)	−0.006 (3)
C67	0.064 (5)	0.034 (4)	0.037 (4)	0.020 (4)	0.001 (4)	−0.003 (3)
N71	0.027 (3)	0.037 (3)	0.028 (3)	0.014 (2)	0.009 (2)	0.002 (2)
C72	0.033 (4)	0.032 (4)	0.039 (4)	0.014 (3)	−0.001 (3)	0.001 (3)
C73	0.044 (4)	0.042 (4)	0.028 (3)	0.021 (3)	0.004 (3)	−0.004 (3)
C74	0.039 (4)	0.041 (4)	0.027 (3)	0.026 (3)	0.009 (3)	0.004 (3)
C75	0.025 (3)	0.032 (3)	0.023 (3)	0.010 (3)	0.005 (2)	0.001 (2)
C76	0.032 (4)	0.041 (4)	0.035 (4)	0.019 (3)	−0.002 (3)	−0.013 (3)
C77	0.051 (5)	0.069 (5)	0.036 (4)	0.038 (4)	0.011 (3)	0.015 (4)
N81	0.052 (4)	0.035 (3)	0.048 (3)	0.028 (3)	0.038 (3)	0.020 (3)
C82	0.032 (4)	0.088 (6)	0.046 (4)	0.035 (4)	0.012 (3)	0.025 (4)
C83	0.027 (4)	0.068 (5)	0.033 (4)	0.014 (3)	−0.003 (3)	0.002 (3)
C84	0.027 (3)	0.028 (3)	0.033 (3)	0.002 (3)	0.010 (3)	−0.003 (3)
C85	0.035 (4)	0.035 (3)	0.024 (3)	0.022 (3)	0.001 (3)	−0.008 (3)
C86	0.032 (3)	0.032 (3)	0.031 (3)	0.011 (3)	0.010 (3)	−0.004 (3)
C87	0.043 (4)	0.035 (4)	0.044 (4)	0.002 (3)	0.018 (3)	−0.014 (3)
N91	0.024 (3)	0.026 (3)	0.028 (3)	0.002 (2)	0.005 (2)	0.006 (2)
C92	0.029 (3)	0.038 (4)	0.024 (3)	0.009 (3)	0.004 (3)	0.002 (3)
C93	0.023 (3)	0.032 (3)	0.023 (3)	0.006 (2)	0.000 (2)	0.005 (2)
C94	0.023 (3)	0.021 (3)	0.030 (3)	0.001 (2)	0.000 (2)	0.006 (2)
C95	0.022 (3)	0.030 (3)	0.019 (3)	0.005 (2)	−0.004 (2)	0.002 (2)
C96	0.029 (3)	0.029 (3)	0.024 (3)	0.010 (3)	0.000 (2)	0.007 (2)
C97	0.034 (4)	0.027 (3)	0.045 (4)	0.006 (3)	−0.003 (3)	−0.003 (3)
N101	0.025 (3)	0.026 (3)	0.022 (3)	0.006 (2)	0.006 (2)	0.001 (2)
C102	0.026 (3)	0.021 (3)	0.028 (3)	0.006 (2)	0.004 (2)	−0.001 (2)
C103	0.027 (3)	0.025 (3)	0.027 (3)	0.005 (2)	0.001 (2)	0.000 (2)
C104	0.019 (3)	0.034 (3)	0.026 (3)	0.007 (2)	0.003 (2)	−0.009 (3)
C105	0.027 (3)	0.029 (3)	0.035 (4)	0.003 (3)	0.009 (3)	−0.007 (3)
C106	0.034 (3)	0.027 (3)	0.027 (3)	0.003 (3)	0.013 (3)	−0.002 (3)
C107	0.032 (4)	0.048 (4)	0.023 (3)	0.014 (3)	−0.003 (3)	−0.006 (3)

### Bis(4-methylpiperidinium) tetrachloridoaurate(III) chloride (2c) Geometric parameters (Å, º)

**Table:**

Au1—Cl3	2.2671 (13)	C54—C57	1.523 (8)
Au1—Cl4	2.2749 (13)	C54—H54	1.0000
Au1—Cl1	2.2750 (13)	C55—C56	1.515 (8)
Au1—Cl2	2.2953 (13)	C55—H55A	0.9900
Au2—Cl7	2.2667 (13)	C55—H55B	0.9900
Au2—Cl5	2.2792 (13)	C56—H56A	0.9900
Au2—Cl8	2.2872 (13)	C56—H56B	0.9900
Au2—Cl6	2.2902 (13)	C57—H57A	0.9800
Au3—Cl9	2.2624 (13)	C57—H57B	0.9800
Au3—Cl11	2.2698 (13)	C57—H57C	0.9800
Au3—Cl10	2.2747 (13)	N61—C66	1.475 (9)
Au3—Cl12	2.2875 (14)	N61—C62	1.483 (10)
Au4—Cl13	2.2816 (13)	N61—H61B	0.9100
Au4—Cl15	2.2962 (13)	N61—H61A	0.9100
Au4—Cl14	2.2983 (14)	C62—C63	1.531 (10)
Au4—Cl16	2.2985 (13)	C62—H62A	0.9900
Au5—Cl17	2.2788 (14)	C62—H62B	0.9900
Au5—Cl20	2.2794 (13)	C63—C64	1.507 (9)
Au5—Cl18	2.2795 (14)	C63—H63A	0.9900
Au5—Cl19	2.2969 (14)	C63—H63B	0.9900
N11—C12	1.495 (7)	C64—C65	1.513 (9)
N11—C16	1.498 (7)	C64—C67	1.525 (8)
N11—H01	0.90 (2)	C64—H64	1.0000
N11—H02	0.89 (2)	C65—C66	1.518 (9)
C12—C13	1.513 (7)	C65—H65A	0.9900
C12—H12A	0.9900	C65—H65B	0.9900
C12—H12B	0.9900	C66—H66A	0.9900
C13—C14	1.534 (7)	C66—H66B	0.9900
C13—H13A	0.9900	C67—H67A	0.9800
C13—H13B	0.9900	C67—H67B	0.9800
C14—C15	1.518 (8)	C67—H67C	0.9800
C14—C17	1.519 (8)	N71—C76	1.479 (8)
C14—H14	1.0000	N71—C72	1.488 (8)
C15—C16	1.512 (8)	N71—H71A	0.9100
C15—H15A	0.9900	N71—H71B	0.9100
C15—H15B	0.9900	C72—C73	1.515 (8)
C16—H16A	0.9900	C72—H72A	0.9900
C16—H16B	0.9900	C72—H72B	0.9900
C17—H17A	0.9800	C73—C74	1.516 (9)
C17—H17B	0.9800	C73—H73A	0.9900
C17—H17C	0.9800	C73—H73B	0.9900
N21—C26	1.490 (7)	C74—C77	1.510 (8)
N21—C22	1.498 (7)	C74—C75	1.542 (8)
N21—H03	0.90 (2)	C74—H74	1.0000
N21—H04	0.90 (2)	C75—C76	1.500 (8)
C22—C23	1.521 (8)	C75—H75A	0.9900
C22—H22A	0.9900	C75—H75B	0.9900
C22—H22B	0.9900	C76—H76A	0.9900
C23—C24	1.526 (7)	C76—H76B	0.9900
C23—H23A	0.9900	C77—H77A	0.9800
C23—H23B	0.9900	C77—H77B	0.9800
C24—C25	1.514 (7)	C77—H77C	0.9800
C24—C27	1.519 (7)	N81—C82	1.471 (10)
C24—H24	1.0000	N81—C86	1.490 (8)
C25—C26	1.509 (7)	N81—H81B	0.9100
C25—H25A	0.9900	N81—H81A	0.9100
C25—H25B	0.9900	C82—C83	1.491 (10)
C26—H26A	0.9900	C82—H82A	0.9900
C26—H26B	0.9900	C82—H82B	0.9900
C27—H27A	0.9800	C83—C84	1.514 (9)
C27—H27B	0.9800	C83—H83A	0.9900
C27—H27C	0.9800	C83—H83B	0.9900
N31—C32	1.467 (8)	C84—C85	1.509 (8)
N31—C36	1.481 (9)	C84—C87	1.517 (8)
N31—H05	0.90 (2)	C84—H84	1.0000
N31—H06	0.90 (2)	C85—C86	1.518 (7)
C32—C33	1.515 (8)	C85—H85A	0.9900
C32—H32A	0.9900	C85—H85B	0.9900
C32—H32B	0.9900	C86—H86A	0.9900
C33—C34	1.513 (8)	C86—H86B	0.9900
C33—H33A	0.9900	C87—H87A	0.9800
C33—H33B	0.9900	C87—H87B	0.9800
C34—C37	1.524 (8)	C87—H87C	0.9800
C34—C35	1.527 (8)	N91—C96	1.491 (8)
C34—H34	1.0000	N91—C92	1.495 (8)
C35—C36	1.518 (8)	N91—H017	0.93 (3)
C35—H35A	0.9900	N91—H018	0.93 (3)
C35—H35B	0.9900	C92—C93	1.503 (8)
C36—H36A	0.9900	C92—H92A	0.9900
C36—H36B	0.9900	C92—H92B	0.9900
C37—H37A	0.9800	C93—C94	1.514 (8)
C37—H37B	0.9800	C93—H93A	0.9900
C37—H37C	0.9800	C93—H93B	0.9900
N41—C46	1.492 (7)	C94—C97	1.513 (8)
N41—C42	1.500 (7)	C94—C95	1.535 (8)
N41—H07	0.89 (2)	C94—H94	1.0000
N41—H08	0.89 (2)	C95—C96	1.507 (8)
C42—C43	1.518 (8)	C95—H95A	0.9900
C42—H42A	0.9900	C95—H95B	0.9900
C42—H42B	0.9900	C96—H96A	0.9900
C43—C44	1.530 (8)	C96—H96B	0.9900
C43—H43A	0.9900	C97—H97A	0.9800
C43—H43B	0.9900	C97—H97B	0.9800
C44—C47	1.519 (8)	C97—H97C	0.9800
C44—C45	1.529 (8)	N101—C106	1.485 (8)
C44—H44	1.0000	N101—C102	1.486 (7)
C45—C46	1.511 (8)	N101—H019	0.93 (3)
C45—H45A	0.9900	N101—H020	0.93 (3)
C45—H45B	0.9900	C102—C103	1.531 (8)
C46—H46A	0.9900	C102—H10A	0.9900
C46—H46B	0.9900	C102—H10B	0.9900
C47—H47A	0.9800	C103—C104	1.530 (8)
C47—H47B	0.9800	C103—H10C	0.9900
C47—H47C	0.9800	C103—H10D	0.9900
N51—C52	1.481 (8)	C104—C107	1.510 (8)
N51—C56	1.489 (8)	C104—C105	1.538 (9)
N51—H09	0.93 (3)	C104—H10E	1.0000
N51—H010	0.93 (3)	C105—C106	1.517 (8)
C52—C53	1.515 (8)	C105—H10F	0.9900
C52—H52A	0.9900	C105—H10G	0.9900
C52—H52B	0.9900	C106—H10H	0.9900
C53—C54	1.533 (8)	C106—H10I	0.9900
C53—H53A	0.9900	C107—H10J	0.9800
C53—H53B	0.9900	C107—H10K	0.9800
C54—C55	1.513 (8)	C107—H10L	0.9800
			
Cl3—Au1—Cl4	89.77 (5)	C54—C55—H55B	109.0
Cl3—Au1—Cl1	178.58 (6)	C56—C55—H55B	109.0
Cl4—Au1—Cl1	89.91 (5)	H55A—C55—H55B	107.8
Cl3—Au1—Cl2	90.35 (5)	N51—C56—C55	110.5 (5)
Cl4—Au1—Cl2	178.72 (5)	N51—C56—H56A	109.6
Cl1—Au1—Cl2	89.99 (5)	C55—C56—H56A	109.6
Cl7—Au2—Cl5	178.76 (6)	N51—C56—H56B	109.6
Cl7—Au2—Cl8	91.29 (5)	C55—C56—H56B	109.6
Cl5—Au2—Cl8	89.45 (5)	H56A—C56—H56B	108.1
Cl7—Au2—Cl6	89.04 (5)	C54—C57—H57A	109.5
Cl5—Au2—Cl6	90.24 (5)	C54—C57—H57B	109.5
Cl8—Au2—Cl6	178.58 (5)	H57A—C57—H57B	109.5
Cl9—Au3—Cl11	177.25 (6)	C54—C57—H57C	109.5
Cl9—Au3—Cl10	89.01 (5)	H57A—C57—H57C	109.5
Cl11—Au3—Cl10	90.97 (5)	H57B—C57—H57C	109.5
Cl9—Au3—Cl12	90.95 (5)	C66—N61—C62	113.2 (5)
Cl11—Au3—Cl12	89.13 (5)	C66—N61—H61B	108.9
Cl10—Au3—Cl12	178.86 (6)	C62—N61—H61B	108.9
Cl13—Au4—Cl15	179.66 (5)	C66—N61—H61A	108.9
Cl13—Au4—Cl14	89.19 (5)	C62—N61—H61A	108.9
Cl15—Au4—Cl14	90.76 (5)	H61B—N61—H61A	107.7
Cl13—Au4—Cl16	89.79 (5)	N61—C62—C63	110.5 (7)
Cl15—Au4—Cl16	90.25 (5)	N61—C62—H62A	109.6
Cl14—Au4—Cl16	178.41 (5)	C63—C62—H62A	109.6
Cl17—Au5—Cl20	90.16 (5)	N61—C62—H62B	109.6
Cl17—Au5—Cl18	89.47 (5)	C63—C62—H62B	109.6
Cl20—Au5—Cl18	179.45 (6)	H62A—C62—H62B	108.1
Cl17—Au5—Cl19	179.24 (5)	C64—C63—C62	112.0 (5)
Cl20—Au5—Cl19	89.98 (5)	C64—C63—H63A	109.2
Cl18—Au5—Cl19	90.40 (5)	C62—C63—H63A	109.2
C12—N11—C16	113.3 (4)	C64—C63—H63B	109.2
C12—N11—H01	103 (4)	C62—C63—H63B	109.2
C16—N11—H01	114 (4)	H63A—C63—H63B	107.9
C12—N11—H02	109 (5)	C63—C64—C65	110.4 (6)
C16—N11—H02	112 (5)	C63—C64—C67	112.2 (5)
H01—N11—H02	105 (4)	C65—C64—C67	110.1 (6)
N11—C12—C13	110.8 (4)	C63—C64—H64	108.0
N11—C12—H12A	109.5	C65—C64—H64	108.0
C13—C12—H12A	109.5	C67—C64—H64	108.0
N11—C12—H12B	109.5	C64—C65—C66	112.8 (6)
C13—C12—H12B	109.5	C64—C65—H65A	109.0
H12A—C12—H12B	108.1	C66—C65—H65A	109.0
C12—C13—C14	111.6 (4)	C64—C65—H65B	109.0
C12—C13—H13A	109.3	C66—C65—H65B	109.0
C14—C13—H13A	109.3	H65A—C65—H65B	107.8
C12—C13—H13B	109.3	N61—C66—C65	109.1 (6)
C14—C13—H13B	109.3	N61—C66—H66A	109.9
H13A—C13—H13B	108.0	C65—C66—H66A	109.9
C15—C14—C17	112.3 (5)	N61—C66—H66B	109.9
C15—C14—C13	108.9 (5)	C65—C66—H66B	109.9
C17—C14—C13	111.7 (5)	H66A—C66—H66B	108.3
C15—C14—H14	108.0	C64—C67—H67A	109.5
C17—C14—H14	108.0	C64—C67—H67B	109.5
C13—C14—H14	108.0	H67A—C67—H67B	109.5
C16—C15—C14	111.9 (5)	C64—C67—H67C	109.5
C16—C15—H15A	109.2	H67A—C67—H67C	109.5
C14—C15—H15A	109.2	H67B—C67—H67C	109.5
C16—C15—H15B	109.2	C76—N71—C72	114.5 (5)
C14—C15—H15B	109.2	C76—N71—H71A	108.6
H15A—C15—H15B	107.9	C72—N71—H71A	108.6
N11—C16—C15	109.8 (4)	C76—N71—H71B	108.6
N11—C16—H16A	109.7	C72—N71—H71B	108.6
C15—C16—H16A	109.7	H71A—N71—H71B	107.6
N11—C16—H16B	109.7	N71—C72—C73	110.5 (5)
C15—C16—H16B	109.7	N71—C72—H72A	109.6
H16A—C16—H16B	108.2	C73—C72—H72A	109.6
C14—C17—H17A	109.5	N71—C72—H72B	109.6
C14—C17—H17B	109.5	C73—C72—H72B	109.6
H17A—C17—H17B	109.5	H72A—C72—H72B	108.1
C14—C17—H17C	109.5	C72—C73—C74	112.6 (5)
H17A—C17—H17C	109.5	C72—C73—H73A	109.1
H17B—C17—H17C	109.5	C74—C73—H73A	109.1
C26—N21—C22	112.0 (4)	C72—C73—H73B	109.1
C26—N21—H03	111 (3)	C74—C73—H73B	109.1
C22—N21—H03	107 (3)	H73A—C73—H73B	107.8
C26—N21—H04	113 (3)	C77—C74—C73	111.3 (5)
C22—N21—H04	110 (3)	C77—C74—C75	111.8 (6)
H03—N21—H04	103 (3)	C73—C74—C75	109.3 (5)
N21—C22—C23	109.1 (4)	C77—C74—H74	108.1
N21—C22—H22A	109.9	C73—C74—H74	108.1
C23—C22—H22A	109.9	C75—C74—H74	108.1
N21—C22—H22B	109.9	C76—C75—C74	111.6 (5)
C23—C22—H22B	109.9	C76—C75—H75A	109.3
H22A—C22—H22B	108.3	C74—C75—H75A	109.3
C22—C23—C24	111.0 (5)	C76—C75—H75B	109.3
C22—C23—H23A	109.4	C74—C75—H75B	109.3
C24—C23—H23A	109.4	H75A—C75—H75B	108.0
C22—C23—H23B	109.4	N71—C76—C75	111.6 (5)
C24—C23—H23B	109.4	N71—C76—H76A	109.3
H23A—C23—H23B	108.0	C75—C76—H76A	109.3
C25—C24—C27	112.4 (5)	N71—C76—H76B	109.3
C25—C24—C23	109.3 (5)	C75—C76—H76B	109.3
C27—C24—C23	109.9 (5)	H76A—C76—H76B	108.0
C25—C24—H24	108.4	C74—C77—H77A	109.5
C27—C24—H24	108.4	C74—C77—H77B	109.5
C23—C24—H24	108.4	H77A—C77—H77B	109.5
C26—C25—C24	112.6 (4)	C74—C77—H77C	109.5
C26—C25—H25A	109.1	H77A—C77—H77C	109.5
C24—C25—H25A	109.1	H77B—C77—H77C	109.5
C26—C25—H25B	109.1	C82—N81—C86	113.3 (5)
C24—C25—H25B	109.1	C82—N81—H81B	108.9
H25A—C25—H25B	107.8	C86—N81—H81B	108.9
N21—C26—C25	109.9 (4)	C82—N81—H81A	108.9
N21—C26—H26A	109.7	C86—N81—H81A	108.9
C25—C26—H26A	109.7	H81B—N81—H81A	107.7
N21—C26—H26B	109.7	N81—C82—C83	111.1 (5)
C25—C26—H26B	109.7	N81—C82—H82A	109.4
H26A—C26—H26B	108.2	C83—C82—H82A	109.4
C24—C27—H27A	109.5	N81—C82—H82B	109.4
C24—C27—H27B	109.5	C83—C82—H82B	109.4
H27A—C27—H27B	109.5	H82A—C82—H82B	108.0
C24—C27—H27C	109.5	C82—C83—C84	111.7 (6)
H27A—C27—H27C	109.5	C82—C83—H83A	109.3
H27B—C27—H27C	109.5	C84—C83—H83A	109.3
C32—N31—C36	114.6 (5)	C82—C83—H83B	109.3
C32—N31—H05	111 (5)	C84—C83—H83B	109.3
C36—N31—H05	111 (5)	H83A—C83—H83B	107.9
C32—N31—H06	109 (5)	C85—C84—C83	108.2 (5)
C36—N31—H06	106 (5)	C85—C84—C87	112.1 (5)
H05—N31—H06	104 (4)	C83—C84—C87	112.1 (6)
N31—C32—C33	110.7 (5)	C85—C84—H84	108.1
N31—C32—H32A	109.5	C83—C84—H84	108.1
C33—C32—H32A	109.5	C87—C84—H84	108.1
N31—C32—H32B	109.5	C84—C85—C86	111.9 (5)
C33—C32—H32B	109.5	C84—C85—H85A	109.2
H32A—C32—H32B	108.1	C86—C85—H85A	109.2
C34—C33—C32	112.4 (5)	C84—C85—H85B	109.2
C34—C33—H33A	109.1	C86—C85—H85B	109.2
C32—C33—H33A	109.1	H85A—C85—H85B	107.9
C34—C33—H33B	109.1	N81—C86—C85	109.5 (5)
C32—C33—H33B	109.1	N81—C86—H86A	109.8
H33A—C33—H33B	107.9	C85—C86—H86A	109.8
C33—C34—C37	112.3 (5)	N81—C86—H86B	109.8
C33—C34—C35	109.4 (5)	C85—C86—H86B	109.8
C37—C34—C35	111.4 (5)	H86A—C86—H86B	108.2
C33—C34—H34	107.8	C84—C87—H87A	109.5
C37—C34—H34	107.8	C84—C87—H87B	109.5
C35—C34—H34	107.8	H87A—C87—H87B	109.5
C36—C35—C34	111.5 (5)	C84—C87—H87C	109.5
C36—C35—H35A	109.3	H87A—C87—H87C	109.5
C34—C35—H35A	109.3	H87B—C87—H87C	109.5
C36—C35—H35B	109.3	C96—N91—C92	112.4 (5)
C34—C35—H35B	109.3	C96—N91—H017	111 (6)
H35A—C35—H35B	108.0	C92—N91—H017	112 (6)
N31—C36—C35	111.1 (5)	C96—N91—H018	107 (5)
N31—C36—H36A	109.4	C92—N91—H018	111 (5)
C35—C36—H36A	109.4	H017—N91—H018	103 (4)
N31—C36—H36B	109.4	N91—C92—C93	110.0 (5)
C35—C36—H36B	109.4	N91—C92—H92A	109.7
H36A—C36—H36B	108.0	C93—C92—H92A	109.7
C34—C37—H37A	109.5	N91—C92—H92B	109.7
C34—C37—H37B	109.5	C93—C92—H92B	109.7
H37A—C37—H37B	109.5	H92A—C92—H92B	108.2
C34—C37—H37C	109.5	C92—C93—C94	113.0 (5)
H37A—C37—H37C	109.5	C92—C93—H93A	109.0
H37B—C37—H37C	109.5	C94—C93—H93A	109.0
C46—N41—C42	111.8 (5)	C92—C93—H93B	109.0
C46—N41—H07	110 (4)	C94—C93—H93B	109.0
C42—N41—H07	110 (4)	H93A—C93—H93B	107.8
C46—N41—H08	115 (5)	C97—C94—C93	112.5 (5)
C42—N41—H08	102 (5)	C97—C94—C95	111.1 (5)
H07—N41—H08	108 (4)	C93—C94—C95	108.4 (5)
N41—C42—C43	110.2 (5)	C97—C94—H94	108.3
N41—C42—H42A	109.6	C93—C94—H94	108.3
C43—C42—H42A	109.6	C95—C94—H94	108.3
N41—C42—H42B	109.6	C96—C95—C94	113.2 (5)
C43—C42—H42B	109.6	C96—C95—H95A	108.9
H42A—C42—H42B	108.1	C94—C95—H95A	108.9
C42—C43—C44	111.7 (5)	C96—C95—H95B	108.9
C42—C43—H43A	109.3	C94—C95—H95B	108.9
C44—C43—H43A	109.3	H95A—C95—H95B	107.8
C42—C43—H43B	109.3	N91—C96—C95	110.7 (5)
C44—C43—H43B	109.3	N91—C96—H96A	109.5
H43A—C43—H43B	107.9	C95—C96—H96A	109.5
C47—C44—C45	111.3 (5)	N91—C96—H96B	109.5
C47—C44—C43	111.6 (5)	C95—C96—H96B	109.5
C45—C44—C43	109.3 (5)	H96A—C96—H96B	108.1
C47—C44—H44	108.2	C94—C97—H97A	109.5
C45—C44—H44	108.2	C94—C97—H97B	109.5
C43—C44—H44	108.2	H97A—C97—H97B	109.5
C46—C45—C44	111.2 (5)	C94—C97—H97C	109.5
C46—C45—H45A	109.4	H97A—C97—H97C	109.5
C44—C45—H45A	109.4	H97B—C97—H97C	109.5
C46—C45—H45B	109.4	C106—N101—C102	112.3 (5)
C44—C45—H45B	109.4	C106—N101—H019	107 (5)
H45A—C45—H45B	108.0	C102—N101—H019	117 (5)
N41—C46—C45	110.8 (4)	C106—N101—H020	112 (4)
N41—C46—H46A	109.5	C102—N101—H020	108 (3)
C45—C46—H46A	109.5	H019—N101—H020	101 (4)
N41—C46—H46B	109.5	N101—C102—C103	110.0 (5)
C45—C46—H46B	109.5	N101—C102—H10A	109.7
H46A—C46—H46B	108.1	C103—C102—H10A	109.7
C44—C47—H47A	109.5	N101—C102—H10B	109.7
C44—C47—H47B	109.5	C103—C102—H10B	109.7
H47A—C47—H47B	109.5	H10A—C102—H10B	108.2
C44—C47—H47C	109.5	C104—C103—C102	112.8 (5)
H47A—C47—H47C	109.5	C104—C103—H10C	109.0
H47B—C47—H47C	109.5	C102—C103—H10C	109.0
C52—N51—C56	113.1 (5)	C104—C103—H10D	109.0
C52—N51—H09	117 (4)	C102—C103—H10D	109.0
C56—N51—H09	104 (4)	H10C—C103—H10D	107.8
C52—N51—H010	116 (5)	C107—C104—C103	110.9 (5)
C56—N51—H010	103 (5)	C107—C104—C105	110.9 (5)
H09—N51—H010	101 (4)	C103—C104—C105	109.8 (5)
N51—C52—C53	110.3 (5)	C107—C104—H10E	108.4
N51—C52—H52A	109.6	C103—C104—H10E	108.4
C53—C52—H52A	109.6	C105—C104—H10E	108.4
N51—C52—H52B	109.6	C106—C105—C104	112.3 (5)
C53—C52—H52B	109.6	C106—C105—H10F	109.2
H52A—C52—H52B	108.1	C104—C105—H10F	109.2
C52—C53—C54	112.5 (5)	C106—C105—H10G	109.2
C52—C53—H53A	109.1	C104—C105—H10G	109.2
C54—C53—H53A	109.1	H10F—C105—H10G	107.9
C52—C53—H53B	109.1	N101—C106—C105	109.8 (5)
C54—C53—H53B	109.1	N101—C106—H10H	109.7
H53A—C53—H53B	107.8	C105—C106—H10H	109.7
C55—C54—C57	111.6 (5)	N101—C106—H10I	109.7
C55—C54—C53	109.5 (5)	C105—C106—H10I	109.7
C57—C54—C53	110.9 (5)	H10H—C106—H10I	108.2
C55—C54—H54	108.3	C104—C107—H10J	109.5
C57—C54—H54	108.3	C104—C107—H10K	109.5
C53—C54—H54	108.3	H10J—C107—H10K	109.5
C54—C55—C56	112.7 (5)	C104—C107—H10L	109.5
C54—C55—H55A	109.0	H10J—C107—H10L	109.5
C56—C55—H55A	109.0	H10K—C107—H10L	109.5
			
C16—N11—C12—C13	54.6 (6)	C66—N61—C62—C63	56.9 (8)
N11—C12—C13—C14	−54.7 (6)	N61—C62—C63—C64	−53.4 (9)
C12—C13—C14—C15	56.1 (6)	C62—C63—C64—C65	52.1 (9)
C12—C13—C14—C17	−179.3 (5)	C62—C63—C64—C67	175.4 (7)
C17—C14—C15—C16	178.2 (5)	C63—C64—C65—C66	−54.1 (8)
C13—C14—C15—C16	−57.6 (6)	C67—C64—C65—C66	−178.5 (6)
C12—N11—C16—C15	−55.4 (6)	C62—N61—C66—C65	−57.8 (7)
C14—C15—C16—N11	57.1 (6)	C64—C65—C66—N61	56.1 (8)
C26—N21—C22—C23	−59.1 (6)	C76—N71—C72—C73	−52.8 (7)
N21—C22—C23—C24	58.2 (6)	N71—C72—C73—C74	54.0 (8)
C22—C23—C24—C25	−56.2 (6)	C72—C73—C74—C77	−179.3 (6)
C22—C23—C24—C27	180.0 (5)	C72—C73—C74—C75	−55.3 (7)
C27—C24—C25—C26	177.4 (5)	C77—C74—C75—C76	178.6 (5)
C23—C24—C25—C26	55.1 (6)	C73—C74—C75—C76	54.9 (7)
C22—N21—C26—C25	57.5 (6)	C72—N71—C76—C75	53.6 (7)
C24—C25—C26—N21	−55.6 (6)	C74—C75—C76—N71	−54.0 (7)
C36—N31—C32—C33	53.5 (6)	C86—N81—C82—C83	55.1 (7)
N31—C32—C33—C34	−54.5 (7)	N81—C82—C83—C84	−56.5 (8)
C32—C33—C34—C37	179.9 (5)	C82—C83—C84—C85	57.3 (7)
C32—C33—C34—C35	55.6 (6)	C82—C83—C84—C87	−178.5 (6)
C33—C34—C35—C36	−55.0 (6)	C83—C84—C85—C86	−57.5 (7)
C37—C34—C35—C36	−179.8 (5)	C87—C84—C85—C86	178.3 (5)
C32—N31—C36—C35	−53.6 (7)	C82—N81—C86—C85	−54.3 (7)
C34—C35—C36—N31	53.7 (7)	C84—C85—C86—N81	56.0 (7)
C46—N41—C42—C43	−56.7 (7)	C96—N91—C92—C93	56.7 (6)
N41—C42—C43—C44	56.1 (7)	N91—C92—C93—C94	−57.3 (7)
C42—C43—C44—C47	−179.1 (5)	C92—C93—C94—C97	178.1 (5)
C42—C43—C44—C45	−55.6 (7)	C92—C93—C94—C95	54.9 (6)
C47—C44—C45—C46	179.4 (5)	C97—C94—C95—C96	−177.3 (5)
C43—C44—C45—C46	55.7 (6)	C93—C94—C95—C96	−53.2 (6)
C42—N41—C46—C45	57.5 (7)	C92—N91—C96—C95	−55.3 (6)
C44—C45—C46—N41	−57.1 (6)	C94—C95—C96—N91	54.1 (6)
C56—N51—C52—C53	56.3 (6)	C106—N101—C102—C103	58.3 (6)
N51—C52—C53—C54	−55.0 (6)	N101—C102—C103—C104	−54.3 (6)
C52—C53—C54—C55	53.5 (6)	C102—C103—C104—C107	174.2 (5)
C52—C53—C54—C57	177.1 (5)	C102—C103—C104—C105	51.4 (6)
C57—C54—C55—C56	−176.5 (5)	C107—C104—C105—C106	−175.4 (5)
C53—C54—C55—C56	−53.3 (6)	C103—C104—C105—C106	−52.6 (6)
C52—N51—C56—C55	−56.2 (6)	C102—N101—C106—C105	−59.7 (6)
C54—C55—C56—N51	54.8 (6)	C104—C105—C106—N101	56.6 (7)

### Bis(4-methylpiperidinium) tetrachloridoaurate(III) chloride (2c) Hydrogen-bond geometry (Å, º)

**Table:**

*D*—H···*A*	*D*—H	H···*A*	*D*···*A*	*D*—H···*A*
N11—H01···Cl21	0.90 (2)	2.18 (3)	3.073 (5)	171 (5)
N11—H02···Cl6^i^	0.89 (2)	2.60 (5)	3.335 (5)	140 (6)
N21—H03···Cl21	0.90 (2)	2.30 (2)	3.182 (5)	167 (4)
N21—H04···Cl22	0.90 (2)	2.50 (4)	3.252 (5)	142 (4)
N31—H05···Cl21	0.90 (2)	2.22 (3)	3.099 (5)	167 (6)
N31—H06···Cl22	0.90 (2)	2.27 (3)	3.134 (5)	162 (7)
N41—H07···Cl23	0.89 (2)	2.32 (2)	3.196 (5)	171 (5)
N41—H08···Cl22	0.89 (2)	2.59 (6)	3.224 (5)	129 (6)
N51—H09···Cl22	0.93 (3)	2.33 (4)	3.180 (5)	151 (6)
N51—H010···Cl23	0.93 (3)	2.22 (3)	3.126 (5)	166 (6)
N61—H61*B*···Cl23	0.91	2.28	3.161 (6)	164
N61—H61*A*···Cl24	0.91	2.57	3.325 (5)	141
N71—H71*A*···Cl23	0.91	2.32	3.216 (5)	169
N71—H71*B*···Cl24	0.91	2.57	3.254 (5)	132
N81—H81*B*···Cl25	0.91	2.26	3.157 (5)	170
N81—H81*A*···Cl24	0.91	2.24	3.146 (5)	175
N91—H017···Cl24	0.93 (3)	2.56 (6)	3.356 (5)	144 (7)
N91—H018···Cl25	0.93 (3)	2.23 (3)	3.134 (5)	163 (7)
N101—H019···Cl5	0.93 (3)	2.64 (4)	3.459 (5)	147 (6)
N101—H019···Cl8	0.93 (3)	2.72 (5)	3.471 (5)	138 (6)
N101—H020···Cl25	0.93 (3)	2.15 (3)	3.075 (5)	170 (4)
C12—H12*A*···Cl1^ii^	0.99	2.81	3.753 (6)	159
C12—H12*A*···Cl5^i^	0.99	2.89	3.553 (6)	125
C16—H16*A*···Cl13^iii^	0.99	2.95	3.886 (6)	157
C22—H22*A*···Cl20	0.99	2.93	3.700 (6)	135
C26—H26*A*···Cl6^iv^	0.99	2.95	3.826 (6)	148
C26—H26*B*···Cl21^iii^	0.99	2.89	3.570 (6)	127
C36—H36*A*···Cl13	0.99	2.92	3.847 (6)	157
C42—H42*B*···Cl7^iv^	0.99	2.78	3.744 (7)	164
C43—H43*A*···Cl10	0.99	2.93	3.785 (7)	145
C45—H45*A*···Cl4^v^	0.99	2.85	3.629 (6)	136
C46—H46*B*···Cl18	0.99	2.95	3.766 (7)	140
C52—H52*A*···Cl2	0.99	2.92	3.863 (6)	159
C52—H52*B*···Cl17	0.99	2.90	3.496 (6)	119
C53—H53*B*···Cl17	0.99	2.84	3.554 (6)	129
C56—H56*A*···Cl15	0.99	2.78	3.692 (6)	154
C65—H65*B*···Cl3	0.99	2.87	3.581 (7)	130
C66—H66*A*···Cl3	0.99	2.97	3.569 (7)	120
C66—H66*B*···Cl15	0.99	2.97	3.923 (7)	162
C75—H75*A*···Cl11^vi^	0.99	2.97	3.724 (6)	134
C76—H76*B*···Cl10	0.99	2.75	3.638 (7)	149
C76—H76*B*···Cl24	0.99	2.91	3.389 (6)	111
C82—H82*B*···Cl12	0.99	2.66	3.629 (7)	166
C83—H83*A*···Cl9^iv^	0.99	2.96	3.664 (7)	129
C86—H86*B*···Cl7^vii^	0.99	2.95	3.511 (6)	117
C92—H92*B*···Cl12	0.99	2.97	3.740 (7)	135
C93—H93*B*···Cl11	0.99	2.79	3.515 (6)	131
C93—H93*B*···Cl11^vi^	0.99	2.97	3.635 (6)	125
C96—H96*A*···Cl4	0.99	2.81	3.773 (6)	163
C96—H96*B*···Cl25^viii^	0.99	2.78	3.676 (6)	151
C102—H10*B*···Cl4^viii^	0.99	2.98	3.716 (6)	132
C103—H10*C*···Cl5	0.99	2.92	3.736 (6)	141
C105—H10*G*···Cl15^iv^	0.99	2.90	3.608 (6)	129
C106—H10*I*···Cl12	0.99	2.95	3.877 (6)	157

Symmetry codes: (i) *x*+1, *y*, *z*−1; (ii) −*x*+1, −*y*+1, −*z*; (iii) −*x*+2, −*y*+2, −*z*; (iv) −*x*+1, −*y*+2, −*z*+1; (v) *x*+1, *y*, *z*; (vi) −*x*+1, −*y*+1, −*z*+1; (vii) −*x*, −*y*+2, −*z*+1; (viii) −*x*, −*y*+1, −*z*+1.

### Hexakis(4-methylpiperidinium) tetrakis{tetrachloridoaurate(III)} dichloroiodate(I) chloride (3) Crystal data

**Table:**

(C_6_H_14_N)[AuCl_4_]_4_(Cl_2_I)Cl	*Z* = 1
*M**_r_* = 2189.40	*F*(000) = 1034
Triclinic, *P*1	*D*_x_ = 2.241 Mg m^−^^3^
*a* = 9.5362 (5) Å	Mo *K*α radiation, λ = 0.71073 Å
*b* = 13.4772 (6) Å	Cell parameters from 17356 reflections
*c* = 13.7179 (7) Å	θ = 2.4–29.8°
α = 98.422 (4)°	µ = 10.31 mm^−^^1^
β = 108.961 (5)°	*T* = 100 K
γ = 96.954 (4)°	Irregular, orange
*V* = 1622.36 (15) Å^3^	0.1 × 0.1 × 0.04 mm

### Hexakis(4-methylpiperidinium) tetrakis{tetrachloridoaurate(III)} dichloroiodate(I) chloride (3) Data collection

**Table:**

Oxford Diffraction Xcalibur, Eos diffractometer	9693 independent reflections
Radiation source: Enhance (Mo) X-ray Source	8260 reflections with *I* > 2σ(*I*)
Detector resolution: 16.1419 pixels mm^-1^	*R*_int_ = 0.072
ω scan	θ_max_ = 30.9°, θ_min_ = 2.3°
Absorption correction: multi-scan (CrysAlisPro; Rigaku OD, 2013)	*h* = −13→13
*T*_min_ = 0.667, *T*_max_ = 1.000	*k* = −19→19
97738 measured reflections	*l* = −19→19

### Hexakis(4-methylpiperidinium) tetrakis{tetrachloridoaurate(III)} dichloroiodate(I) chloride (3) Refinement

**Table:**

Refinement on *F*^2^	Primary atom site location: structure-invariant direct methods
Least-squares matrix: full	Secondary atom site location: difference Fourier map
*R*[*F*^2^ > 2σ(*F*^2^)] = 0.029	Hydrogen site location: mixed
*wR*(*F*^2^) = 0.046	H atoms treated by a mixture of independent and constrained refinement
*S* = 1.05	*w* = 1/[σ^2^(*F*_o_^2^) + (0.0126*P*)^2^ + 0.4353*P*] where *P* = (*F*_o_^2^ + 2*F*_c_^2^)/3
9693 reflections	(Δ/σ)_max_ = 0.003
331 parameters	Δρ_max_ = 1.16 e Å^−^^3^
18 restraints	Δρ_min_ = −0.96 e Å^−^^3^

### Hexakis(4-methylpiperidinium) tetrakis{tetrachloridoaurate(III)} dichloroiodate(I) chloride (3) Fractional atomic coordinates and isotropic or equivalent isotropic displacement parameters (Å^2^)

**Table:**

	*x*	*y*	*z*	*U*_iso_*/*U*_eq_	
Au1	0.39387 (2)	0.50585 (2)	0.20597 (2)	0.01070 (3)	
Au2	1.000000	0.500000	0.500000	0.01058 (4)	
Au3	0.500000	0.000000	0.000000	0.01265 (4)	
I1	1.000000	1.000000	0.500000	0.01432 (7)	
Cl1	0.15106 (9)	0.50346 (7)	0.10488 (7)	0.01718 (18)	
Cl2	0.44699 (10)	0.67948 (7)	0.22697 (7)	0.01853 (19)	
Cl3	0.63842 (9)	0.50623 (7)	0.30913 (7)	0.01897 (19)	
Cl4	0.34111 (10)	0.33372 (6)	0.19184 (7)	0.01687 (18)	
Cl5	1.01344 (10)	0.67022 (6)	0.50122 (7)	0.01606 (18)	
Cl6	1.13481 (9)	0.48389 (6)	0.38799 (7)	0.01554 (17)	
Cl7	0.37502 (11)	0.08877 (7)	0.08891 (8)	0.0229 (2)	
Cl8	0.49745 (10)	0.12140 (7)	−0.10024 (7)	0.01984 (19)	
Cl9	0.500000	0.500000	0.000000	0.0195 (3)	
Cl10	0.78118 (10)	0.86637 (7)	0.36866 (7)	0.02082 (19)	
N11	0.7720 (3)	0.6787 (2)	0.1638 (3)	0.0172 (7)	
H01	0.691 (3)	0.627 (2)	0.136 (3)	0.042 (14)*	
H02	0.754 (4)	0.722 (2)	0.214 (2)	0.013 (10)*	
C12	0.7924 (4)	0.7338 (3)	0.0815 (3)	0.0181 (8)	
H12A	0.699977	0.760642	0.048254	0.022*	
H12B	0.809901	0.686093	0.026238	0.022*	
C13	0.9255 (4)	0.8210 (3)	0.1300 (3)	0.0178 (8)	
H13A	0.903511	0.871319	0.180847	0.021*	
H13B	0.939825	0.855629	0.074170	0.021*	
C14	1.0709 (4)	0.7846 (3)	0.1864 (3)	0.0145 (7)	
H14	1.097838	0.739844	0.132814	0.017*	
C15	1.0452 (4)	0.7218 (3)	0.2651 (3)	0.0157 (7)	
H15A	1.135940	0.692069	0.295325	0.019*	
H15B	1.030850	0.767257	0.323339	0.019*	
C16	0.9097 (4)	0.6369 (3)	0.2156 (3)	0.0158 (8)	
H16A	0.928244	0.586828	0.162829	0.019*	
H16B	0.893617	0.601265	0.270326	0.019*	
C17	1.2006 (4)	0.8740 (3)	0.2411 (3)	0.0245 (9)	
H17A	1.212238	0.915099	0.189952	0.037*	
H17B	1.293868	0.848293	0.271288	0.037*	
H17C	1.179138	0.916204	0.297206	0.037*	
N21	0.6624 (3)	0.6990 (2)	0.4978 (2)	0.0146 (6)	
H03	0.724 (4)	0.653 (2)	0.497 (3)	0.021 (11)*	
H04	0.647 (4)	0.724 (3)	0.438 (2)	0.035 (13)*	
C22	0.7427 (4)	0.7843 (3)	0.5896 (3)	0.0174 (8)	
H22A	0.838990	0.814768	0.584169	0.021*	
H22B	0.765934	0.757579	0.655477	0.021*	
C23	0.6463 (4)	0.8648 (3)	0.5926 (3)	0.0180 (8)	
H23A	0.631318	0.895691	0.529586	0.022*	
H23B	0.699429	0.919297	0.655424	0.022*	
C24	0.4924 (4)	0.8207 (3)	0.5958 (3)	0.0163 (8)	
H24	0.509181	0.794555	0.662550	0.020*	
C25	0.4167 (4)	0.7309 (3)	0.5035 (3)	0.0193 (8)	
H25A	0.320703	0.698964	0.508315	0.023*	
H25B	0.392825	0.756364	0.436901	0.023*	
C26	0.5157 (4)	0.6512 (3)	0.5016 (3)	0.0188 (8)	
H26A	0.533235	0.621231	0.565349	0.023*	
H26B	0.464668	0.595859	0.439293	0.023*	
C27	0.3942 (5)	0.9023 (3)	0.5949 (3)	0.0279 (10)	
H27A	0.378652	0.930149	0.530897	0.042*	
H27B	0.444311	0.957106	0.656694	0.042*	
H27C	0.296460	0.871910	0.596628	0.042*	
N31	0.6699 (3)	0.3078 (3)	0.1185 (3)	0.0207 (7)	
H05	0.618 (4)	0.359 (2)	0.124 (3)	0.040 (13)*	
H06	0.607 (3)	0.2556 (19)	0.068 (2)	0.019 (11)*	
C32	0.7237 (4)	0.2736 (3)	0.2207 (3)	0.0253 (9)	
H32A	0.636631	0.251667	0.241597	0.030*	
H32B	0.791673	0.331070	0.275803	0.030*	
C33	0.8070 (4)	0.1859 (3)	0.2112 (3)	0.0177 (8)	
H33A	0.735482	0.126199	0.161688	0.021*	
H33B	0.846745	0.166763	0.280675	0.021*	
C34	0.9367 (4)	0.2125 (3)	0.1726 (3)	0.0159 (8)	
H34	1.015005	0.266442	0.227920	0.019*	
C35	0.8813 (4)	0.2549 (3)	0.0717 (3)	0.0192 (8)	
H35A	0.968933	0.279658	0.052571	0.023*	
H35B	0.814581	0.199223	0.013767	0.023*	
C36	0.7967 (4)	0.3407 (3)	0.0831 (3)	0.0235 (9)	
H36A	0.866135	0.399933	0.134887	0.028*	
H36B	0.756969	0.362096	0.014760	0.028*	
C37	1.0079 (4)	0.1189 (3)	0.1546 (3)	0.0257 (9)	
H37A	1.032042	0.088624	0.217545	0.039*	
H37B	1.100438	0.139281	0.140472	0.039*	
H37C	0.936859	0.068725	0.094408	0.039*	

### Hexakis(4-methylpiperidinium) tetrakis{tetrachloridoaurate(III)} dichloroiodate(I) chloride (3) Atomic displacement parameters (Å^2^)

**Table:**

	*U*^11^	*U*^22^	*U*^33^	*U*^12^	*U*^13^	*U*^23^
Au1	0.01036 (6)	0.01113 (6)	0.01079 (7)	0.00253 (5)	0.00391 (5)	0.00176 (5)
Au2	0.00919 (9)	0.01244 (9)	0.01023 (9)	0.00297 (7)	0.00296 (7)	0.00280 (7)
Au3	0.01158 (9)	0.01122 (9)	0.01348 (10)	0.00249 (7)	0.00237 (7)	0.00154 (7)
I1	0.01367 (15)	0.01521 (16)	0.01513 (16)	0.00419 (12)	0.00612 (13)	0.00236 (13)
Cl1	0.0109 (4)	0.0199 (5)	0.0197 (5)	0.0047 (3)	0.0027 (3)	0.0052 (4)
Cl2	0.0196 (4)	0.0122 (4)	0.0242 (5)	0.0025 (3)	0.0091 (4)	0.0018 (4)
Cl3	0.0124 (4)	0.0202 (5)	0.0195 (5)	0.0044 (3)	0.0004 (3)	−0.0002 (4)
Cl4	0.0188 (4)	0.0133 (4)	0.0167 (4)	0.0021 (3)	0.0036 (4)	0.0041 (3)
Cl5	0.0172 (4)	0.0141 (4)	0.0195 (4)	0.0045 (3)	0.0085 (4)	0.0053 (4)
Cl6	0.0156 (4)	0.0157 (4)	0.0192 (4)	0.0048 (3)	0.0098 (3)	0.0049 (4)
Cl7	0.0272 (5)	0.0226 (5)	0.0248 (5)	0.0125 (4)	0.0137 (4)	0.0051 (4)
Cl8	0.0224 (5)	0.0181 (5)	0.0215 (5)	0.0064 (4)	0.0077 (4)	0.0092 (4)
Cl9	0.0166 (6)	0.0223 (7)	0.0172 (6)	−0.0019 (5)	0.0075 (5)	−0.0024 (5)
Cl10	0.0201 (5)	0.0213 (5)	0.0197 (5)	0.0029 (4)	0.0070 (4)	0.0004 (4)
N11	0.0135 (16)	0.0191 (17)	0.0196 (17)	0.0037 (13)	0.0073 (13)	0.0014 (14)
C12	0.0169 (18)	0.022 (2)	0.0154 (19)	0.0087 (16)	0.0014 (15)	0.0101 (16)
C13	0.024 (2)	0.0141 (18)	0.019 (2)	0.0083 (15)	0.0088 (16)	0.0079 (15)
C14	0.0170 (18)	0.0135 (18)	0.0169 (19)	0.0044 (14)	0.0099 (15)	0.0044 (15)
C15	0.0140 (17)	0.0161 (18)	0.0149 (18)	0.0022 (14)	0.0021 (14)	0.0038 (15)
C16	0.0137 (17)	0.0177 (19)	0.0150 (18)	0.0035 (14)	0.0012 (14)	0.0084 (15)
C17	0.025 (2)	0.017 (2)	0.033 (2)	−0.0043 (16)	0.0156 (18)	0.0021 (18)
N21	0.0120 (15)	0.0150 (16)	0.0161 (16)	0.0028 (12)	0.0051 (13)	0.0001 (13)
C22	0.0125 (17)	0.0182 (19)	0.0186 (19)	0.0015 (15)	0.0032 (15)	0.0007 (15)
C23	0.0178 (19)	0.0108 (17)	0.025 (2)	0.0011 (14)	0.0084 (16)	0.0019 (15)
C24	0.0196 (19)	0.0165 (18)	0.0180 (19)	0.0076 (15)	0.0110 (15)	0.0055 (15)
C25	0.0110 (17)	0.026 (2)	0.021 (2)	0.0011 (15)	0.0079 (15)	0.0025 (17)
C26	0.0152 (18)	0.0168 (19)	0.023 (2)	−0.0040 (15)	0.0098 (16)	−0.0013 (16)
C27	0.028 (2)	0.031 (2)	0.038 (3)	0.0194 (19)	0.020 (2)	0.015 (2)
N31	0.0156 (16)	0.0180 (17)	0.0274 (19)	0.0083 (14)	0.0055 (14)	0.0012 (15)
C32	0.021 (2)	0.036 (2)	0.024 (2)	0.0089 (18)	0.0130 (17)	0.0058 (19)
C33	0.0156 (18)	0.020 (2)	0.0158 (19)	0.0003 (15)	0.0033 (15)	0.0064 (16)
C34	0.0110 (17)	0.0158 (18)	0.0170 (19)	0.0018 (14)	0.0014 (14)	−0.0005 (15)
C35	0.0182 (19)	0.024 (2)	0.0190 (19)	0.0027 (16)	0.0109 (16)	0.0073 (16)
C36	0.019 (2)	0.022 (2)	0.032 (2)	0.0027 (16)	0.0071 (17)	0.0152 (18)
C37	0.019 (2)	0.023 (2)	0.029 (2)	0.0061 (17)	0.0028 (17)	−0.0010 (18)

### Hexakis(4-methylpiperidinium) tetrakis{tetrachloridoaurate(III)} dichloroiodate(I) chloride (3) Geometric parameters (Å, º)

**Table:**

Au1—Cl1	2.2733 (8)	C22—C23	1.509 (5)
Au1—Cl4	2.2792 (9)	C22—H22A	0.9900
Au1—Cl2	2.2882 (9)	C22—H22B	0.9900
Au1—Cl3	2.3003 (8)	C23—C24	1.533 (5)
Au2—Cl5	2.2794 (8)	C23—H23A	0.9900
Au2—Cl5^i^	2.2794 (8)	C23—H23B	0.9900
Au2—Cl6	2.3052 (8)	C24—C27	1.527 (5)
Au2—Cl6^i^	2.3052 (8)	C24—C25	1.528 (5)
Au3—Cl7^ii^	2.2808 (9)	C24—H24	1.0000
Au3—Cl7	2.2808 (9)	C25—C26	1.516 (5)
Au3—Cl8	2.2837 (9)	C25—H25A	0.9900
Au3—Cl8	2.2837 (9)	C25—H25B	0.9900
I1—Cl10	2.5574 (9)	C26—H26A	0.9900
I1—Cl10^iii^	2.5574 (10)	C26—H26B	0.9900
N11—C12	1.489 (5)	C27—H27A	0.9800
N11—C16	1.496 (4)	C27—H27B	0.9800
N11—H01	0.912 (19)	C27—H27C	0.9800
N11—H02	0.915 (19)	N31—C36	1.487 (5)
C12—C13	1.515 (5)	N31—C32	1.489 (5)
C12—H12A	0.9900	N31—H05	0.907 (19)
C12—H12B	0.9900	N31—H06	0.908 (19)
C13—C14	1.531 (5)	C32—C33	1.514 (5)
C13—H13A	0.9900	C32—H32A	0.9900
C13—H13B	0.9900	C32—H32B	0.9900
C14—C17	1.521 (5)	C33—C34	1.521 (5)
C14—C15	1.528 (5)	C33—H33A	0.9900
C14—H14	1.0000	C33—H33B	0.9900
C15—C16	1.511 (5)	C34—C37	1.530 (5)
C15—H15A	0.9900	C34—C35	1.533 (5)
C15—H15B	0.9900	C34—H34	1.0000
C16—H16A	0.9900	C35—C36	1.506 (5)
C16—H16B	0.9900	C35—H35A	0.9900
C17—H17A	0.9800	C35—H35B	0.9900
C17—H17B	0.9800	C36—H36A	0.9900
C17—H17C	0.9800	C36—H36B	0.9900
N21—C26	1.488 (4)	C37—H37A	0.9800
N21—C22	1.493 (4)	C37—H37B	0.9800
N21—H03	0.909 (19)	C37—H37C	0.9800
N21—H04	0.910 (19)		
			
Cl1—Au1—Cl4	89.87 (3)	C22—C23—C24	111.9 (3)
Cl1—Au1—Cl2	90.75 (3)	C22—C23—H23A	109.2
Cl4—Au1—Cl2	177.70 (3)	C24—C23—H23A	109.2
Cl1—Au1—Cl3	179.16 (3)	C22—C23—H23B	109.2
Cl4—Au1—Cl3	89.40 (3)	C24—C23—H23B	109.2
Cl2—Au1—Cl3	89.96 (3)	H23A—C23—H23B	107.9
Cl5—Au2—Cl5^i^	180.0	C27—C24—C25	111.8 (3)
Cl5—Au2—Cl6	91.51 (3)	C27—C24—C23	111.3 (3)
Cl5^i^—Au2—Cl6	88.48 (3)	C25—C24—C23	108.9 (3)
Cl5—Au2—Cl6^i^	88.49 (3)	C27—C24—H24	108.3
Cl5^i^—Au2—Cl6^i^	91.52 (3)	C25—C24—H24	108.3
Cl6—Au2—Cl6^i^	180.0	C23—C24—H24	108.3
Cl7^ii^—Au3—Cl7	180.00 (4)	C26—C25—C24	112.2 (3)
Cl7^ii^—Au3—Cl8^ii^	90.28 (3)	C26—C25—H25A	109.2
Cl7—Au3—Cl8^ii^	89.72 (3)	C24—C25—H25A	109.2
Cl7^ii^—Au3—Cl8	89.72 (3)	C26—C25—H25B	109.2
Cl7—Au3—Cl8	90.28 (3)	C24—C25—H25B	109.2
Cl8^ii^—Au3—Cl8	180.0	H25A—C25—H25B	107.9
Cl10—I1—Cl10^iii^	180.0	N21—C26—C25	109.7 (3)
C12—N11—C16	111.1 (3)	N21—C26—H26A	109.7
C12—N11—H01	111 (3)	C25—C26—H26A	109.7
C16—N11—H01	109 (3)	N21—C26—H26B	109.7
C12—N11—H02	110 (2)	C25—C26—H26B	109.7
C16—N11—H02	108 (2)	H26A—C26—H26B	108.2
H01—N11—H02	107 (3)	C24—C27—H27A	109.5
N11—C12—C13	110.0 (3)	C24—C27—H27B	109.5
N11—C12—H12A	109.7	H27A—C27—H27B	109.5
C13—C12—H12A	109.7	C24—C27—H27C	109.5
N11—C12—H12B	109.7	H27A—C27—H27C	109.5
C13—C12—H12B	109.7	H27B—C27—H27C	109.5
H12A—C12—H12B	108.2	C36—N31—C32	111.7 (3)
C12—C13—C14	112.1 (3)	C36—N31—H05	110 (3)
C12—C13—H13A	109.2	C32—N31—H05	110 (3)
C14—C13—H13A	109.2	C36—N31—H06	107 (2)
C12—C13—H13B	109.2	C32—N31—H06	110 (2)
C14—C13—H13B	109.2	H05—N31—H06	108 (3)
H13A—C13—H13B	107.9	N31—C32—C33	110.3 (3)
C17—C14—C15	110.7 (3)	N31—C32—H32A	109.6
C17—C14—C13	111.2 (3)	C33—C32—H32A	109.6
C15—C14—C13	109.7 (3)	N31—C32—H32B	109.6
C17—C14—H14	108.4	C33—C32—H32B	109.6
C15—C14—H14	108.4	H32A—C32—H32B	108.1
C13—C14—H14	108.4	C32—C33—C34	112.3 (3)
C16—C15—C14	112.5 (3)	C32—C33—H33A	109.1
C16—C15—H15A	109.1	C34—C33—H33A	109.1
C14—C15—H15A	109.1	C32—C33—H33B	109.1
C16—C15—H15B	109.1	C34—C33—H33B	109.1
C14—C15—H15B	109.1	H33A—C33—H33B	107.9
H15A—C15—H15B	107.8	C33—C34—C37	110.3 (3)
N11—C16—C15	110.5 (3)	C33—C34—C35	110.1 (3)
N11—C16—H16A	109.6	C37—C34—C35	110.9 (3)
C15—C16—H16A	109.6	C33—C34—H34	108.4
N11—C16—H16B	109.6	C37—C34—H34	108.5
C15—C16—H16B	109.6	C35—C34—H34	108.4
H16A—C16—H16B	108.1	C36—C35—C34	112.6 (3)
C14—C17—H17A	109.5	C36—C35—H35A	109.1
C14—C17—H17B	109.5	C34—C35—H35A	109.1
H17A—C17—H17B	109.5	C36—C35—H35B	109.1
C14—C17—H17C	109.5	C34—C35—H35B	109.1
H17A—C17—H17C	109.5	H35A—C35—H35B	107.8
H17B—C17—H17C	109.5	N31—C36—C35	110.4 (3)
C26—N21—C22	111.7 (3)	N31—C36—H36A	109.6
C26—N21—H03	112 (2)	C35—C36—H36A	109.6
C22—N21—H03	108 (2)	N31—C36—H36B	109.6
C26—N21—H04	110 (3)	C35—C36—H36B	109.6
C22—N21—H04	108 (3)	H36A—C36—H36B	108.1
H03—N21—H04	107 (3)	C34—C37—H37A	109.5
N21—C22—C23	110.3 (3)	C34—C37—H37B	109.5
N21—C22—H22A	109.6	H37A—C37—H37B	109.5
C23—C22—H22A	109.6	C34—C37—H37C	109.5
N21—C22—H22B	109.6	H37A—C37—H37C	109.5
C23—C22—H22B	109.6	H37B—C37—H37C	109.5
H22A—C22—H22B	108.1		
			
C16—N11—C12—C13	−59.4 (4)	C27—C24—C25—C26	178.0 (3)
N11—C12—C13—C14	57.1 (4)	C23—C24—C25—C26	54.7 (4)
C12—C13—C14—C17	−176.0 (3)	C22—N21—C26—C25	58.5 (4)
C12—C13—C14—C15	−53.2 (4)	C24—C25—C26—N21	−57.1 (4)
C17—C14—C15—C16	175.6 (3)	C36—N31—C32—C33	−58.8 (4)
C13—C14—C15—C16	52.5 (4)	N31—C32—C33—C34	55.7 (4)
C12—N11—C16—C15	58.7 (4)	C32—C33—C34—C37	−174.8 (3)
C14—C15—C16—N11	−55.6 (4)	C32—C33—C34—C35	−52.0 (4)
C26—N21—C22—C23	−58.6 (4)	C33—C34—C35—C36	51.9 (4)
N21—C22—C23—C24	56.6 (4)	C37—C34—C35—C36	174.3 (3)
C22—C23—C24—C27	−178.0 (3)	C32—N31—C36—C35	58.6 (4)
C22—C23—C24—C25	−54.3 (4)	C34—C35—C36—N31	−55.2 (4)

Symmetry codes: (i) −*x*+2, −*y*+1, −*z*+1; (ii) −*x*+1, −*y*, −*z*; (iii) −*x*+2, −*y*+2, −*z*+1.

### Hexakis(4-methylpiperidinium) tetrakis{tetrachloridoaurate(III)} dichloroiodate(I) chloride (3) Hydrogen-bond geometry (Å, º)

**Table:**

*D*—H···*A*	*D*—H	H···*A*	*D*···*A*	*D*—H···*A*
N11—H01···Cl9	0.91 (2)	2.41 (2)	3.270 (3)	157 (3)
N11—H02···Cl2	0.92 (2)	2.98 (3)	3.479 (3)	116 (2)
N11—H02···Cl10	0.92 (2)	2.58 (2)	3.465 (3)	164 (3)
N21—H03···Cl3	0.91 (2)	2.82 (3)	3.317 (3)	116 (3)
N21—H03···Cl5	0.91 (2)	2.72 (3)	3.402 (3)	133 (3)
N21—H03···Cl6^i^	0.91 (2)	2.79 (2)	3.547 (3)	142 (3)
N21—H04···Cl2	0.91 (2)	2.83 (3)	3.566 (3)	139 (3)
N21—H04···Cl10	0.91 (2)	2.63 (3)	3.371 (3)	139 (3)
N31—H05···Cl3	0.91 (2)	2.92 (3)	3.569 (3)	130 (3)
N31—H05···Cl9	0.91 (2)	2.83 (3)	3.538 (3)	136 (3)
N31—H06···Cl8	0.91 (2)	2.54 (2)	3.406 (3)	161 (3)
C12—H12*A*···Cl7^iv^	0.99	2.96	3.724 (3)	135
C12—H12*A*···Cl8^iv^	0.99	2.80	3.625 (4)	141
C12—H12*B*···Cl4^iv^	0.99	2.82	3.482 (4)	125
C15—H15*B*···Cl5	0.99	2.97	3.508 (4)	115
C16—H16*A*···Cl1^v^	0.99	2.80	3.649 (4)	145
C16—H16*B*···Cl3	0.99	2.85	3.627 (4)	136
C16—H16*B*···Cl5	0.99	2.95	3.659 (4)	129
C22—H22*A*···I1	0.99	3.32	4.155 (4)	144
C22—H22*B*···Cl4^vi^	0.99	2.96	3.847 (4)	149
C25—H25*A*···Cl5^vii^	0.99	2.88	3.823 (4)	160
C32—H32*B*···Cl3	0.99	2.98	3.480 (4)	113
C35—H35*B*···Cl8	0.99	2.89	3.708 (4)	140
C36—H36*B*···Cl1^iv^	0.99	2.86	3.671 (4)	140

Symmetry codes: (i) −*x*+2, −*y*+1, −*z*+1; (iv) −*x*+1, −*y*+1, −*z*; (v) *x*+1, *y*, *z*; (vi) −*x*+1, −*y*+1, −*z*+1; (vii) *x*−1, *y*, *z*.
